# Scrutinizing the Gateway Relationship Between Gaming and Gambling Disorder: Scoping Review With a Focus on the Southeast Asian Region

**DOI:** 10.2196/59740

**Published:** 2025-01-15

**Authors:** Kristiana Siste, Daniel L King, Enjeline Hanafi, Lee Thung Sen, Adrian Adrian, Belinda Julivia Murtani

**Affiliations:** 1 Department of Psychiatry, Faculty of Medicine, Universitas Indonesia – Dr. Cipto Mangunkusumo National Referral Hospital, Jakarta, Indonesia; 2 College of Education, Psychology and Social Work, Flinders University, Adelaide, Australia; 3 School of Psychology, The University of Adelaide, Adelaide, Australia

**Keywords:** behavioral addiction, convergence, gateway effect, gambling advertisement, gamblification, monetized gaming

## Abstract

**Background:**

The gaming and gambling overlap has intensified with new evidence emerging. However, the relationship between gaming and gambling in the digital space is still inconclusive, especially in resource-limited Asian countries.

**Objective:**

This study aims to review available evidence on the possible interaction and focuses specifically on the gateway interaction between gambling and gaming. Additionally, this review delves into the state of evidence from the Southeast Asian region, providing an in-depth analysis of this underexplored area.

**Methods:**

We performed a scoping review by sifting through the publications in five databases. We focused on the gateway interaction and provided a possible pathway model, while two other convergence relationships were provided for comparison.

**Results:**

The scoping review identified a total of 289 publications, with the majority being empirical (n=181), although only 12 studies used longitudinal designs. A significant proportion of the publications (n=152) concentrated on the correlation or comorbidity between gaming and gambling. Most of the evidence has originated from Global North countries, with very limited research emerging from Southeast Asia (n=8). The most commonly studied gambling-like element in video games was loot boxes (n=105). Other elements investigated included esports betting, skin betting, token wagering, gambling advertisements, and gambling-like features. Several longitudinal studies have highlighted the risk of the gateway effect associated with gamblification involvement. However, emerging evidence suggests more nuanced underlying mechanisms that drive the transition from gaming to gambling.

**Conclusions:**

Overall, there is early evidence of linkage between gambling and gaming, through shared structural and biopsychosocial characteristics. This association possibly extends beyond disparate comorbidity, as such engagement in one activity might influence the risk of partaking in the other behavior. The field requires further longitudinal data to determine the directionality and significant precipitating factors of the gateway effect, particularly evidence from Asia.

## Introduction

Video games have undergone substantial evolution since their integration with internet-based platforms, with a notable shift being the monetization of virtual items through chance-based activities [1]. Among the most widely debated mechanics is the “loot box” system [2]. This system permits players to purchase boxes containing randomized items of varying rarity, functionality, and monetary value. Highly coveted items, which enhance gameplay or provide unique aesthetic modifications, are often accessible exclusively through loot boxes [2,3]. Although the concept of probabilistic rewards for in-game items has been a feature of video games since the early days of role-playing games, it is evident that such mechanics have become increasingly monetized by capitalizing on the psychological loop between probability and instant gratification [4].

In addition to loot boxes, numerous games have implemented other forms of microtransactions, including downloadable content, purchasable upgrades, and play passes, which can be acquired with real-world money or via intermediary in-game currencies [2,5]. The gambling-like features (GLFs) of video games have expanded to include both players and spectators, notably through “skin betting” and esports wagering. Esports, much as traditional sports, now allow spectators to place bets on teams and players [6], with websites emerging that facilitate match tracking and performance analysis (eg, Oddspedia). A survey conducted in the United Kingdom revealed that 8.5% of adults had engaged in betting on esports [7], while an Australian study found that 41.5% of respondents participated in esports cash betting, skin betting, or skin gambling [8]. Other platforms have broadened the scope of betting to include not only professional esports leagues but also everyday matches involving skilled players, with transactions occurring in both real-world currency and virtual items or skins, the latter referred to as “skin betting.”

The *ICD-11* (*International Classification of Diseases, 11th Revision*) recently classified gaming and gambling disorders as conditions related to addictive behaviors, underscoring shared diagnostic criteria and symptomatology [9]. Global data indicate a past-year prevalence of gambling disorder ranging from 0.12% to 5.8% [10], while the prevalence of gaming disorder stands at approximately 3.3% [11]. Prior research has estimated the comorbidity between gaming and gambling disorders at approximately 15%. Moreover, emerging evidence has suggested a potential “gateway” effect, where individuals transition from gaming to gambling disorder [12,13]. However, contemporary discourse in this nascent field has shifted toward examining the underlying motivations that drive the initiation and persistence of the transition between the two disorders. It is also plausible that certain gamer populations are more frequently sensitized to gambling stimuli, partly due to the proliferation of targeted gambling advertisements—similar to those promoting other addictive substances [14] and particularly within Southeast Asia [15]. This sensitization places vulnerable adolescents and young adults at elevated risk for problematic gambling behaviors [16]. The increasingly aggressive nature of gambling advertisements, coupled with inadequate regulatory oversight, exacerbates this issue. The paucity of clinical evidence, particularly concerning the gateway effect, presents therapeutic challenges and underscores the urgent need for further research [17].

This study seeks to explore the forms of intersection between gaming and gambling disorders. To achieve this, we conducted a scoping review to systematically evaluate the existing evidence on the overlap between gaming and gambling disorders. The scoping review method was selected, as it offers an exploratory overview of the available literature [18], allowing for the identification of gaps in research. Our scoping review attempts to focus on the gateway effect, while also examining other associations, that is, embedded gambling mechanics within digital games and the clinical comorbidity between the two disorders.

## Methods

### Scoping Review Objectives and Outline

This scoping review was conceptualized as an exploratory approach to research synthesis, aimed at identifying key evidence and theoretical concepts within the field. While several significant areas were included in the search, the examination of gambling mechanics and clinical comorbidity served primarily as background, providing context for the current state of research on the gateway effect. The primary focus of the discussion is the gateway effect, specifically the transition from gaming to gambling disorder. In contrast to a systematic review, this study was not preregistered with a review registry [19,20]. Nevertheless, the review was conducted following the guidelines outlined in the PRISMA-ScR (Preferred Reporting Items for Systematic Reviews and Meta-Analyses Extension for Scoping Reviews) checklist [18,21].

### Search Strategy and Reference Screening

The search strategy focused on systematically examining existing literature on the shared mechanics between gaming and gambling, the concept of gaming as a “gateway” to gambling disorder, and the co-occurrence or comorbidity of gaming and gambling disorders. Detailed information regarding the search methodology is provided in Multimedia Appendix 1. To ensure thoroughness, the search incorporated terms from relevant medical subject headings. Databases including PubMed, ScienceDirect, ProQuest, Google Scholar, and Garuda (the Indonesian national scientific database) were searched using this strategy from their inception until July 31, 2024, with the search results summarized in the *Results* section. Following prior recommendations, the first 300 results from Google Scholar were included [22]. Additionally, a backward citation search was conducted to ensure the comprehensiveness of the studies identified. The results from the database search and the backward citation search were exported into a reference management software (EndNote, version 20, Clarivate).

### Inclusion and Exclusion Criteria

This review encompassed all study types, including commentaries, editorials, letters, and reports [23], to capture the breadth of available evidence and highlight areas requiring further research. The inclusion criteria for nonempirical studies required a discussion of gaming and gambling characteristics. Empirical studies must include statistical data, such as overlapping prevalences, correlations, associations, regression analyses, or other methods that establish a relationship between gaming and gambling. Textbox 1 provides a comprehensive outline of the inclusion and exclusion criteria. The terms “video game(-ing)” and “game(-ing)” were defined based on previous research as “playable interactive digital entertainment that requires audiovisual apparatus, typically demands strategic play, and may involve narratives” [24,25] to avoid confusion stemming from the interchangeable use of “gaming” and “gambling” in some legal contexts [26]. Furthermore, this scoping defines gateway effect as the transitory relationship from video gaming to gambling and reverse gateway effect being the vice versa (gambling to video gaming) [27]. Other definitions of gateway effect are beyond the scope of this scoping review. This paper explored multiple facets of video gaming that might propagate such effects including, but not limited to, microtransactions (ie, in-game payments made available from indirect virtual money or directly through real-life currencies [17]), loot boxes (ie, purchasable content that will give chance-based items [28]), and gambling advertising in video games. Titles and abstracts were screened for eligibility based on inclusion and exclusion criteria by two authors (LTS and AA), with disagreements resolved by a third author (EH). Full-text analysis and data extraction were conducted by two authors (LTS and AA) and subsequently verified by a third author (KS) to ensure methodological rigor. The overall extracted data and interpretation were reviewed by KS and DLK.

Textbox 1. **Criteria for inclusion and exclusion of publications for review.**

**Inclusion criteria**

- This review includes all types of published studies, such as commentaries, editorials, viewpoints, proceedings, reports, empirical studies, and theses.
- Nonempirical studies should discuss the relationship between gaming and gambling characteristics, including converging mechanics (structural features), combined epidemiological or natural histories, risk correlations, or predictive risks.
- Empirical studies should provide statistical data on the overlapping prevalence, clinical profiles, correlations, associations, regression analyses, or other analytical methods that measure the relationship between gaming and gambling.
- Accessible full-text records.

**Exclusion criteria**

- Studies that focus solely on gaming, without any direct reference to gambling elements or gambling disorder.
- Studies that focus solely on gambling, such as electronic gambling machines, video lottery terminals, cyber gambling, online gambling, and other forms of simulated gambling, without any direct reference to video gaming or gaming disorder.
- Studies that measure gaming disorder using nonspecific screening tools, such as those designed to assess general internet addiction, rather than tools specifically developed for gaming disorder.
- Studies published in languages other than English or Bahasa Indonesia.
- Studies that focus exclusively on comparing the adoption of diagnostic criteria, either *ICD-11* (*International Classification of Diseases, Eleventh Revision*) or *DSM-5* (*Diagnostic and Statistical Manual of Mental Disorders, Fifth Edition*).

### Data Extraction

The data extraction method was designed to synthesize evidence in alignment with this study’s key research questions. Following the updated guidelines from JBI (Joanna Briggs Institute) for scoping reviews [18], as well as other relevant recommendations [19,21], and drawing from a prior scoping study [29], a comprehensive set of extraction variables was developed and organized into an Excel (Microsoft Corp) workbook. These variables aim to capture the types of studies, year of publication (prioritizing the date of first digital publication), study location (based on the sample rather than author affiliation), study design and temporality, objectives, study description, the relationship between gaming and gambling, and key findings.

Studies were classified into three categories: reviews (including systematic reviews, scoping reviews, literature reviews, narrative reviews, book chapters, and review reports), empirical studies (encompassing any primary qualitative or quantitative designs, as well as meta-analyses), and viewpoint or commentary pieces (including opinion papers, editorials, comments, and viewpoints). The overlap between gaming and gambling was further categorized into three distinct groups based on this study’s focus: (1) converging mechanics (where the papers examined the shared mechanics between video games and gambling), (2) correlation or comorbidity (where the papers analyzed the overlap or association between problematic gaming and gambling behaviors), and (3) gateway effect (where the papers assessed the predictive relationship between gaming disorder, its gambling-like mechanics, and the development or escalation of gambling behavior). Findings were reported as descriptive data, in line with the recommended guideline [18].

### Ethical Considerations

This scoping review does not require ethical clearance as it reviews published papers.

## Results

### Publication Characteristics

**Figure 1.**
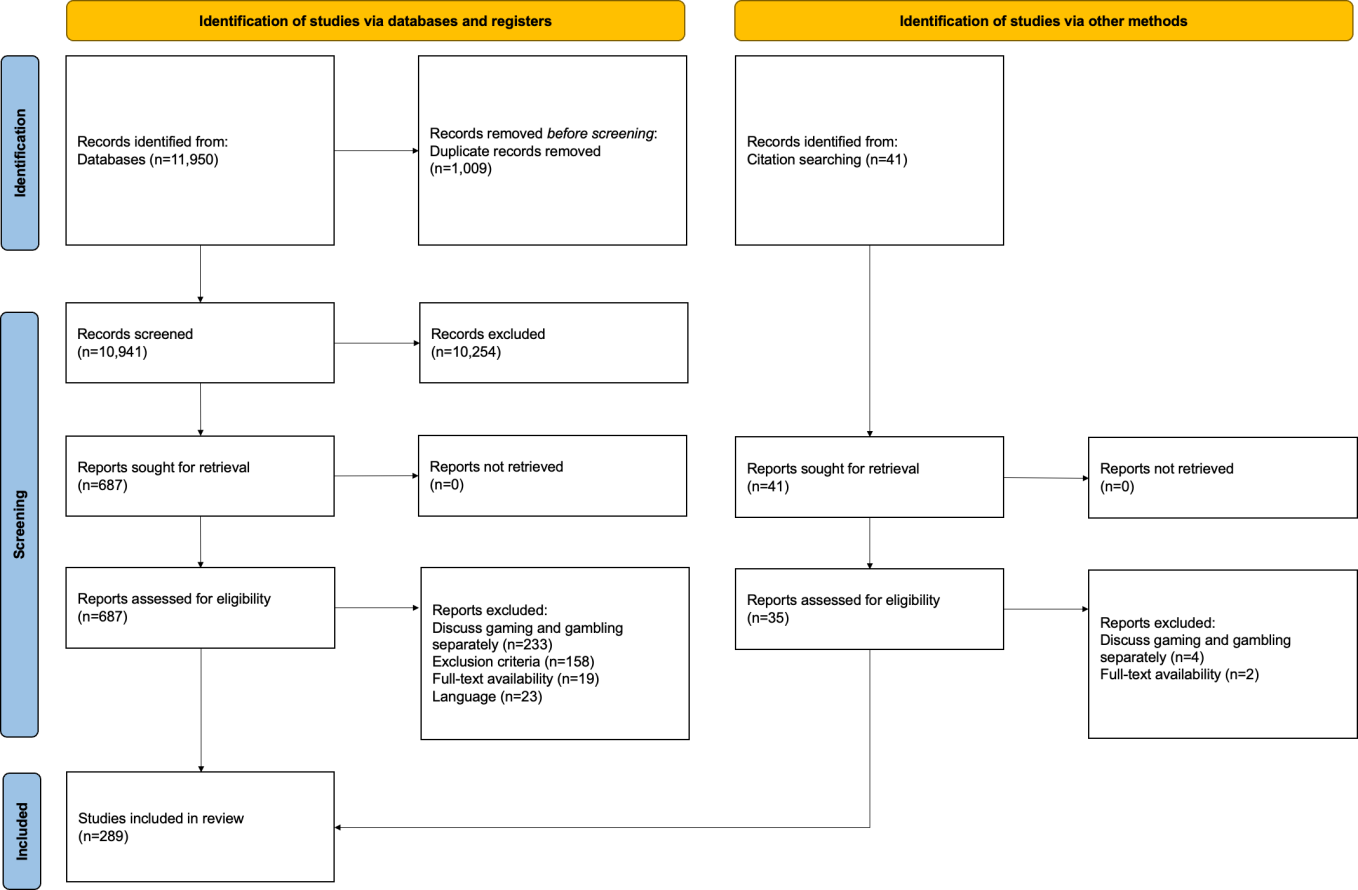
PRISMA-ScR flow diagram. The search across five databases initially yielded 11,950 publications. After removing 1009 duplicates, 10,254 records were excluded for not meeting the inclusion or exclusion criteria during the screening process. Subsequently, 687 publications were retrieved for full-text review. Additionally, 41 records were identified through reference back-searching. Finally, a total of 289 publications were included in this review. PRISMA-ScR: Preferred Reporting Items for Systematic Reviews and Meta-Analyses Extension for Scoping Reviews.

**Figure 2.**
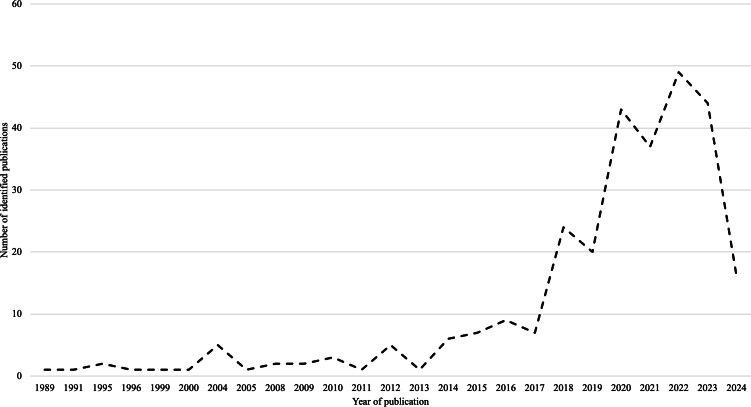
Number of identified publications across the years. This scoping review identified a total of 289 published records spanning from 1989 to 2024. An increasing trend in the number of publications has been observed since 2013, with the peak occurring in 2022 when 49 publications were recorded.

The search strategy and inclusion or exclusion criteria yielded 289 relevant publications (see Multimedia Appendix 2). Figure 1 presents a detailed PRISMA-ScR flow diagram of the database search process. The earliest publication identified dates back to 1989, with an upward trend in research addressing gaming and gambling convergence observed since then (Figure 2), culminating in a peak of 49 publications in 2022. Publications spanned at least 36 countries (excluding those with multinational affiliations), with the highest numbers originating from the United Kingdom (n=53), Australia (n=52), and Canada (n=30). In Southeast Asia, this scoping review identified 5 studies from Indonesia, 2 from Singapore, and 1 from Malaysia. Of the 5 studies from Indonesia, only 2 are empirical [30,31], while the remaining studies consist of commentaries and reviews that examine the relationship between gaming and gambling in the context of Indonesian or Islamic law [32-34].

Among the studies analyzed, 181 are empirical, 69 are review publications, and 39 are viewpoint or commentary pieces. Sample sizes in the reviewed studies varied significantly, ranging from a single-patient case study to a large-scale study involving 16,196 participants. The majority of empirical studies (n=101) used adult samples (defined as aged older than 18 years), while 19.3% (n=35) focused on children and adolescents, and 14.4% (n=26) included both adults and adolescents. Approximately 10.5% (n=19) of the empirical studies either did not involve human participants or did not report age-specific data.

### Gaming-Gambling Interactions Overview

Looking at the area of convergence, 152 (55.5%) of publications examined the correlation or comorbidity between gaming and gambling, 35% (n=96) assessed the converging mechanics of video game gamblification, and 15% (n=41) explored the gateway effect of gaming to gambling. Gaming and gambling comorbidity ranged from 1.2% to 26.3% [35-38] and varying correlations, from weak [39,40], moderate [41,42] to strong [43-45]. Out of all identified publications, the form of microtransactions most studied was loot box (n=105), followed by esports betting (n=49), and then skins betting (n=29). Twenty-three studies focused on GLFs, 11 studies specifically examined the effects of gambling advertisements in and around video games, and 3 studies described token wagering. Contrastingly, several new research indicated nonsignificant association between loot box involvement and problem gambling [46-48]. Cryptogames have only recently emerged [49,50] representing a new frontier in the overlap between gaming and gambling. Some view cryptogames to be gambling-like activities, as they blend elements of skill and chance and are inherently linked to cryptocurrency and monetary liquefaction [49].

### Longitudinal Studies

Out of all the empirical studies, 12 publications used a longitudinal design. Of the 12 longitudinal publications, five publications [51-55] focused on adolescent samples and scrutinized the gateway effect. These longitudinal studies on adolescents examined baseline involvement with microtransactions (eg, loot boxes and virtual currencies) and exposure to gambling advertisements. All studies found that these baseline factors significantly predicted the development of problematic gambling over time. Alternatively, 7 publications concentrated on the adult population [27,48,56-60]. However, only 4 investigated the gateway effect [27,48,59,60] focusing on baseline involvement to loot boxes, gambling advertisements, skin betting, and problematic gaming. Out of those 4 studies, 2 reported significant gaming to gambling transition [27,60], while the other 2 studies did not find significant gateway effect [48,59]. Furthermore, 2 longitudinal studies investigated both gateway and reverse gateway effect. Both studies reported that only the gateway effect demonstrated statistical significance, while the reverse gateway effect did not [27,53].

### Risk Factors to Gateway Effect

#### Biological

Demographically, the younger populations are at greater risk for both gaming and gambling disorders. Some studies have observed the gateway effect from gaming to gambling disorder to be more frequent among youth [61-63]. In contrast, another group has demonstrated that esports bettors had a later onset age than traditional sports bettors and carry higher odds for problematic gambling [64]. Multiple studies indicate that male sex may be a risk factor for developing the gateway effect [28,62,65], though one study exhibited its potential as a protective factor for the gateway effect [53]. Paradoxically, emerging research suggest that female sex may be associated with an increased risk, particularly in the post-COVID-19 period [55,66,67].

#### Psychosocial Factors

Psychologically, novelty-seeking temperament, compulsivity, and impulsivity [68-70] are correlated to interacting with gambling features in video games, although findings have been inconsistent. The gateway effect was also observed to be predisposed by cognitive errors [28,47,54], for example, predictive bias, sunk-cost bias, or expectancy. A study by Spicer et al [47] found that the transition from gaming to gambling is more accurately explained by the connection between gaming behaviors and gambling-related cognitions, rather than solely through involvement with loot boxes. Several studies proposed simulated gambling in social networking applications and having peers making similar microtransactions [54,71]. Additionally, a greater risk of developing pathological gambling was seen for gamers with average income but higher spending [72]. In spite of that, other studies were unable to replicate these findings [73,74].

### Drivers of Gateway Effect

#### Structural Features

Cited intermediary pathways for the transition from gaming to gambling include priming of gambling stimuli that leads to normalization and also desensitization toward monetary losses [28,61]. Several studies [27,52,61] have described affinity, exposure to gambling features in video games, and gambling advertisement as factors increasing the risk, particularly among youth. Frequency of in-game transactions (eg, purchased or sold loot boxes), amount spent monthly, number of opening loot boxes or other microtransactions, and history of participation in traditional gambling were predictive of pathological gambling [52,62,75,76]. An overarching phenomenon, termed GLFs in modern video games, has been described [77-81]. GLFs are manifested as virtual currencies, chance-based purchases, and event features [78,79,81]. Other forms include meta-game or real-life rewards, daily log-in payouts, and near-miss features [79]. Some recent studies also noted the separate impact of gambling advertisements [52,54,82-85], which enhanced gambling interest, engagement in chance-based activities in-game, associated with earlier gambling onset, and higher gambling participation using real-world currency. These gambling advertisements appear on live streaming platforms [86] and social media apps, oftentimes trying to appeal to younger populations [85].

#### Motives for Shifting

Multiple studies have looked into motivations for either using skin or monetary instruments for esports betting [87], involvement in both gaming and gambling [76], using GLFs [31,88,89], and specifically to transition from gaming to gambling [28,90]. Motives for shifting from gaming to gambling include perceived similarity of structure between GLFs and gambling games, thus sense of normalization to shift or even a routine [28]. Players also seek excitement and emotions that are thought to be similar or more intense through gambling activities. Furthermore, they are aware of the addiction potential of GLFs and gambling games. They see gambling as another outlet to channel the compulsion. Subsequently, the positive experience from GLFs form cognitive biases such as illusion of control. Lastly, the video game players view virtual funds or currencies and subsequent real-life rewards as carrying social and financial benefits to help fund their gameplay [90]. Adolescents may view accessibility to underage gambling as a form of social prestige [91].

### Harm and Preventive Measures for Gaming-Gambling Convergence

#### Harms and Consequences

Involvement in microtransactions and gamers with gambling engagement was shown with higher gambling harm categories [92-95]. Gambling with skins rather than traditional monetary form demonstrated a higher degree of experienced harm on the Short Gambling Harm Screen [96]. Experienced harms include financial loss, physical symptoms, psychological harm, social deficits, work or academic distress, or legacy harm [30,96]. For example, Drummond et al [97] reported a high degree of psychological distress among video gamers who purchased loot boxes. Intriguingly, a study in the United States found that students with esports as a career choice experienced stigmatization from school counselors. This might stem from the societal biases on the nontraditional career pathway and the association between esports to addiction, gambling, and overall negative judgment [98].

#### Preventive Measures

Some research has advocated for GLFs in video games to be described and incorporated as criteria for licensure or censorship [46,99]. In line, there are suggestions that this information is disclosed to parents and youth, allowing the making of informed decisions [46]. Some hold the view that parents should be equipped with knowledge about the risks associated with microtransactions, chance-based activities, and gambling imagery in video games [100]. Others have reviewed the potential for implementing several layers of protection, including age checking, payment security measures, and setting a maximum spending limit [101,102]. Several viewpoint papers have also expanded to call for accountability from the gaming and gambling industries [100,103], urging them to engage with regulators on consumption risks. Several jurisdictions have responded by banning specific microtransactions in video games, for instance, loot boxes [104]. A commentary paper had voiced concerns regarding the creation of black markets due to the outright banning of microtransactions [105]. Technically, major video game corporations could also implement access blocks for skins or items on unregulated third-party websites [106].

## Discussion

This scoping review aimed to examine the existing body of literature, map the landscape of available publications, and analyze selected evidence. A specific relationship, known as the “gateway effect,” describes the transition from gaming to gambling disorders [107]. Our findings indicate an increase in studies seeking to clarify this relationship, highlighting the clinical challenges posed by the convergence of gaming and gambling platforms. This overlap potentially heightens awareness of the gateway relationship, leading to increased reports and complex analyses. Overall, the pooled data suggest a small to moderate correlation between gaming and gambling behaviors [108]. However, much of the evidence supporting the gateway effect is derived from cross-sectional studies, limiting causal inference. Additionally, studies varied widely in defining initial pathological gaming behaviors and subsequent problematic gambling activity [8,27,28,53,63,68,69,75,76,92,94,109-111]. For instance, Vadlin et al [53] assessed predictive gaming behaviors using the Gaming Addiction Identification Test, whereas Molde et al [27] used the Gaming Addiction Scale. Exposure to GLFs in nongambling environments may also acclimate individuals to gambling [61,112]. Some recent studies have failed to replicate significant associations between in-game gambling activities (ie, loot boxes) and problematic gambling [46-48]. More nuanced analyses suggest that the association between loot box engagement and problematic gambling may be indirect, driven by gambling-related cognitive biases [47]. In addition, Xiao et al [46] propose that cultural factors, such as limited gambling availability and enforced transparency within the gambling industry, may contribute to the nonsignificant association observed in their Chinese-speaking sample.

Although estimates exist for gaming-gambling comorbidity, the rates of transition via the gateway effect remain unclear. Moreover, despite global reports of higher rates of gaming disorder [113], there is limited data from Asian countries on gaming-gambling convergence. In Indonesia, all forms of gambling, including loot boxes, are prohibited [32], which may contribute to a societal stigma surrounding such behaviors and thus related research. Additionally, Southeast Asian countries tend to prioritize enforcement measures, such as crackdowns on gambling [114], rather than investing in public health research on gambling [115]. In Indonesia, gambling and other behavioral addictions are not covered by the national health insurance system, further contributing to the scarcity of data and limited research in this area. Nonetheless, there has been a recent rise in empirical studies from Indonesia examining gambling elements within online video games [30,31]. The Lancet Public Health Commission’s [116] initiative on global cooperation to address boundary-crossing gambling products should encourage research in Global South countries, such as Indonesia. Enhanced empirical evidence is essential for informing policies in these regions, as unique national gambling cultures [46] and differing jurisdictions [117] have been shown to influence gaming-gambling convergence in various ways.

Some experts suggest that legal age attainment may amplify gambling participation [118], though this factor is inapplicable in countries where gambling is prohibited, such as Indonesia. Additionally, the gateway transition may be encouraged by increased economic access (eg, loans or buy-now-pay-later options [119]) upon reaching adulthood. Furthermore, the transitional mechanism may be reinforced by exposure to gambling advertisements [54]. However, such exposure and initial GLF involvement do not necessarily lead to problematic gambling behaviors. Multiple studies have indicated that additional biopsychosocial vulnerabilities (eg, gambling-related cognitive fallacies) are required for these behaviors to develop [47,48]. Preliminary findings indicate that certain biopsychosocial factors in the gateway effect mirror elements of the Interaction of Person-Affect-Cognition-Execution model [120], which describes risk factors for gaming and gambling disorders independently. These factors include younger age, specific temperamental traits, and impaired cognitive processes. Notably, the evidence linking sex to the gateway effect has become increasingly ambiguous. Female participation in video games and GLFs has been steadily rising [67], which corresponds with a heightened risk of problematic gambling among this demographic. Some researchers propose that this trend may resemble the telescoping phenomenon observed in gambling disorders, where the onset and progression of gambling-related issues occur more rapidly [55]. Concurrently, online gambling advertisements have increasingly targeted female audiences [121]. There is evidence of shared neurobiological disruptions to the prefrontal cortices and striatum, particularly within the default mode and reward circuits, in both gaming and gambling disorders [122]. However, definitive data linking these neural alterations to the gateway effect are still lacking.

Several identified studies also observed that the reverse effect (gambling to gaming) was not statistically significant [27,109,123]. This phenomenon raised the question of whether gambling has higher addictive potency than gaming. Some gamblers engage in video game chance-based activities as they are perceived to be “safer” [28]. Prior gambling participation did correlate to higher microtransaction involvement [124-126]. Moreover, several studies [52,54,82] have discovered the associative link between gambling marketing around video games to gambling behavior, though modest, and gambling industries have used these targeted advertisements to expand microtransactions (eg, loot box) usage [127].

**Figure 3.**
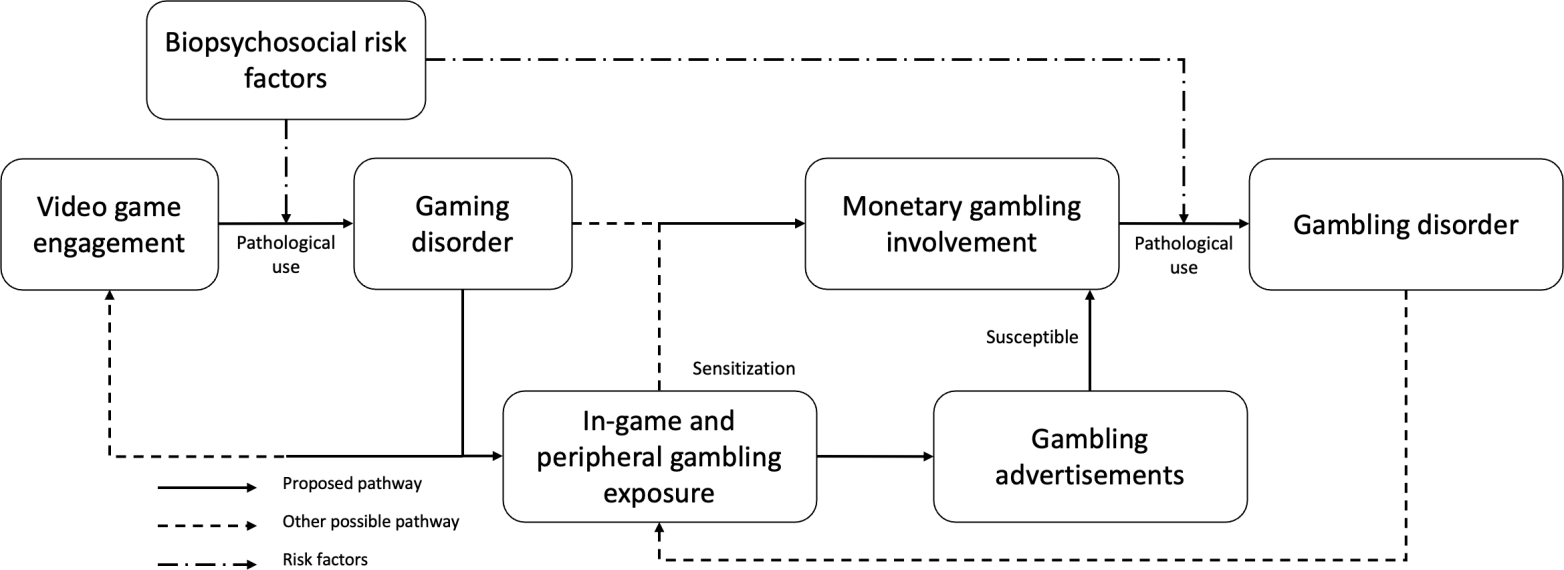
This is a simplified illustration of the dual-hit model proposed for the transition from gaming to gambling. An alternative pathway bypasses the dual-hit approach, where only one of the two hits is experienced. Peripheral exposure includes gambling-related features found on platforms linked to video games, such as, but not limited to, loot box involvement, watching betting activities on video game streaming services, or spectating esports betting. Gambling advertisements may be encountered through pop-up advertisements, video game chat applications, online forums, competitive events, or other evolving forms of interaction.

These patterns might be sensitized with prior engagement to chance-based activities in-game and exposure to gambling advertisements. The two elements might represent a dual-hit pathway for the gateway transition mechanism, similar to the two-hit hypothesis of schizophrenia [128,129] that proposes genetic factors (first hit) sensitize an individual to the environmental insults (second hit) leading to schizophrenia. Adopting the concept, the in-game or peripheral participation in gambling-like activities primes the individual as the first hit, and the gambling advertisements act as the second insult leading to real-world gambling participation (see Figure 3; note that the figure is not meant to be exhaustive). An alternative pathway is presented that might circumvent the dual hit pathway, meaning only one of the two hits is experienced. For instance, the individual progresses from microtransactions to monetary gambling without exposure to gambling advertisements. The proposed dual hit pathway remains a hypothetical and theoretical model requiring empirical evidence to demonstrate if it poses a greater risk than the other pathway.

Consequently, the response to mitigate the migration to gambling behaviors and the associated harms has been insufficient. The rapid evolution of gaming and gambling mechanisms on digital platforms has outpaced regulatory efforts. Historical policy experiences indicate that outright bans may not be the most effective solution [105,127]. Emerging blended forms, such as cryptogames, may also introduce new legal challenges. Instead, industry and structural strategies could encompass implementing more stringent age restrictions [130], restricting API access by third-party platforms [131], instituting additional licensing requirements [104], and promoting self-regulation and ethical conduct within the industry [132]. In 2016, Indonesia introduced its game rating system, which permits gambling-like elements in games rated for players aged 13 years and older, provided they do not include cashing-out features. However, the age threshold should be raised to 18 years, as gambling-like elements present risks and are illegal in the physical realm. Despite the recent increase in research on the gateway effect from gaming to gambling, there remains a significant lack of empirical, clinical, and psychological preventive measures. Early detection should be implemented for all patients with gaming disorder by screening for involvement in microtransactions and other gambling-like activities. This approach will facilitate the application of harm reduction strategies, such as limiting access to real-world money and restricting transactions to virtual currencies that cannot be cashed out. Additionally, engaging families or significant others in the use of supervision applications can help restrict exposure to gambling advertisements. More targeted interventions should focus on clinical management to address cognitive biases and gambling fallacies, fostering awareness that chance-based activities constitute gambling and may have negative consequences.

Overall, the existing studies illustrate that children, adolescents, and emerging adults are particularly vulnerable to the convergence of gaming and gambling. Additional predisposing factors contributing to the transition toward risky behaviors in these groups, such as high impulsivity and sensation-seeking tendencies [76,133], warrant further research. Moreover, additional research is needed to substantiate the gateway effect, aligning with the perspectives presented in previous reviews and commentaries [17,25,107]. Most studies on the gateway effect have focused on traditional Western gambling activities, leaving region-specific gambling activities, such as pachinko in Japan or karambol in Indonesia, relatively underexplored. Further research is essential to clarify the epidemiology, specific mechanisms of transition, directionality of the transition, definitive risk factors, and effective preventive measures related to the gaming-gambling gateway effect.

This study has several limitations. First, the scoping review approach lacks the comprehensiveness of a systematic review, and the identified publications are not exhaustive (for instance, preprint databases were not included, and the search strategy was not preregistered). Consequently, the descriptive counts and proportions presented here reflect only the exploratory sample of identified publications, not the entire body of literature [18]. Second, this study did not address gamified gambling or the growing trend of skill-based gambling forms. Publications focusing solely on social casino gambling were also excluded, as these are traditional gambling activities delivered via the internet rather than video games [107]. Third, this scoping review incorporates some viewpoints and commentary pieces to map the publication landscape. Where feasible, references to these sources have been minimized or clearly identified. Readers are encouraged to be mindful of the varying levels of evidence these pieces provide and to refer to the supplementary materials or reference list for verification if needed. Fourth, the majority of empirical studies rely on self-reported data, which may impact accuracy and necessitates caution in interpretation. Finally, studies that compare gaming and gambling through distinct or combined samples, or with differing diagnostic criteria, were excluded, as such heterogeneity does not support direct examination of overlap or convergence. Nonetheless, these studies still offer valuable insights into the broader relationship between gaming and gambling disorders as behavioral addictions.

## Supplementary material

10.2196/59740 Multimedia Appendix 1 Search strategy.

10.2196/59740 Multimedia Appendix 2 Results of data extraction.

10.2196/59740 Checklist 1 PRISMA-ScR (Preferred Reporting Items for Systematic Reviews and Meta-Analyses Extension for Scoping Reviews) checklist.

## Abbreviations

GLF: gambling-like feature
*ICD-11*: *International Classification of Diseases, 11th Revision*
JBI: Joanna Briggs Institute
PRISMA-ScR: Preferred Reporting Items for Systematic Reviews and Meta-Analyses Extension for Scoping Reviews

## References

[1] King DL, Delfabbro P, Griffiths MD (2010). The convergence of gambling and digital media: implications for gambling in young people. J Gambl Stud.

[2] Drummond A, Sauer JD (2018). Video game loot boxes are psychologically akin to gambling. Nat Hum Behav.

[3] Drummond A, Sauer JD, Hall LC, Zendle D, Loudon MR (2020). Why loot boxes could be regulated as gambling. Nat Hum Behav.

[4] King DL, Gainsbury SM, Delfabbro PH, Hing N, Abarbanel B (2015). Distinguishing between gaming and gambling activities in addiction research. J Behav Addict.

[5] Davidovici-Nora M (2013). Innovation in business models in the video game industry: free-to-play or the gaming experience as a service. Comput Game J.

[6] Bányai F, Griffiths MD, Király O, Demetrovics Z (2019). The psychology of esports: a systematic literature review. J Gambl Stud.

[7] Wardle H, Petrovskaya E, Zendle D (2020). Defining the esports bettor: evidence from an online panel survey of emerging adults. Int Gambl Stud.

[8] Greer N, Rockloff M, Russell AMT (2020). Gambling and video games: are esports betting and skin gambling associated with greater gambling involvement and harm?. https://responsiblegambling.vic.gov.au/resources/publications/gambling-and-video-games-are-esports-betting-and-skin-gambling-associated-with-greater-gambling-involvement-and-harm-967/.

[9] Stein DJ, Szatmari P, Gaebel W (2020). Mental, behavioral and neurodevelopmental disorders in the ICD-11: an international perspective on key changes and controversies. BMC Med.

[10] Calado F, Griffiths MD (2016). Problem gambling worldwide: an update and systematic review of empirical research (2000-2015). J Behav Addict.

[11] Kim HS, Son G, Roh EB (2022). Prevalence of gaming disorder: a meta-analysis. Addict Behav.

[12] Li W, Mills D, Nower L (2019). The relationship of loot box purchases to problem video gaming and problem gambling. Addict Behav.

[13] Macey J, Hamari J (2018). Investigating relationships between video gaming, spectating esports, and gambling. Comput Human Behav.

[14] Siste K, Sen LT (2021). Ascending pattern of alcohol use and underage drinking in Asia: a commentary on the article by Assanangkornchai and Vichitkunakorn (2020). Alcohol Clin Exp Res.

[15] Lala G, Chandra S, Ogun N, Moody L, Third A (2022). Online Safety Perceptions, Needs, and Expectations of Young People in Southeast Asia: Consultations with Young People in Indonesia.

[16] Lussier ID, Derevensky J, Gupta R, Vitaro F (2014). Risk, compensatory, protective, and vulnerability factors related to youth gambling problems. Psychol Addict Behav.

[17] Gibson E, Griffiths MD, Calado F, Harris A (2022). The relationship between videogame micro-transactions and problem gaming and gambling: a systematic review. Comput Hum Behav.

[18] Peters MDJ, Marnie C, Tricco AC (2020). Updated methodological guidance for the conduct of scoping reviews. JBI Evd Synth.

[19] Colquhoun HL, Levac D, O’Brien KK (2014). Scoping reviews: time for clarity in definition, methods, and reporting. J Clin Epidemiol.

[20] Winters N, Langer L, Geniets A (2018). Scoping review assessing the evidence used to support the adoption of mobile health (mHealth) technologies for the education and training of community health workers (CHWs) in low-income and middle-income countries. BMJ Open.

[21] Tricco AC, Lillie E, Zarin W (2018). PRISMA Extension for Scoping Reviews (PRISMA-ScR): checklist and explanation. Ann Intern Med.

[22] Haddaway NR, Collins AM, Coughlin D, Kirk S (2015). The role of Google Scholar in evidence reviews and its applicability to grey literature searching. PLoS One.

[23] Blanco D, Altman D, Moher D, Boutron I, Kirkham JJ, Cobo E (2019). Scoping review on interventions to improve adherence to reporting guidelines in health research. BMJ Open.

[24] Esposito N A short and simple definition of what a videogame is.

[25] King DL, Delfabbro PH (2020). The convergence of gambling and monetised gaming activities. Curr Opin Behav Sci.

[26] Humphreys A, Latour KA (2013). Framing the game: assessing the impact of cultural representations on consumer perceptions of legitimacy. J Consum Res.

[27] Molde H, Holmøy B, Merkesdal AG (2019). Are video games a gateway to gambling? A longitudinal study based on a representative Norwegian sample. J Gambl Stud.

[28] Spicer SG, Fullwood C, Close J, Nicklin LL, Lloyd J, Lloyd H (2022). Loot boxes and problem gambling: investigating the “gateway hypothesis”. Addict Behav.

[29] Montiel I, Basterra-González A, Machimbarrena JM, Ortega-Barón J, González-Cabrera J (2022). Loot box engagement: a scoping review of primary studies on prevalence and association with problematic gaming and gambling. PLoS One.

[30] Lubis AA, Saleh S, Marsa YJ (2022). The phenomenon of online gambling under the guise of online games among college student. J Hum Soc Stud.

[31] Kesuma AE, Princes E (2024). Antecedents of gacha gaming intention: extending UTAUT2 with structural video game characteristics. Comput Hum Behav Rep.

[32] Putra MTR, Gunadi A (2020). Legalitas sistem monetisasi lootbox dalam transaksi game online berdasarkan undang-undang nomor 11 tahun 2008 jo undang-undang nomor 19 tahun 2016. J Huk Adigama.

[33] Budiman R, Romadini NA, Herwandi Aziz MA, Pratama AG (2022). The impact of online gambling among Indonesian teens and technology. ITSDI.

[34] Malik BW, Harisah H (2024). An islamic perspective on e-sport competition. Islamuna J Stud Islam.

[35] Sanders J, Williams R (2017). Factors distinguishing problem gamblers, problem video-gamers, and dual problem gamblers/video-gamers [Abstract]. J Behav Addict.

[36] Peter SC, Li Q, Pfund RA, Whelan JP, Meyers AW (2019). Public stigma across addictive behaviors: casino gambling, esports gambling, and internet gaming. J Gambl Stud.

[37] Szerman N, Basurte-Villamor I, Vega P (2023). Is there such a thing as gambling dual disorder? Preliminary evidence and clinical profiles. Eur Neuropsychopharmacol.

[38] Marmet S, Studer J, Wicki M, Bertholet N, Khazaal Y, Gmel G (2019). Unique versus shared associations between self-reported behavioral addictions and substance use disorders and mental health problems: a commonality analysis in a large sample of young Swiss men. J Behav Addict.

[39] Walther B, Morgenstern M, Hanewinkel R (2012). Co-occurrence of addictive behaviours: personality factors related to substance use, gambling and computer gaming. Eur Addict Res.

[40] Mills DJ, Marchica L, Keough MT, Derevensky JL (2020). Exploring differences in substance use among emerging adults at-risk for problem gambling, and/or problem video gaming. Int Gambl Stud.

[41] Schluter MG, Hodgins DC, Wolfe J, Wild TC (2018). Can one simple questionnaire assess substance-related and behavioural addiction problems? Results of a proposed new screener for community epidemiology. Addiction.

[42] Brooks GA, Clark L (2019). Associations between loot box use, problematic gaming and gambling, and gambling-related cognitions. Addict Behav.

[43] Greenberg JL, Lewis SE, Dodd DK (1999). Overlapping addictions and self-esteem among college men and women. Addict Behav.

[44] Macey J, Abarbanel B, Hamari J (2021). What predicts esports betting? A study on consumption of video games, esports, gambling and demographic factors. New Media Soc.

[45] Spicer SG, Nicklin LL, Uther M, Lloyd J, Lloyd H, Close J (2022). Loot boxes, problem gambling and problem video gaming: a systematic review and meta-synthesis. New Media Soc.

[46] Xiao LY, Fraser TC, Nielsen RKL, Newall PWS (2024). Loot boxes, gambling-related risk factors, and mental health in mainland China: a large-scale survey. Addict Behav.

[47] Spicer SG, Close J, Nicklin LL (2024). Exploring the relationships between psychological variables and loot box engagement, part 2: exploratory analyses of complex relationships. R Soc Open Sci.

[48] D’Amico NJ, Drummond A, de Salas K (2022). No effect of short term exposure to gambling like reward systems on post game risk taking. Sci Rep.

[49] Scholten OJ, Hughes NGJ, Deterding S, Drachen A, Walker JA, Zendle D Ethereum crypto-games: mechanics, prevalence, and gambling similarities.

[50] Serada A Why is cryptokitties (not) gambling?. https://dl.acm.org/doi/proceedings/10.1145/3402942.

[51] Johnson TE, Dixon MR (2009). Influencing children’s pregambling game playing via conditional discrimination training. J Appl Behav Anal.

[52] Hayer T, Kalke J, Meyer G, Brosowski T (2018). Do simulated gambling activities predict gambling with real money during adolescence? Empirical findings from a longitudinal study. J Gambl Stud.

[53] Vadlin S, Åslund C, Nilsson KW (2018). A longitudinal study of the individual- and group-level problematic gaming and associations with problem gambling among Swedish adolescents. Brain Behav.

[54] Brosowski T, Turowski T, Hayer T (2020). Simulated gambling consumption mediation model (SGCMM): disentangling convergence with parallel mediation models. Int Gambl Stud.

[55] González-Cabrera J, Basterra-González A, Ortega-Barón J (2023). Loot box purchases and their relationship with internet gaming disorder and online gambling disorder in adolescents: a prospective study. Comput Hum Behav.

[56] Tárrega S, Castro-Carreras L, Fernández-Aranda F (2015). A serious videogame as an additional therapy tool for training emotional regulation and impulsivity control in severe gambling disorder. Front Psychol.

[57] Jouhki H, Savolainen I, Sirola A, Oksanen A (2022). Escapism and excessive online behaviors: a three-wave longitudinal study in Finland during the COVID-19 pandemic. Int J Environ Res Public Health.

[58] Mohamed MS, Rukh G, Schiöth HB (2023). Worsened anxiety and loneliness influenced gaming and gambling during the COVID-19 pandemic. JCM.

[59] Wardle H, Tipping S (2023). The relationship between problematic gambling severity and engagement with gambling products: longitudinal analysis of the Emerging Adults Gambling Survey. Addiction.

[60] Brooks GA, Clark L (2023). The gamblers of the future? Migration from loot boxes to gambling in a longitudinal study of young adults. Comput Human Behav.

[61] Armstrong T, Rockloff M, Browne M, Li E (2018). An exploration of how simulated gambling games may promote gambling with money. J Gambl Stud.

[62] Greer N, Rockloff MJ, Russell AMT, Lole L (2021). Are esports bettors a new generation of harmed gamblers? A comparison with sports bettors on gambling involvement, problems, and harm. J Behav Addict.

[63] Kristiansen S, Severin MC (2020). Loot box engagement and problem gambling among adolescent gamers: findings from a national survey. Addict Behav.

[64] Gainsbury SM, Abarbanel B, Blaszczynski A (2017). Game on: comparison of demographic profiles, consumption behaviors, and gambling site selection criteria of esports and sports bettors. Gaming Law Rev.

[65] DeCamp W (2021). Loot boxes and gambling: similarities and dissimilarities in risk and protective factors. J Gambl Stud.

[66] Ide S, Nakanishi M, Yamasaki S (2021). Adolescent problem gaming and loot box purchasing in video games: cross-sectional observational study using population-based cohort data. JMIR Serious Games.

[67] DeCamp W, Daly K (2023). Loot box consumption by adolescents pre- and post- pandemic lockdown. PeerJ.

[68] Hing N, Lole L, Russell AMT (2022). Adolescent betting on esports using cash and skins: links with gaming, monetary gambling, and problematic gambling. PLoS One.

[69] Hing N, Rockloff M, Russell AMT (2022). Loot box purchasing is linked to problem gambling in adolescents when controlling for monetary gambling participation. J Behav Addict.

[70] Muela I, Navas JF, Barrada JR (2023). Operationalization and measurement of compulsivity across video gaming and gambling behavioral domains. BMC Psychol.

[71] King DL, Russell AMT, Delfabbro PH, Polisena D (2020). Fortnite microtransaction spending was associated with peers’ purchasing behaviors but not gaming disorder symptoms. Addict Behav.

[72] Close J, Spicer SG, Nicklin LL, Uther M, Lloyd J, Lloyd H (2021). Secondary analysis of loot box data: are high-spending “whales” wealthy gamers or problem gamblers?. Addict Behav.

[73] Close J, Spicer SG, Nicklin LL, Lloyd J, Lloyd H (2022). Loot box engagement: relationships with educational attainment, employment status and earnings in a cohort of 16 000 United Kingdom gamers. Addiction.

[74] von Meduna M, Steinmetz F, Ante L, Reynolds J, Fiedler I (2020). Loot boxes are gambling-like elements in video games with harmful potential: results from a large-scale population survey. Technol Soc.

[75] Wardle H (2019). The same or different? Convergence of skin gambling and other gambling among children. J Gambl Stud.

[76] King A, Wong-Padoongpatt G, Barrita A, Phung DT, Tong T (2020). Risk factors of problem gaming and gambling in us emerging adult non-students: the role of loot boxes, microtransactions, and risk-taking. Issues Ment Health Nurs.

[77] Teichert T, Gainsbury SM, Mühlbach C (2017). Positioning of online gambling and gaming products from a consumer perspective: a blurring of perceived boundaries. Comput Hum Behav.

[78] Imataka G, Izumi S, Miyamoto Y, Maehashi A (2024). Gaming disorders: navigating the fine line between entertainment and addiction-gaming history, health risks, social consequences, and pathways to prevention. J Clin Med.

[79] Saini N, Hodgins DC (2023). Investigating gaming structural features associated with gaming disorder and proposing a revised taxonomical model: a scoping review. J Behav Addict.

[80] Macey J, Hamari J, Adam M (2024). A conceptual framework for understanding and identifying gamblified experiences. Comput Human Behav.

[81] Kim HS, Leslie RD, Stewart SH (2023). A scoping review of the association between loot boxes, esports, skin betting, and token wagering with gambling and video gaming behaviors. J Behav Addict.

[82] Labrador FJ, Bernaldo-de-Quirós M, Sánchez-Iglesias I (2021). Advertising games of chance in adolescents and young adults in Spain. J Gambl Stud.

[83] Russell AMT, Armstrong TA, Rockloff MJ, Greer NM, Hing N, Browne M (2020). Exploring the changing landscape of gambling in childhood, adolescence and young adulthood.

[84] Abarbanel B, Phung D (2019). Exploring gamers’ perceptions of esports betting advertising. Gaming Law Rev.

[85] Rossi R, Nairn A, Smith J, Inskip C (2021). “Get a £10 free bet every week!”—gambling advertising on Twitter: volume, content, followers, engagement, and regulatory compliance. J Public Policy & Marketing.

[86] Abarbanel B, Johnson MR (2020). Gambling engagement mechanisms in Twitch live streaming. Int Gambl Stud.

[87] Denoo M, Bibert N, Zaman B Disentangling the motivational pathways of recreational esports gamblers: a laddering study. https://dl.acm.org/doi/proceedings/10.1145/3411764.

[88] Lakić N, Bernik A, Čep A (2023). Addiction and spending in gacha games. Information.

[89] Bujňáková E (2023). Consumer behavior of league of legends players [Bachelor’s thesis]. http://www.theseus.fi/handle/10024/812974.

[90] Wieczorek Ł, Bujalski M, Dąbrowska K (2024). “I can tell you it’s a bit of a gamble”: a qualitative analysis of how people who engage in gaming and gambling understand a link between these two behaviours. J Gambl Stud.

[91] Calado F, Alexandre J, Griffiths MD (2014). Mom, dad it’s only a game! perceived gambling and gaming behaviors among adolescents and young adults: an exploratory study. Int J Ment Health Addict.

[92] Rockloff M, Russell AMT, Greer N, Lole L, Hing N, Browne M (2021). Young people who purchase loot boxes are more likely to have gambling problems: an online survey of adolescents and young adults living in NSW Australia. J Behav Addict.

[93] Duffy L (2021). Gen bet: a plain english summary of research into gambling and young people. Victorian Responsible Gambling Foundation.

[94] Greer N, Rockloff M, Hing N, Browne M, King DL (2023). Skin gambling contributes to gambling problems and harm after controlling for other forms of traditional gambling. J Gambl Stud.

[95] Sidloski B, Brooks GA, Zhang K, Clark L (2022). Exploring the association between loot boxes and problem gambling: are video gamers referring to loot boxes when they complete gambling screening tools?. Addict Behav.

[96] Greer N (2023). Experiences with esports betting and skin gambling: exposure, access, motivations and impacts [Thesis]. https://acquire.cqu.edu.au/articles/thesis/Experiences_with_esports_betting_and_skin_gambling_Exposure_access_motivations_and_impacts/23897322/1.

[97] Drummond A, Hall LC, Sauer JD (2022). Surprisingly high prevalence rates of severe psychological distress among consumers who purchase loot boxes in video games. Sci Rep.

[98] Cole JD (2023). Understanding school counselors’ perceptions of esports and igaming as a career choice [Doctoral dissertation]. https://ir.library.oregonstate.edu/concern/graduate_thesis_or_dissertations/j9602803m.

[99] Declerck P, Feci N (2022). Mapping and analysis of the current regulatory framework on gambling(- like) elements in video games: a report in the framework of the ‘gam(e)(a)ble’ research project. http://hdl.handle.net/1854/LU-8768539.

[100] Laato S Is simulating casino environments in video games worse than gambling with loot boxes? The case of the removed pokémon game corner. http://ceur-ws.org/Vol-2737/SP_1.pdf.

[101] Xiao LY (2022). Which implementations of loot boxes constitute gambling? A UK legal perspective on the potential harms of random reward mechanisms. Int J Ment Health Addict.

[102] Kolandai-Matchett K, Wenden Abbott M (2022). Gaming-gambling convergence: trends, emerging risks, and legislative responses. Int J Ment Health Addict.

[103] Whitson J, French M (2021). Productive play: the shift from responsible consumption to responsible production. J Consumer Cult.

[104] Schwiddessen S, Karius P (2018). Watch your loot boxes! – Recent developments and legal assessment in selected key jurisdictions from a gambling law perspective. IELR.

[105] McCaffrey M (2020). A cautious approach to public policy and loot box regulation. Addict Behav.

[106] Amadieu T, Chrétien-Ichikawa S, Pawlik K (2022). Creative Industries and Digital Transformation in China.

[107] Delfabbro P, King DL (2020). Gaming-gambling convergence: evaluating evidence for the ‘gateway’ hypothesis. Int Gambl Stud.

[108] Garea SS, Drummond A, Sauer JD, Hall LC, Williams MN (2021). Meta-analysis of the relationship between problem gambling, excessive gaming and loot box spending. Int Gambl Stud.

[109] Biegun J, Edgerton JD, Roberts LW (2021). Measuring problem online video gaming and its association with problem gambling and suspected motivational, mental health, and behavioral risk factors in a sample of university students. Games Cult.

[110] Delfabbro P, King DL, Lambos C, Puglies S (2009). Is video-game playing a risk factor for pathological gambling in Australian adolescents?. J Gambl Stud.

[111] Castrén S, Järvinen-Tassopoulos J, Raitasalo K (2021). Money used in gaming is associated with problem gambling: results of the ESPAD 2019 Finland. J Behav Addict.

[112] Rodda S (2020). A rapid review and research gap analysis: a 2020 update. Commissioned by the NSW Responsible Gambling Fund. GambleAware.

[113] Stevens MW, Dorstyn D, Delfabbro PH, King DL (2021). Global prevalence of gaming disorder: a systematic review and meta-analysis. Aust N Z J Psychiatry.

[114] De Leon A (2024). Cutting losses: Southeast Asia’s crackdown on online gambling. The Diplomat.

[115] Rattaphong S (2022). Governance and strategies of gambling business in Southeast Asia. CBSR.

[116] Wardle H, Degenhardt L, Marionneau V (2024). The Lancet Public Health Commission on gambling. Lancet Public Health.

[117] Burleigh TL, Griffiths MD, Sumich A (2022). Co-occurrence of gaming disorder and other potentially addictive behaviours between Australia, New Zealand, and the United Kingdom. Int J Environ Res Public Health.

[118] King DL, Delfabbro PH (2016). Adolescents’ perceptions of parental influences on commercial and simulated gambling activities. Int Gambl Stud.

[119] Lia DAZ, Natswa SL Buy-now-pay-later (BNPL): generation Z’s dilemma on impulsive buying and overconsumption intention.

[120] Brand M, Wegmann E, Stark R (2019). The Interaction of Person-Affect-Cognition-Execution (I-PACE) model for addictive behaviors: update, generalization to addictive behaviors beyond internet-use disorders, and specification of the process character of addictive behaviors. Neurosci Biobehav Rev.

[121] Håkansson A, Widinghoff C (2019). Television gambling advertisements: extent and content of gambling advertisements with a focus on potential high-risk commercial messages. Addict Behav Rep.

[122] Bae S, Han DH, Jung J, Nam KC, Renshaw PF (2017). Comparison of brain connectivity between internet gambling disorder and Internet gaming disorder: a preliminary study. J Behav Addict.

[123] Close J, Spicer SG, Nicklin LL, Lloyd J, Whalley B, Lloyd H (2021). Gambling and gaming in the United Kingdom during the COVID-19 lockdown. COVID.

[124] Zendle D, Cairns P (2018). Video game loot boxes are linked to problem gambling: results of a large-scale survey. PLoS One.

[125] Zendle D, Cairns P (2019). Loot boxes are again linked to problem gambling: results of a replication study. PLoS One.

[126] Drummond A, Sauer JD, Ferguson CJ, Hall LC (2020). The relationship between problem gambling, excessive gaming, psychological distress and spending on loot boxes in Aotearoa New Zealand, Australia, and the United States—a cross-national survey. PLoS One.

[127] Liu K (2019). A global analysis into loot boxes: is it “virtually” gambling? Washington. Int Law J.

[128] Guerrin CGJ, Doorduin J, Sommer IE, de Vries EFJ (2021). The dual hit hypothesis of schizophrenia: evidence from animal models. Neurosci Biobehav Rev.

[129] Bayer TA, Falkai P, Maier W (1999). Genetic and non-genetic vulnerability factors in schizophrenia: the basis of the “two hit hypothesis”. J Psychiatr Res.

[130] Shi J, Colder Carras M, Potenza MN, Turner NE (2020). A perspective on age restrictions and other harm reduction approaches targeting youth online gambling, considering convergences of gambling and videogaming. Front Psychiatry.

[131] Haskell JV (2015). More than just skin(s) in the game: how one digital video game item is being used for unregulated gambling purposes online. J High Technol Law.

[132] McCaffrey M (2019). The macro problem of microtransactions: the self-regulatory challenges of video game loot boxes. Bus Horiz.

[133] Sanders J, Williams R (2019). The relationship between video gaming, gambling, and problematic levels of video gaming and gambling. J Gambl Stud.

