# Supplementary material for: Scrutinizing the Gateway Relationship Between Gaming and Gambling Disorder: Scoping Review With a Focus on the Southeast Asian Region
Source: JMIR Serious Games. 2025 Jan 15;13:e59740. doi: 10.2196/59740 (PMC11753718; doi:10.2196/59740)
Supplement: Multimedia Appendix 1 [file games-v13-e59740-s001.docx]

**PubMed:**

1. game* [tw]
2. gaming [tw]
3. esport$ [tw]
4. electronic sport$ [tw]
5. exp Games, Experimental/ [mesh]
6. exp Video Games/ [mesh]
7. exp Games, Recreational/ [mesh]
8. exp Exergaming/ [mesh]
9. exp Technology Addiction/ [mesh]
10. exp Internet Addiction Disorder/ [mesh]
11. or/1-10
12. gambl* [tw]
13. bet$ [tw]
14. betting$ [tw]
15. exp Gambling/ [mesh]
16. or/12-15
17. 11 AND 16
18. exp Addiction Medicine/ [mesh]
19. exp Behavior, Addictive/ [mesh]
20. 18 OR 19
21. 17 AND 20

#Notes: [tw: text words that include all words and numbers], [exp: explode for MeSH terms], MeSH terms are denoted by capital letters, slash marks, and square brackets at the end

**ScienceDirect:** (game* OR gaming OR esport$ OR electronic sport$ OR games, experimental OR video games OR Games, Recreational OR exergaming OR Technology, Addiction OR internet addiction disorder) AND (gambl* OR bet$ OR betting$ OR Gambling) AND (Addiction Medicine or Behavior, Addictive)

**Google Scholar:** (game OR gaming OR games or esports or "electronic sports") AND (gamble OR gambling OR bet OR betting) AND (addiction OR addictive OR addiction OR behavior addiction)

**Proquest:** noft(game* OR gaming OR esport OR electronic sport OR experimental game OR video games OR exergaming) AND noft(gambl* OR bet OR betting) AND noft(Addiction Medicine OR Behavior Addiction OR Addictive)

#Notes: [noft: searches the full bibliographic record]

**Garuda:** (game OR gaming OR games or esports or "electronic sports") AND (gamble OR gambling OR bet OR betting) AND (addiction OR addictive OR addiction OR behavior addiction)
