# Supplementary material for: Scrutinizing the Gateway Relationship Between Gaming and Gambling Disorder: Scoping Review With a Focus on the Southeast Asian Region
Source: JMIR Serious Games. 2025 Jan 15;13:e59740. doi: 10.2196/59740 (PMC11753718; doi:10.2196/59740)
Supplement: Multimedia Appendix 2 [file games-v13-e59740-s002.docx]

| **No** | **First Author** | **Country** | **Year** | **Type of Publication  (Empirical, Review, Viewpoint/Commentary)** | **Design (Commentary, Editorial, Review, Case Reports, Cross-sectional Survey, Cohort, Case-control, Quasi-experimental, RCT, if others specify)** | **Longitudinal** | **Relevant Objectives of the Study** | **N** | **Age  (mean, median or range)** | **Sex  (% of female)** | **Main types of Gaming-Gambling Relationship (Converging Mechanics, Correlation/Comorbidity, Gateway Effect)** | **Relevant Notable Findings** |
| --- | --- | --- | --- | --- | --- | --- | --- | --- | --- | --- | --- | --- |
| 1 | Iain, R | UK | 1989 | Review | Narrative Review | No | Discuss correlation between gaming, gambling, and risk taking | - | - | - | Correlation/Comorbidity | Playing video games induce arousal or excitement. The study proposed a developmental model of pathological interaction with technologies to video game addiction to gambling involvement as the person ages, thus machine gambling addiction stems from early problematic man-machine relationship. |
| 2 | Griffiths, MD | UK | 1991 | Review | Literature Review | No | Review the literature on the concerns between UK fruit machines and US video game machines | - | - | - | Converging mechanics | Both machines are amusement machines, they are commonly located within the same vicinity (arcades) thus sharing not only structural commonalities but also physical elements. Further, it has been proposed a model of development between video game addiction to gambling addiction throughout the lifespan from childhood to adulthood. |
| 3 | Fisher, S | UK | 1995 | Review | Narrative Review | No | Explore slot machines and pathological gambling as well as the overlap between slot machines and video game playing | - | - | - | Converging mechanics | Both machines have been suggested to induce dependency. Some shared characteristics include a software loop governing predictable response, rapidity in play span, winnings induce flood of aural and visual cues, provide digital score for 'correct behavior', and opportunity to attract peer attention or approval. |
| 4 | Ladouceur, R | Canada | 1995 | Empirical | Cross-sectional | No | To study the gambling behavior in the involvement of non-gambling video game arcades | 122 | 19.2±5.5 | 17.20% | Correlation/Comorbidity | Within the sample, 83.6% had gambled once in their life and 29.5% gambled regularly once a week. Among the arcade video game players, 10.7% were identified as probable pathological gamblers and 14.8% screened as problem gamblers. Frequency of attending these video game arcades correlate to SOGS score (r: 0.40, p<0.0001). |
| 5 | Gupta, R | Canada | 1996 | Empirical | Cross-sectional | No | Evaluate the link between video game and gambling in children | 104 | 9-14 | 50.96% | Correlation/Comorbidity | High frequency children visit arcade game 8.5 times per month vs low frequency 0.9 times per month. Children perceived games to be skill-driven (51%) and only 20% considered in to require a lot of luck. 70% of children reported having gambled in the past and 55% once a week or more. High frequency gamers tended to gamble larger sums of money than low frequency gamers. The pattern persisted for males but not females. Though there was no correlation of winnings to gaming frequency. |
| 6 | Greenberg, JL | USA | 1999 | Empirical | Cross-sectional | No | Observe the tendency for "multiply addicted" and overlapping addictions | 129 | 20.5 | 50.40% | Correlation/Comorbidity | High correlation between video game and gambling (r: 0.72). |
| 7 | Griffiths, MD | UK | 2000 | Review | Literature Review | No | Review the evidence on gambling, videogame playing and Internet use among adolescents | - | - | - | Correlation/Comorbidity | Adolescents face risks for not only gambling but also videogame playing, in which the latter can also be noted as non-monetary forms of gambling. The technologically-advanced forms of gambling were more appealing to adolescents. |
| 8 | Haninger, K | USA | 2004 | Empirical | Cross-sectional | No | Verify and categorize the type of content for Teen-rated video games | 396 | - | - | Converging mechanics | None of the games received descriptors of containing gambling contents, upon experimentation 1% (n: 1) involved some gambling scenes. |
| 9 | Johansson, A | Norway | 2004 | Empirical | Cross-sectional | No | Assess the gambling-like problems among adolescents playing non-monetary games | 2,050 | 12-18 | 32.87% | Correlation/Comorbidity | Among the study sample, 28.1% correspond to loss of control criteria and 16.4% to preoccupation |
| 10 | Messerlian, C | Canada | 2004 | Review | Literature Review | No | Investigate the risk of gambling from rising gambling technology and the internet among youths | - | - | - | Converging mechanics | The gambling industry adapted advancements from the gaming industry to its products. Online gambling is faceted with multitude of colors, fast-paced qualities similar to video games. |
| 11 | Wood, RTA | UK | 2004 | Empirical | Cross-sectional | No | Explore the structural characteristics of video games | 382 | 21.1±4.75 | 36.60% | Converging mechanics | In terms of game dynamics, 75.2% felt that elements of surprise in a game is important, which was the second highest. Finding new things such as secret doors, hidden levels, were also appealing to most (65.3%) as well as findings bonuses (52.2%). Some these structural characteristics portray the allure of chance-probabilistic nature embedded in games, replicating that of gambling activities. |
| 12 | Wood, RTA | Canada | 2004 | Empirical | Cross-sectional | No | Examine the relationship between video game, gambling and other related factors | 996 | 10-17 | 55.10% | Correlation/Comorbidity | Regular gamblers were more likely to be regular gamers. Hours playing video games were correlated to problem gambling severity, r: 0.18, p<0.001. High frequency male gamers were more likely to be problem gamblers (11.0% vs. 6.9%), similarly in female, 5.9% vs 1.0%. |
| 13 | Griffiths, MD | UK | 2005 | Viewpoint/Commentary | Correspondence | No | Comparing the similarities between playing video game arcades and slot machines | - | - | - | Converging mechanics | Observations of commonalities had been proposed along with respective addictive potential, which require further empirical corroboration. |
| 14 | Parker, JDA | Canada | 2008 | Empirical | Cross-sectional | No | Assess the link of gaming and internet abuse and emotional intelligence to problem gambling in adolescence | 667 | 16.2±1.45 | 62.70% | Correlation/Comorbidity | Weak correlation, r: 0.30 p< 0.05, between SOGS-RA and PVGS (problem videogaming). |
| 15 | Griffiths, MD | UK | 2008 | Viewpoint/Commentary | Viewpoint | No | Discuss the overlap between gambling and technologies | - | - | - | Converging mechanics | Video game technology has been utilized within gambling products to attract the public. On the other hand, gaming platforms have begun offering monetary rewards based on game performance. The convergence widen into games in social media and even interactive TV programs. Research is required for advantages and disadvantages of this convergence. |
| 16 | Delfabbro, P | Australia | 2009 | Empirical | Cross-sectional | No | To scrutinize if video game playing is a risk factor to pathological gambling | 2,669 | 14.6 | 49.50% | Gateway effect | Regression analysis found very small beta values, indicating minute causal possibility. Alternatively, the group of daily video game player had higher DSM-IV-J scores than the rest of the sample (Mean 0.41 vs 0.16, p<0.001, d: 0.25) |
| 17 | Johnson, TE | USA | 2009 | Empirical | Quasi-experimental | Yes | Analyze if simulated game could conditioned future gambling-like preferences | 7 | 7-10 | 28.6% | Gateway effect | 6 out of 7 participants were conditioned to prefer the color of die that was previously exposed to as contextual cues |
| 18 | King, DL | Australia | 2010 | Review | Literature Review | No | The various forms of gambling in the virtual world and new gambling-like mechanics among modern games | - | - | - | Converging mechanics | Young people are at greater risk of problematic gambling and new forms of gambling in digital media have increased this risk. Additionally, exposure has been more ubiquitous with the addition of gambling features surrounding (in- and out of) video games, with little legal protections. |
| 19 | Griffiths, MD | UK | 2010 | Viewpoint/Commentary | Viewpoint | No | Review the potential risks and harms posed by social games | - | - | - | Converging mechanics | Social games have evolved to adopt exploitative practices, termed as freemium. The games are free to install, but certain aspect require monetary payments and could boosts gameplay. Some have noted the potency of social games to be a gateway to potentially problematic activities such as gambling. |
| 20 | Griffiths, MD | UK | 2010 | Viewpoint/Commentary | Viewpoint | No | To comment on the penetration of gaming with gambling-features in social networking sites | - | - | - | Converging mechanics | The presence of gambling activities on non-gambling platform may normalize the image of gambling for the public, particularly as it is packaged within gaming modules, for example Farmville. The game has some gambling-like features that may not be apparent, so much so that some cases have reported children spending money on the game without parental knowledge. |
| 21 | Griffiths, MD | UK | 2011 | Viewpoint/Commentary | Commentary | No | Outline the legal and psychosocial considerations on gaming and gambling convergence | - | - | - | Correlation/Comorbidity | Opportunities of gaming and gambling convergence is vast and will continue to grow. A phenomena occur in which video game servers pay players for every kill in a first-person shooting game, which is comparable to some online gambling forms. Masking activities as skill-based maybe a legal loophole to avoid implications. Other social games have also allowed for virtual payments accessible to children without parental knowledge. It is debatable whether activities blending these skills/chance elements are considered as gambling, but should not be legally isolated. Legal protection should be available for vulnerable groups, such as age checking and monetary security measures. |
| 22 | King, DL | Australia | 2012 | Empirical | Cross-sectional | No | Exploring the association of video game playing to cognitive error and risky behaviors of gambling | 50 | 21.8±2.8 | 4% | Correlation/Comorbidity | Video game players were moderately involved with gambling activities, such as card gambling, lotteries, and scratch tickets (3-5 times per year). In a trial gambling task, players demonstrated accurate estimation of past and future wins according to the set probability. They also attribute wins due to chance. Study concluded no association of video game playing to either higher gambling involvement or cognitive error. |
| 23 | Walther, B | Germany | 2012 | Empirical | Cross-sectional | No | Examine the co-occurrence between addictions (including gaming and gambling) | 2,553 | 16.7±3.0 | 49.30% | Correlation/Comorbidity | Correlation between addiction of gaming and gambling was small, r: 0.12 |
| 24 | Parker, JDA | Australia | 2012 | Review | Literature Review | No | To overview what defines social gambling and the its variety of forms | - | - | - | Converging mechanics | Some of the social gambling forms that have converge with video games are virtual world gambling, SNS (Freemium) Gambling, and app gambling. About 3-5% were estimate to purchase in-game items, of which 56% will make a second purchase, and 25% three or more purchases. |
| 25 | King, DL | Australia | 2012 | Review | Literature Review | No | Assess the rating and regulation for games with gambling elements | - | - | - | Correlation/Comorbidity | The evidence demonstrated that warnings and ratings had been inconsistent or not adequate. Of 102 games with gambling elements that had been released, 69 were rated parental guidance and 33 as generally suitable for all ages. |
| 26 | Griffiths, MD | UK | 2012 | Viewpoint/Commentary | Viewpoint | No | Discuss the psychological aspect of games within social networking sites | - | - | - | Converging mechanics | Some gambling companies have leveraged gambling mechanics in their video game designs in order to increase monetary spending of the players. One of the key ways this is achieved is through operant conditioning and the use of random reinforcements. Social gamers are also becoming similar to slot gamblers, in the sense that they know the money they are putting into the game is going to be lost but they are willing to pay to play. |
| 27 | Critselis, E | Cyprus | 2013 | Empirical | Cross-sectional | No | Investigate association of gambling to other internet addictive behavior | 805 | 13-18 | 30.70% | Correlation/Comorbidity | Among frequent internet gamblers (n: 109), 92.7% reported using internet for game-playing and 6.4% fulfilled criteria for addictive behavior. |
| 28 | Jiménez-Murcia, S | Spain | 2014 | Empirical | Cross-sectional | No | Explore the rate of video game use and addiction among gambling disorder patients | 193 | 42.4±13.4 | 13.50% | Correlation/Comorbidity | Among gambling disorder patients, 62.7% played video games and 15.0% were classified as video game addicted. Majority of those with video game addiction (69.0%) gamble using slot machines. Patients with VGA had higher SCL-90-R scores with moderate effect size. In pathway analysis, VGA score was not predictive of SOGS score. |
| 29 | Calado, F | Portugal | 2014 | Empirical | Qualitative | No | Scrutinize the association between gambling and gaming behaviors along with its consequences | 37 | 13-26 | - | Correlation/Comorbidity | Adolescents view gaming as a potentially addictive behavior, with certain genre being able to be played for gambling activities (e.g., MMORPG). While young adults viewed gaming as more of a platform for social interaction and improving skills. Adolescents also emphasized that underage gambling accessibility and opportunity was growing due to its social prestige and financial rewards, although gambling was perceived requiring maturity. |
| 30 | Gainsbury, SM | Australia | 2014 | Review | Literature Review | No | Review the consequences of gaming and gambling convergence in the virtual world | - | - | - | Correlation/Comorbidity | The review proposed algorithm and taxonomy to differentiate specific online gambling-related services. Such that practice game and stand-alone video games were ultimately differentiated whether it was provided by a gambling operator. While social casino game and social game were distinct on whether gambling theme is central to the gameplay. |
| 31 | King, DL | Australia | 2014 | Empirical | Cross-sectional | No | Assess the rate of adolescent involvement with social media and digital gambling | 1,287 | 14.9±1.5 | 50.40% | Correlation/Comorbidity | 314 adolescents had played gambling activities in video games (e.g., Runescape), of those 46.1% were identified as problem gamblers. The most common monetary gambling utilized by simulated gamblers were scratch card (15.3%), racing bets (10.4%), and card games (9.4%), with significant relationship and moderate effect size (eta: 0.27). Overall, pathological gambling prevalence was 1.0%. |
| 32 | Griffiths, MD | UK | 2014 | Viewpoint/Commentary | Viewpoint | No | Provide a summary of recent findings on social games and gambling on networking sites | - | - | - | Converging mechanics | Increased genres of social networking games. Some games include exploitative practices, such as freemium and foot-in-the-door technique. This reinforces addiction through impulse buying. In-game virtual goods and accessories are psychological masterstroke. In gambling games, you can win back your initial investment back, thus such prospect adding layers of complexity to the model. |
| 33 | Griffiths, MD | UK | 2014 | Review | Book | No | Collate evidence on the convergence of gaming and gambling activities | - | - | - | Correlation/Comorbidity | Convergence between gaming and gambling had started out with its co-location as amusement machines in arcades. Modern convergence has been the embedding of gambling features in games. Studies have also shown the correlation of gaming and gambling addiction symptoms. |
| 34 | Castrén, S | Finland | 2015 | Empirical | Cross-sectional | No | Examine the association of gambing and gaming participation among at-risk/problem gambling adolescents | 988 | 13.4±0.4 | 46.80% | Correlation/Comorbidity | Video game playing weekly or more twice the odds than less than once a week for at-risk/problem gambling (OR: 2.42 [1.3-4.5]) |
| 35 | Delfabbro, P | Australia | 2015 | Review | Literature Review | No | Scrutinize the gambling cognitive errors theories and approaches applicability to video gaming | - | - | - | Correlation/Comorbidity | There is lack of evidence that erroneous beliefs central to gambling disorder translate well to gaming disorder. The paper proposed four cognitive categories that should be focused on in gaming disorder. |
| 36 | Forrest, CJ | Australia | 2015 | Empirical | Cross-sectional | No | To identify overlapping video gaming and gambling habits among video game players | 485 | 25.8 | 16% | Correlation/Comorbidity | Among the video game players, the mean gambling occasion was 1.74 in the past 3 months. Most preferred gambling for money than credits/points. GAS score was not correlated to gambling for money but small correlation to gambling for credits/points (r: 0.09). On regression, GAS score predicted gambling frequency (B: 0.03, IRR: 1.03 [1.01-1.05]) |
| 37 | Griffiths, MD | UK | 2015 | Viewpoint/Commentary | Viewpoint | No | To determine the mechanics of gambling-like features in a game is similar to gambling or gaming, using example of Runescape | - | - | - | Converging mechanics | Certain gambling-like activities within Runescape meet the legal criteria for gambling within the Gambling Act in the UK as well as psychological definitions set out in the literature. Multiple forms of mini-games within the larger game also hand out items with real-world value, thus might constitute as activities requiring purview of the gambling commission as well. |
| 38 | Haskell, JV | USA | 2015 | Viewpoint/Commentary | Editorial | No | Discuss the emergence of virtual skins as 'chips' of unregulated gambling | - | - | - | Converging mechanics | Skins are first created for cosmetic purposes in gameplay. These have evolved to become "de facto currency" similar to casino chips for placing bets in marketplaces (esports betting, roulette games, virtual coin flips, etc.), many of which are unregulated. Identification or age verification has been very lacking in these websites and game providers thus credibility of crack-downs by major game industry is also questioned. The gaming industry could limit API access, also by recognizing esports as legitimate sports, certain regulations would be applicable to regulate esports betting. |
| 39 | Tárrega, S | Spain | 2015 | Empirical | Quasi-experimental | Yes | Determine the efficacy of serious games to alleviate gambling symptoms | 16 | 34.08±6.02 | 0% | Correlation/Comorbidity | Three modules of serious games, face of cronos, treasures of the sea, and sign of the magupta, are delivered. SOGS score decreased by 3.75 points (p: 0.047, d: 0.81) and BIS by 10.9 points (p: 0.028, d:0.86). |
| 40 | King, DL | Australia | 2015 | Review | Literature Review | No | To differentiate gaming and gambling definitions in addiction research through its overlap on several activities | - | - | - | Converging mechanics | The usage of category-based nomenclature such as gambling-life game is too obscure to comprehend the gaming/gambling overlap. 3 types of definitions for gambling-like contents are proposed considering the interactivity, monetization, wagering mechanics, outcome determination, outcome measurement, fidelity, context, centrality, and advertisement. |
| 41 | Brand, M | Germany | 2016 | Review | Literature Review | No | Investigate a theoretical model uniting the internet addictive disorders | - | - | - | Correlation/Comorbidity | Though there is "first-choice" use may correlate to the resulting disorder, internet use disorders is highly overlapping thus may present as mixed disorder (internet-gaming and internet-gambling disorder) |
| 42 | Griffiths, MD | UK | 2016 | Review | Literature Review | No | Examine the gambling-type games accesible by children and adolescents | - | - | - | Converging mechanics | There are few evidence on impact of gambling-type activities within games to draw conclusions. However, stricter age verification is recommended, particularly for games accessible to children and adolescents |
| 43 | Thege, BK | Canada | 2016 | Empirical | Cross-sectional | No | Explore the distinct subpopulation having multiple addictions (including substance and non-substance) | 2,728 | 45.1±13.5 | 62.20% | Correlation/Comorbidity | Problematic video gaming and problematic gambling co-occur in 4 latent clusters, particularly cluster VII had 100.0% problematic video gaming participants with co-occurring 12.3% problematic gambling. |
| 44 | Larche, CJ | Canada | 2016 | Empirical | Cross-sectional | No | Explore the near-miss trigger for arousal, urge and frustation in "Candy Crush" game similar to gambling | 60 | 21±1.4 | 84.20% | Correlation/Comorbidity | Near-miss sessions were reported to induce higher scores of frustration compared to losses and wins, however, the reported urge and post-reinforcement responses were only significant compared to losses and not winning sessions. |
| 45 | King, DL | Australia | 2016 | Review | Literature Review | No | Discuss models of simulated gambling (e.g., gambling-like video games) early exposure leading to risks and benefits | - | - | - | Correlation/Comorbidity | Develops two models, first the containment model as protection and catalyst as risks for simulated gambling. The cognitive variable within the risk model included misinterpretation of profitability and perceiving gambling as gaming. Simulated gambling among video games were becoming ubiquitous through enhanced structural features, e.g., broadcasted through streaming platforms and embedded promotional contents. |
| 46 | Kinnunen, J | Finland | 2016 | Empirical | Qualitative | No | Examine the role and nuances of money among free-to-play (F2P) gamers and gamblers | 16 | 32.38±8.62 | 18.75% | Converging mechanics | Among F2P players, play money is not separated from money for other basic necessities. In F2P, there is rarely separate accounts to set aside an amount of money specifically for playing. Thus the low framing of money management among F2P players. |
| 47 | McBride, J | Canada | 2016 | Empirical | Cross-sectional | No | Examine the similarities between gambling and gaming particularly on their psychological and behavioral domains | 1,229 | 18.69±1.41 | 56.55% | Correlation/Comorbidity | On the overall sample only 0.48% were identified as probable problematic gamblers, while 2.8% screened with gaming addiction. 604 (49.1%) reported involvement in gambling and gaming in the past year. Addicted gamers had the highest gambling participation (62.9%) than non-gamer (30.8%) or regular gamers (54.2%). Of the addicted gamers that gamble, 11.4% were problematic gamblers. |
| 48 | King, DL | Australia | 2016 | Empirical | Cross-sectional | No | Examine the effect of parents on adolescents gambling involvement across all platforms | 824 | 14.1±1.5 | 50.97% | Correlation/Comorbidity | Video games with gambling was included as gambling with virtual money or credits. Of the participants, 1.2% reported having been involved in the past year. About 21.6% received invitation but only 1.1% reported having sent out invitations. 7.9% of video game gamblers were unsupervised, 1.4% alternatingly, and 0.1% had continuous supervision. Parental modelling had low association. |
| 49 | Lopez-Gonzales, H | Spain | 2016 | Review | Narrative Review | No | Review the evidence for online sports betting including esports | - | - | - | Correlation/Comorbidity | Esports is the results of sportification of video games, which has garnered huge mass and traction with competitions handing out millions of dollars in prizemoney. The humongous interest has led gambling operators to chip in and design a betting platform for esports. The companies pitch bettors against each other and take commission from winning bets, contrary to house versus gambler design of traditional sports gambling. |
| 50 | Bae, S | South Korea | 2017 | Empirical | Cross-sectional | No | Analyze IGD and ibGD neurobiological mechanism in which instruments were administered for all groups | 15 | 25.7±5.5 | 0% | Correlation/Comorbidity | Among IGD group, participants scored 5.5±2.4 on the PG-YBOCS (Yale-Brown Obsessive Compulsive Scale for Pathological Gambling) which was similar to healthy controls but lower than pathological gambling. |
| 51 | Estévez, A | Spain | 2017 | Empirical | Cross-sectional | No | Explore relationship of substance and non-substance addiction through emotiona regulation | 472 | 15.6±1.3 | 51.60% | Correlation/Comorbidity | Video game addiction and gambling disorder had significant correlation at r: 0.32 |
| 52 | James, RJE | UK | 2017 | Viewpoint/Commentary | Commentary | No | Discuss development of gaming and gambling disorder, adding on the overlap between the two | - | - | - | Correlation/Comorbidity | Convergence of gaming and gambling can be in both directions. Scrutiny on gamblification of games have increased although many focused on the simulated element of gambling in-game through the use of virtual currencies. |
| 53 | Sanders, J | Canada | 2017 | Empirical | Cross-sectional | No | Identify the co-morbidity of multiple addictive behaviors | 3,942 | 43.6 | 50.50% | Correlation/Comorbidity | From the sample, 1.2% were identified as concurrent problems gamblers and problem video gamers (10.6% as only problem gamblers and 3.9% as only problem video gamers). The group with concurrent PG/PVG had higher antisocial characteristics, impulsivity, and substance misuse risk |
| 54 | Gainsbury, SM | Australia | 2017 | Empirical | Cross-sectional | No | To compare the harm experienced by esports bettors versus conventional sports bettors | 501 | 45.5±14.8 | 32.20% | Converging mechanics | Sports bettors respondents have been gambling longer than esports bettors (20.92 years vs 13.53 years, p< 0.001). However, esports bettors demonstrated higher degree of problematic gambling behavior (M= 9.64) than traditional sports bettors (M= 3.44, p< 0.001, d= 1.06). Esports bettors also significantly gambled more daily than sports bettors. |
| 55 | Gainsbury, SM | Australia | 2017 | Empirical | Cross-sectional | No | To outline the profile of Australian esports gamblers | 160 | 45.5±14.8 | 32.20% | Converging mechanics | Esports bettors were younger than sports bettors (p< 0.001), more highly educated (p< 0.001), and more like to be actively employed (p< 0.001). Interestingly, esports gamblers were also less likely to have children than sports bettors (p< 0.05). In contrast, onset age of gambling for esports bettors were higher (p< 0.001). Esports bettors were also more likely to utilize offshore websites, citing conveniences such as lack of identity regulation, ease of account creation, and no betting limits. |
| 56 | Teichert, T | Germany | 2017 | Empirical | Cross-sectional | No | To investigate similarities between 16 gaming and 9 gambling products | 3000 triads | - | - | Converging mechanics | Consumers perceived several similarities between gaming and gambling. Strategy games such as collectible card games are perceived similarly to other strategy based gambling product such as poker. Casino gambling is also linked to management games. Virtual slot machines had moderate pairwise association to action games. This similarities are based on secondary dimensions such as luck vs skill and experience type. |
| 57 | Abarbanel, B | USA | 2018 | Empirical | Qualitative | No | Assess qualitatively the perception on match fixing in esports betting | 1321 | - | - | Converging mechanics | Match fixing as a form of cheating was not detrimental according to respondents, compared to other forms of cheating. Esports consumer also appear to be less aware about the complexity of match fixing. Some also voiced their perception that cheating to lose could be a strategic note. Others who noticed match fixing in esports argue that it did impact the esports integrity. |
| 58 | Armstrong, T | Australia | 2018 | Review | Literature Review | No | Investigate effect of simulated gambling exposure towards real-money gambling behavior | - | - | - | Gateway effect | Simulated gambling includes those played in standalone console, online or mobile games; which might correlate to the high exposure of simulated gambling games among youth. These gambling-like features also promote gambling biases among youth and desensitize them to monetary losses. |
| 59 | Chen, JH | Hong Kong | 2018 | Empirical | Cross-sectional | No | Scrutinize the comorbid prevalence of gambling disoder with internet gaming disorder, depression and anxiety | 1000 | 40±15.3 | 56% | Correlation/Comorbidity | The study identified 187 participants were frequent gamblers with 10.2% (n: 19) of them as probable gamblers. Among probable gamblers, 4 fulfilled DSM-5 criteria for IGD and the calculated odds for IGD among probable gamblers were OR: 12.53. |
| 60 | De Pasquale, C | Italy | 2018 | Empirical | Cross-sectional | No | Assess the prevalence of internet gaming disorder among Italian university students, with details on their game types | 221 | 21.6±1.4 | 57.90% | Correlation/Comorbidity | The study reported high incidence of high-risk IGD 84.6% among the sample, with 23% of the sample reported game types of online gambling. |
| 61 | Hayer, T | Germany | 2018 | Empirical | Longitudinal | Yes | Examine whether simulated internet gambling (intersection between gambling and computer gaming) increased risk for real-world monetary gambling | 1,178 | 13.6±1.4 | 52.50% | Gateway effect | Recorded simulated internet gambling activities included forms of microtransactions in-game, e.g., purchasing virtual currency, cash payments to increase odds of winning, or to reduce imposed delay between plays. Transition to real-world monetary gambling were significantly predicted through participation of simulated gambling on social networking sites (OR: 2.42 [1.25-4.69]) and exposure to gambling advertisements (OR: 3.53 [1.04-12.00]). Simulated gambling through video games, computer games or mobile games did not demonstrate statistical significance. |
| 62 | Håkansson, A | Sweden | 2018 | Empirical | Cross-sectional | No | Investigate the prevalence of lifetime problem gambling and problem gaming among national-level athletes | 352 | 23.7±3.18 | 60% | Correlation/Comorbidity | The study found 23 and 8 participants had problem gambling and problem gaming respectively. Of those, 3 had concurrent problem gambling and problem gaming. The calculated odds for concurrent disorders was OR: 9.72 (2.17-43.60). |
| 63 | King, DL | Australia | 2018 | Viewpoint/Commentary | Editorial | No | Outline the growing schemes of monetization of video games which is predatory and association to internet gaming disorder | - | - | - | Converging mechanics | Monetization in video games is growing in the forms of microtransactions that incentivize the players. These features may propagate spending and involvement through concepts e.g., entrapment and sunk-cost bias. The game and developers may also utilize information asymmetry to 'personalize' purchasing offers toward specific players thus increasing the odds of those players buying into the schemes. |
| 64 | Rémond, JJ | France | 2018 | Empirical | Cross-sectional | No | Investigate the link between gambling practice and screen practice (video game, Internet, mobile screen) | 432 | 21.9±5.51 | 50.90% | Correlation/Comorbidity | Among the 8 participants identified as pathological gamblers, 5 participants also had pathological video game addiction test scores. There were also significant group mean differences on VAT scores which was highest among pathological gamblers and at-risk gamblers but lower for non-problematic gamblers (F: 15.49, p<0.001) |
| 65 | Kircaburun, K | Turkey | 2018 | Empirical | Cross-sectional | No | Assess the relationship of dark traits with specific online activities including gaming, gambling, shopping, scoial media and sex | 772 | 20.7±2.30 | 64% | Correlation/Comorbidity | Gaming use and gambling had significant correlation r: 0.26. Both gaming and gambling was predicted by Machiavellianism. |
| 66 | Bányai, F | Hungary | 2018 | Review | Literature Review | No | Review evidence regarding electronic sports from its psychological aspects, including the convergence of gaming and gambling | - | - | - | Correlation/Comorbidity | Gaming has been considered as non-financial form of gambling and its evolution to esports extends this relationship. Future esports research should also look into the detrimental aspect of involvement, akin to professional gambling, particularly as esports involved players making a financial living out of it which potentially shifts the motivation than casual gaming. |
| 67 | Molde, H | Norway | 2018 | Empirical | Longitudinal | Yes | Investigate the transition between problem gaming and problem gambling | 4,601 | 48±15.1 | 52.80% | Gateway effect | The Gaming Addiction Scale at Time 1 had significant correlation to Canadian Problem Gambling Index at Time 1 and 2 (both r: 0.25), while the GAS at Time 2 had significant correlation to CPGI T1 and T2 at r: 0.19. In a cross-lagged model, the GAS at T1 predicted CPGI at T2 (B: 0.15, p<0.001), but CPGI T1 did not predict GAS at T2 (B: 0.05, p>0.05). |
| 68 | Peter, SC | USA | 2018 | Empirical | Cross-sectional | No | Evaluate the level of stigma attributed to traditional gambling, esports gambling, and internet gaming | 504 | 37.2±11.10 | 50.60% | Correlation/Comorbidity | Within the study sample, 24% were identified as pathological gambler, 24% as pathological gamer, and 25% as pathological gambling gamer. eSports gambling was seen as dangerous as casino gambling, but more dangerous than Internet gaming. eSports gambler was significantly more feared than financial crisis. |
| 69 | Sanders, J | Canada | 2018 | Empirical | Cross-sectional | No | Study the cross-addictrion involvement between gambling and video gaming | 3,942 | 43.6±15.9 | 50.50% | Correlation/Comorbidity | Among the participants, 10.6% were assessed as problem gamblers while another 3.9% as problem gamers and 1.2% as problematic gamblers and gamers. Among gamers (n: 203), 78.5% had gamble in the past year and 24.1% were problem gamblers. While among gamblers (n: 466), 70.7% had also play video game in the past year and 10.5% were problem video game players. Correlation between overall gambling and gaming frequency was small (tau-b = .11, p < .05). Higher impulsivity (UPPS) were consistently found to discriminate between general problematic behaviour vs. non-problematic use, problem gambling vs. problem gaming, and concurrent problem gambling/gaming vs. single problematic use (last group had OR: 1.1) |
| 70 | Wu, Y | China | 2018 | Empirical | Cross-sectional | No | Investigate the gambling cognitive distortions among IGD and healthy control | 44 | 21.4±1.3 and 22.0±1.7 | 0% | Correlation/Comorbidity | IGD group did not display higher PGSI score than healthy control. However, IGD group had higher gambling expectancy (d: 0.66), illusion of control (d: 0.81), and predictive control (d: 1.10) than healthy control. IGD group also demonstrated overall higher motivational ratings than control for near-miss before but not near-miss after. |
| 71 | Drummond, A | Australia | 2018 | Viewpoint/Commentary | Commentary | No | Expand that loot boxes is psychologically similar to gambling | - | - | - | Converging mechanics | Out of 22 game titles observed, at least 10 contained loot boxes feature. A further 6 of those ten provided ability to cash out winnings, and all were rated appropriate for 13 years old or younger. Even in the strictest definition of gambling adopted, at least 23% of the scrutinized games fulfilled the criteria. |
| 72 | Griffiths, MD | UK | 2018 | Viewpoint/Commentary | Commentary | No | Comment on the current state of gaming and gambling convergence understanding | - | - | - | Correlation/Comorbidity | The scientific discourse on potential gaming and gambling discourse goes back to more than two decades ago. Loot box has become ubiquitous in the modern video games industry and it is akin to gambling. Now game addiction also constitute not only loss of time but also loss of money through the excessive purchase of in-game items. There is also a growing trend of crypto-addiction which is essentially gambling addiction. |
| 73 | Schwiddessen, S | USA | 2018 | Review | Literature Review | No | Examine loot boxes from the gambling legal perspective | - | - | - | Correlation/Comorbidity | Gambling law in each jurisdiction is different and might not be applicable to cover loot boxes. Several international jurisdictions have launched investigations to regulate loot boxes, e.g., Japan, China, Singapore, South Korea, Germany, Australia. While Poland for example have already covered loot boxes as applicable gambling. There is still doubt pertaining secondary market of these in-game items and loot boxes. |
| 74 | Zendle, D | UK | 2018 | Empirical | Cross-sectional | No | Scrutinize the risk of loot box involvement to problem gaming and problem gambling | 7,422 | 18-45+ | 9% | Correlation/Comorbidity | Overall, 104 (1.40%) gamers were identified as problem gamblers. Severity gradient was observed from problem gambling to non-problem gamblers on loot box spending and on other microtransactions. With small to medium effect size (η2 = 0.054) for the former and small effect size for the latter (η2 = 0.009). |
| 75 | King, DL | Australia | 2018 | Review | Literature Review | No | To define gaming, gambling and the areas they are overlapping along with the risks posed to children and adolescents | - | - | - | Correlation/Comorbidity | Youth at the ages of 13-17 have been exposed to gambling-like activities and promotions through video games, social media, etc. Some are also participating into unregulated third party gambling sites. Some gambling products have been sophisticatedly designed to normalize the activity, particularly around esports, games, and streaming platforms. |
| 76 | Schluter, MG | Canada | 2018 | Empirical | Cross-sectional | No | Develop and examine cross-addiction instrument | 6,000 | 18-55+ | 53.80% | Correlation/Comorbidity | The instrument's video gaming addiction domain had moderate correlation to PGSI, r:0.50 and similarly the instrument's gambling addiction domain also had a moderate correlation to GAIA (Gaming Addiction Inventory for Adults), r: 0.54. |
| 77 | Macey, J | Finland | 2018 | Empirical | Cross-sectional | No | Scrutinize the link between various gambling types and video game consumption, particularly esports | 613 | <14-50+ | 6.20% | Gateway effect | Of the whole sample, 4.2% were identified as problem gamblers and 6.5% with video game addiction. Video game habits (B:0.116) and esports viewing (B: 0.167) had small association to video-gambling habits , but online gambling habit had moderate effect (B: 0.602). Video game gambling involvement had small predictive value to PGSI (B: 0.347). |
| 78 | Griffiths, MD | UK | 2018 | Viewpoint/Commentary | Viewpoint | No | Review whether loot boxes constitute gambling | - | - | - | Converging mechanics | The purchase of loot boxes at its essence is a virtual game of chance. This is sometimes performed in multiple steps to negate the feeling of gambling, players have to buy a certain in-game currency using real money, after which they have to convert that currency to another 'soft currency' in order to purchase a key (or similar items) to open the loot boxes. Some regulatory bodies in Europe maintain that loot boxes is not a form of gambling since you always get something out of it. In contrast, Asian countries viewed loot boxes as a form of gambling or encouraged gambling-like behavior thus incorporate it into their regulations. |
| 79 | Vadlin, S | Sweden | 2018 | Empirical | Cohort | Yes | Assess whether problematic gaming correlates prospectively with problematic gambling in adolescents | 1,576 | 13 or 15 | 58% | Gateway effect | Among the sample, 23.6% were problematic gamers in wave 1, 19.7% remained problematic gamers in wave 2 and 1.7% were identified as problematic gamblers in wave 2. Problematic gaming (GAIT) score wave 1 was predictive of problematic gambling wave 2 (eta-squared: 0.007, p: 0.001, OR: 1.886[1.125-3.161]), age (eta-squared: 0.007, p:0.001) and male sex (eta-squared: 0.008, p: 0.001, OR: 0.201[0.055-0.733]) were also predictive however male sex seemed to be protective. |
| 80 | Office of the eSafety Commissioner | Australia | 2018 | Empirical | Cross-sectional | No | Provide overview of the state of gamers and gambling-like features exposure to youths | 3,017 | 8-17 | - | Correlation/Comorbidity | 80% had played in the past year and 50% had played esports games in the past year. 34% ever made microtransactions inside games in the past year (adolescents 38% vs. children 32% and boys 51% vs. girls 34%). 15% estimated watched esports and 8% participated in a tournament. |
| 81 | Brooks, GA | Canada | 2019 | Empirical | Cross-sectional | No | Investigate the relationship of gaming and in-game microtransactions to gambling and gambling-related cognitive errors | 257 | 28.3 | 32.70% | Correlation/Comorbidity | Across the sample, 60.3% reported ever purchasing loot boxes and 79.3% mentioned that loot box felt akin to gambling while 86.2% agreed that loot box is a type of gambling. Problem gambling severity index dan internet gaming disorder test scores were moderately correlated r: 0.426. Among the sample of video gamers, around <10% fulfilled scores for problem gambling. |
| 82 | Drummond, A | New Zealand | 2019 | Viewpoint/Commentary | Letters to Editor | No | Discuss the overlap between lootboxes and traditional gambling features, with possible regulatory control over the lootboxes | - | - | - | Converging mechanics | Defined loot boxes as virtual containers in-game that contained digital items at random. These probabilistic trait of gambling being accessible to children is worrisome and that loot boxes lead to higher spending among those with symptoms of problem gambling. |
| 83 | Li, W | USA | 2019 | Empirical | Cross-sectional | No | Examine the link between loot box purchase to problem gaming and problem gambling | 618 | 27±8.9 | 36.30% | Gateway effect | Around 44.0% of the gamers reported having purchased loot boxes in the past 12-months. Those who purchased loot boxes had much higher IGD score (11.81 vs. 5.61). Similarly, they also reported much higher PGSI scores, 11.49 vs. 2.67. In a path analysis, loot box purchases predicted problem gaming (B: 0.22) and problem gambling (B: 0.14) directly and indirectly though increased online gaming and gambling frequency and number of sessions. |
| 84 | Marmet, S | Switzerland | 2019 | Empirical | Cross-sectional | No | Assess the role mental health problems play in instigating behavioral addictions and substance addictions | 5,516 | 25.5±1.26 | 0% | Correlation/Comorbidity | Among the study sample, 7.0% were identified as gaming addiction and 1.4% as gambling addiction. Of those with gaming addiction, 26.3% also had gambling addiction. Conversely, from those with gambling addiction 5.2% were also assessed with gaming addiction. |
| 85 | Wardle, H | UK | 2019 | Empirical | Cross-sectional | No | Evaluate the consequences of skin gambling involvement among children and adolescents | 2,881 | 11-16 | 50.90% | Gateway effect | From the sample, 7% had participated in skin gambling in the past month, in contrast to 16% for any other forms of gambling. Skin betting is more popular among boys and older age group (15-16 vs. 11-12). Among children skin betting, 39% also gambled on at least one other form, conversely among other gamblers, only 16% participated in skin gambling. Those who did online gambling had higher odds than other forms for skin betting. 23% of those who did skin betting and other gambling forms were identified as problematic gamblers, compared to 8% of those who only did other forms of gambling. However, skin betting alone did not predict problematic gambling but online gambling did (OR: 8.4, 1.9-37.4) |
| 86 | Derevensky, JL | Canada | 2019 | Viewpoint/Commentary | Viewpoint | No | Discuss the convergence of gaming and gambling, and how each industry should protect the consumers | - | - | - | Correlation/Comorbidity | Gaming and gambling activities have converge in their structure and design. The gaming industry have been targeting games for younger and younger population (as low as 3+ year olds) and gambling-themed games will normalize the behavior upon this early exposure. The gambling industry have been working with academics to promote responsible gambling. More studies to understand similar risk in gambling-themed games are required so the gaming industry could follow suit in designing consumer protection codes. |
| 87 | Gainsbury, SM | Australia | 2019 | Viewpoint/Commentary | Viewpoint | No | Comment on the research, regulation and reaction toward gaming-gambling convergence | - | - | - | Correlation/Comorbidity | More research is needed to delineate the association between gaming and gambling disorders, particularly the directionality through longitudinal observations. Aside from the studied risks, potential benefits from this convergence should be explored to fully understand the relationship and guide future policies. Regulation on new gambling technologies remains warranted, particularly towards vulnerable consumers. |
| 88 | Greer, N | Australia | 2019 | Review | Literature Review | No | Provide overview of the evolution of esports betting and skin gambling | - | - | - | Converging mechanics | Some early evidence have demonstrated the increased risk through esports betting towards monetary gambling involvement. However, studies on skin gambling have been lacking behind. Both are very accessible to minors. |
| 89 | Karlsson, J | Sweden | 2019 | Empirical | Cross-sectional | No | Analyze the relationship between internet use, gaming, and gambling problems | 1,593 | 15-60+ | 48% | Correlation/Comorbidity | Overall, 9.8% scores as problem gamblers with significant interaction to gender and GAS (Gaming Addiction scale) scores (OR: 1.14, 1.09-1.19) |
| 90 | Liu, K | USA | 2019 | Viewpoint/Commentary | Commentary | No | Discuss whether loot box is essentially gambling | - | - | - | Converging mechanics | Loot box have been exploited through the ease of online payments, operant conditioning, and targeted advertisements. Countries have been reacting differently, e.g., outright banning, regulating, launching investigations, or dismissing loot box as simply a game feature. Other approaches could be taken such as ensuring a zero-sum game and eliminating pay-to-win environment, enhancing parental control for online payments, and disclosing odds of winning. |
| 91 | Macey, J | Finland | 2019 | Empirical | Cross-sectional | No | Examine the association of esports to gambling activities | 582 | <14-50+ | 5.50% | Correlation/Comorbidity | Within the sample, 51% had gambled and spectate esports in the past year. The number increased to 67.18% including loot boxes purchases. 4.5% fulfilled criteria for problematic gambling and 18% for moderate risk. Video game related gambling-activities and loot box purchases demonstrate significant associations to problematic gambling. |
| 92 | Zendle, D | UK | 2019 | Viewpoint/Commentary | Correspondence | No | Comment the convergence of games and gambling through loot boxes | - | - | - | Converging mechanics | Loot boxes, owing to its chances-based nature, make it similar to gambling. The deleterious link between loot box spending and gambling severity seems to be even stronger in adolescents. |
| 93 | Scholten, OJ | UK | 2019 | Empirical | Cross-sectional | No | Assess characteristics and gameplay of cryptogames running on Ethereum cryptocurrency | 9 | - | - | Converging mechanics | The paper combined psychological and legal definition of gambling, particularly on the ability to 'cash-out'. Of the 9 cryptogames, all fulfilled the gambling criteria, that the exchange of virtual items have unknown probability, the outcome is due to chance, requires the exchange of money or items of financial value, losses can be avoided by not participating, and ability to cash out to real-world money. By design these cryptogames focus on generation and ownership of virtual goods, however, the chance-based element is central. There is also a feature for decentralized exchanges. |
| 94 | Zendle, D | UK | 2019 | Empirical | Cross-sectional | No | Determine the association of loot box and gambling behavior in adolescents | 1,155 | 16-18 | 9% | Correlation/Comorbidity | 40.5% had purchased loot box in the past month and a majority (80.6%) bought their first loot box after 1 month of playing the game. Gamers who bought loot box had 4x the mean PGSI score (4.318) than non-buyers (1.719). Conversely, problem gamblers also spent the most on loot boxes than other risk-groups gamblers. Most gamers buy loot boxes for gameplay advantages (21.9%) rather than pursuing profit (0.9%). |
| 95 | Zendle, D | UK | 2019 | Empirical | Cross-sectional | No | Replicate the association of loot box and gambling behavior in adults | 1,172 | 18-40+ | 31% | Correlation/Comorbidity | Problem, moderate, and low risk gamblers had significantly higher loot box spending than non-problem gamblers with moderate effect sizes (d: 0.429-0.568). This pattern remains when analyzed for in-game objects apart from loot boxes. Correlation of PGSI scores to loot box was r: 0.238 and r: 0.164 for PGSI scores to other microtransactions. |
| 96 | McCaffrey, M | UK | 2019 | Viewpoint/Commentary | Viewpoint | No | Provide contrary view to the definition of loot boxes as gambling | - | - | - | Correlation/Comorbidity | Firms have begun to rethink and redesign their game development and marketing strategies. Including self-regulation on loot boxes and other microtransactions. Address the stakeholder's complaints on current designs and the complex relationship to regulatory bodies. Additionally, increase direct support and involvement to customers communities such as in the gambling industry. |
| 97 | Gambling Commission | UK | 2019 | Empirical | Cross-sectional | No | Provide nationally representative data on gambling-like games participations | 2,943 | 11-16 | 45% | Correlation/Comorbidity | 52% had heard of in-game items, and 44% had paid money for microtransactions, e.g., opening loot boxes, for those in-game items. Mostly utilized money from pocket money. 7% have bet money on online monetary gambling platforms. 1.7% is classified as problem gambling. 29% played gambling-like game before gambling (35% for boys vs. 17% for girls). |
| 98 | King, DL | Australia | 2019 | Review | Book | No | Summarize information on internet gaming disorder, also provide difference and similarities to gambling | - | - | - | Correlation/Comorbidity | Gaming has been involved with monetized schemes. Within loot boxes is similar to gambling slot machines, as they require no skill and provide random outcomes at a determined proportion that most times are not declared. Some games promote this purchase to progress or gain items in-game. |
| 99 | Abarbanel, B | USA | 2019 | Empirical | Cross-sectional | No | To investigate the relationship between esports spectatorship to esports betting, whether it is mediated by gambling product advertising, and perception of appropriateness of such advertisement | 1049 | 37.0±13.47 | 30.30% | Gateway effect | Among esports bettors, there was significantly higher rates of having seen gambling ads (21.8% vs 5.0%, p< 0.001). Additionally, they also perceived these ads as being more appropriate compared to non-bettors (35.2% vs 16.0%, p< 0.001). This pattern was replicated among esports spectators, having seen ads in the past year (10.1% vs 4.1%, p<0.001) and perceiving them as appropriate (25.0 vs 11.6%, p< 0.001). |
| 100 | Sweeney, K | USA | 2019 | Empirical | Cross-sectional | No | To provide specific difference of esports betting to traditional sports betting | 3651 matches | - | - | Converging mechanics | Esports bettors had favorite-longshot bias especially when involving real money market. Some of the esports gambling markets are also prone to arbitrage from informed bettors. |
| 101 | Dagaev, D | Russia | 2020 | Empirical | Cross-sectional | No | To analyze pattern of esports bettors to a famous first-person shooting game | 3129 matches | - | - | Converging mechanics | There is a reverse favorite-longshot bias among esports bettors. Bettors prefer to bet on the underdogs. This is counterproductive as the analysis documented inefficiency of market persisting across time, comparing bets made during or outside of matches. There was no difference across geographical sites. |
| 102 | Putra, MTP | Indonesia | 2020 | Viewpoint/Commentary | Narrative Review | No | This research explores the monetization system of lootboxes in video games, emphasizing their gambling-like traits and the consequences of these traits in relation to Indonesian law. | - | - | - | Gateway effect | Research data indicates that the loot box monetization system contains inherent gambling elements and negative effects, yet it remains legally ambiguous. These gambling aspects are considered illegal under Indonesian laws and regulations. The government needs to undertake legal research on the loot box monetization system and establish regulations governing such practices in Indonesia. |
| 103 | Rodda, S | Australia | 2020 | Review | Systematic Review | No | To provide gap analysis on current evidence | - | - | - | Converging mechanics | Emerging technologies blur the boundaries between gambling and gaming, such as loot boxes. Gamblers on social casino games online are also more likely to migrate to other online gambling forms and are at higher risk to problematic gambling. Other convergence of gambling and gaming include skin betting. Some evidence of esports spectatorship also increased risk to transitioning to gambling and experiencing gambling harm. |
| 104 | Russell, AMT | Australia | 2020 | Empirical | Cross-sectional | No | To investigate the effect of gambling-like features exposure to gambling-related harm | 2004 | 23.7±3.6 | 63.00% | Converging mechanics | Early exposure to loot box, esports, skin betting, and watching gambling advertisement all correlated positively to gambling-related harm. Onset of exposure after age 18 demonstrate higher risk for risky gambling compared to exposure before 18 years old. |
| 105 | Beranuy, M | Spain | 2020 | Empirical | Cross-sectional | No | Validate the IGDS9-F and its concurrent validity to OGD-Q | 535 | 18.4±2.13 | 21.50% | Correlation/Comorbidity | The IGDS9-F scores were moderately correlated to OGD-Q scores, r: 0.44, p<0.001 |
| 106 | Drummond, A | New Zealand | 2020 | Empirical | Cross-sectional | No | Investigate the links of loot boxes purchase to problem gaming and problem gambling | 1,288 | 40±15.4 | 63.40% | Correlation/Comorbidity | Loot box purchase was significantly correlated to PGSI (r: 0.328), risky loot box index (r: 0.392), positive mood (r: 0.163), negative mood (0.140), and psychological distress (r: 0.138). Problematic gamblers and moderate gamblers spent the most on loot boxes in hierarchical manner, while low-risk and no-risk gamblers were similar in spending amount. RLI was also moderately correlated to PGSI (r: 0.411) and IGD (r: 0.600). PGSI predicted loot box purchase (B: 0.267) more than IGD or IGD/PGSI concurrently. |
| 107 | Ford, M | Sweden | 2020 | Empirical | Cross-sectional | No | Assess the relationship between problem gambling to other behavioral addictions and mental health conditions | 2,038 | 18-(>)60 | 54.70% | Correlation/Comorbidity | Within the sample, 116 participants were identified as problem gamblers and they scored much higher than non problem gambler in Gaming Addiction Scale (median 10 vs 7). GAS also predicted problem gambling (OR: 1.13, 1.07-1.19) |
| 108 | González-Cabrera, J | Spain | 2020 | Empirical | Cross-sectional | No | Develop OGD-Q for adolescents and assess the relationship to IGD-20 | 2,691 | 14.3±1.55 | 52.90% | Correlation/Comorbidity | The correlation between OGD-Q and IGD-20 among adolescents was significant and small, r: 0.19, p<0.001 |
| 109 | Kim, HS | Canada | 2020 | Empirical | Cross-sectional | No | Investigate the relationship across 10 addiction disorders and their indicators | 3,503 | >18 | 57.10% | Correlation/Comorbidity | Among the sample, 236 (6.7%) reported problems with video games in the past year and 254 (7.3%) with gambling. 807 had two addiction problems and 787 had three or more concurrent problems. |
| 110 | King, A | USA | 2020 | Empirical | Cross-sectional | No | Examine the overlap of video game and gambling disorders | 300 | 22.8±2.00 | 51% | Gateway effect | Internet gaming disorder severity and gambling disorder severity correlated significantly, r: 0.375. All items on RLI correlated to IGD, and also to GD except for item 2 (increased amount of spending on loot boxes). Problematic gaming (OR: 6.45, 1.69-24.54) and problematic gambler (OR: 5.62, 1.51-20.98) were also risk factors of each other. 55.4% of gamers had purchase in-game currency in the past month. Relationship of IGD to GD were partially mediated by microtransactions (b: 0.13, 0.06-0.22, p<0.01) |
| 111 | King, DL | Australia | 2020 | Viewpoint/Commentary | Literature Review | No | Review the basic attributes of gaming and gambling, how gaming is becoming monetized, and how these affect the behavioral outcomes | - | - | - | Correlation/Comorbidity | Most studies have focused on the microtransaction elements within video games, comparing it to gambling and how it increases participation to gambling. Some evidence have suggested the increased risk to problematic gambling and problematic gaming. |
| 112 | King, DL | Australia | 2020 | Empirical | Cross-sectional | No | Assess the gaming motivations toward microtransactions spending in-game | 428 | 23.5±7.3 | 6.50% | Correlation/Comorbidity | In-game spending was predicted by availability of payment methods, frequency of closest friends making purchases, older age, and number of hours weekly playing the game. Gaming disorder was associated with higher impulsivity, validation seeking, and weekly gaming hours. |
| 113 | Kotyuk, E | Hungary | 2020 | Empirical | Cross-sectional | No | Scrutinize the concurrent presence of substance and non-substance addictions | 3,003 | 21±2.8 | 57.40% | Correlation/Comorbidity | Among the sample, 50 was assessed with problematic gambling and 12.2% had problematic online gaming. Conversely, 113 were screened as problematic online gaming and 0.5% had concurrent problematic gambling. |
| 114 | McCaffrey, M | UK | 2020 | Viewpoint/Commentary | Commentary | No | Discuss on regulatory approaches toward loot box and how recent research might be too hasty | - | - | - | Converging mechanics | Correlation does not show much on the indicated harm, in this case from loot box and subsequence problem gambling among vulnerable groups. The gaming industry was already making changes and more transparency toward these gambling-like features, e.g., loot boxes. Outright banning of loot boxes is posited to turn it into the black markets thus disabling monitoring of such practices and consequent behavior on players. |
| 115 | Richard, J | Canada | 2020 | Review | Systematic Review | No | Investigate the links between conduct problem, depression symptoms, problem gambling and problem gaming | - | - | - | Correlation/Comorbidity | Only 1 study fulfilled the criteria for concurrent PG/PVG, in which the primary study only identified 1.3% of sample as PG/PVG. Overall, conduct problems and depressive symptoms seem to be associated however more conclusive data is required. |
| 116 | Shi, J | Canada | 2020 | Review | Rapid Review | No | Examine the outcome of measures restricting gambling, e.g., age restrictions and harm reductions, particularly in the context of gaming-gambling convergence | - | - | - | Correlation/Comorbidity | Some governmental and regulatory bodies have considered loot boxes as gambling, with skin gambling and other random-chance features in game also similar to gambling. Recent findings suggest that age-restrictions and harm reduction measures are warranted for video games containing gambling or gambling-like features. |
| 117 | Zendle, D | UK | 2020 | Empirical | Cross-sectional | No | Assess the proportion of top grossing video games containing loot boxes and their accesibility to children | 250 | - | - | Converging mechanics | Out of the top 100 games in Google Playstore, 58.0% incorporated loot boxes into their mechanics. Similarly 59.0% titles were found in Apple Appstore. While among the top 50 titles in Steam platform, 36.0% contained loot box feature. Of the games containing loot box, 93.1% in Google, 94.9% in Apple and 38.8% in Steam were accessible to children (rated safe for 12+ year olds). |
| 118 | Prati, AM | USA | 2020 | Viewpoint/Commentary | Commentary | No | Comment on the legal urgency due to loot boxes and gambling overlap | - | - | - | Converging mechanics | In light of the loot boxes exposure to children induce an urgency for regulatory action, as existing regulations contain lapses. Enough disclosure is required for consumers, particularly parents, are able to make informed decisions. |
| 119 | Drummond, A | New Zealand | 2020 | Viewpoint/Commentary | Commentary | No | Comment on the possibility to regulate loot box as a gambling mode | - | - | - | Converging mechanics | Technological innovation has always been a step ahead of regulation and governance, thus there are many weakly regulated activities in the virtual world. Though the science has begun to shed light on the value of digital items and microtransactions, legal approaches have lagged behind. |
| 120 | Gainsbury, SM | Australia | 2020 | Empirical | Cross-sectional | No | Measure the relationship between EGM, SGM, mobile game, and video games | 184 | 34.02±9.29 | 32.10% | Correlation/Comorbidity | Participants with involvement in SGM had higher frequency of mobile and video games. Mobile games also predicted SGM frequency (B: 1.404, p<0.05) |
| 121 | Kalkan, B | Turkey | 2020 | Empirical | Cross-sectional | No | Examine the rates and link between internet use, online gaming, and online gambling | 222 | 25.04±7.07 | 60.80% | Correlation/Comorbidity | There was small correlation between Online GSAS (Gambling Symptom Assessment Scale) and POGQ (Problematic Online Gaming Questionnaire), r: 0.25, p<0.01 |
| 122 | Macey, J | Finland | 2020 | Empirical | Cross-sectional | No | Assess gambling-related cognitive instruments on gamer population | Set 1: 442 Set 2: 391 Set 3: 335 | Set1: 33.5% <30 Set 2: 85.7% <30 Set 3: 36.7% <30 | Set 1: 36.4% Set 2: 6.6% Set 3: 42.2% | Correlation/Comorbidity | GRCS did not appear to be psychometrically sound to assess gambling gamer's cognitive properties although model had good fit. Thirteen items from GRCS were added with 5 new items for a new GamCog instrument. Cognitive biases seem to be retained among gamers, however perception of skill and luck were not suitable for gamers. |
| 123 | Serada, A | Finland | 2020 | Viewpoint/Commentary | Case Study | No | Discuss whether "CryptoKitties" can be constituted as a form of gambling | - | - | - | Converging mechanics | CryptoKitties is similar to other pet breeding games, with an obscure goal that players can determine. However, it requires the input of certain amount of cryptocurrencies to receive random qualities of virtual kitties. The kitties can be bred with chance-based system, traded on market, and cashed out. Expectedly, rare breeds of kitties are priced higher. Overall, it is based on a probabilistic system similar to gambling but require certain skills of breeding (strategizing) and trading (pertaining cryptocurrency ledger). |
| 124 | Wardle, H | UK | 2020 | Empirical | Cross-sectional | No | Explore the characteristics of esprots bettors | 3,549 | 16-24 | 49% | Correlation/Comorbidity | Esports bettor had higher odds of paying money for loot boxes (OR: 10.45), skin gambling on external websites (OR: 2.57) and private skin gambling (OR: 5.89). Esports bettors are also more involved in gambling, 50% gambled in more than 5 gambling types and 53% had PGSI score above 8. |
| 125 | Zendle, D | UK | 2020 | Empirical | Cross-sectional | No | Examine the seven mechanisms of loot boxes involving gambling-like elements | 1,200 | 18-40+ | 37.10% | Converging mechanics | Of the participating gamers, 17.7% were classified as problem gamblers according to revised PGSI scoring scheme. Gamers who engaged in monetary loot box had a mean score (5.407) double that of those who used non-monetary loot box (2.190). Ability to cash out via in-game marketplace, ability to cash out via external websites, both in-game and external, and being able to trade items with other players strengthen the relationship between loot boxes and problem gambling (B: 0.049-0.090). The amount of money made from selling loot boxes weakened the link (B: -0.005). Paying to win, presence of near misses, use of virtual currency, and exclusivity of items also strengthened the relationship. |
| 126 | DeCamp, W | USA | 2020 | Empirical | Cross-sectional | No | Scrutinize whether loot boxes share similar characeristics of risk or protective factors with gambling | 13,042 | 13-14 and 16-17 | 29-50% | Correlation/Comorbidity | Nearly 30% of 8th grade and 25% of 11th grade students had at least bet in a video once in their life (either through loot boxes or downloadable content [DLC]). The female sex posed lower odds for betting in game of chance, game of skills, loot box and DLC purchases. History of neighborhood bullying was significant to both games of chance and loot box betting. While depression/anxiety symptom was linked to DLC purchases |
| 127 | Delfabbro, P | Australia | 2020 | Review | Literature Review | No | To collate evidence on the gateway hypothesis of gaming to gambling | - | - | - | Gateway effect | Only small correlation between video game and gambling have been published so far. The attraction might go both ways between gaming and gambling. Nevertheless, loot box and problem gambling seem to be positively associated. Overall, evidence was scarce and need further longitudinal research. |
| 128 | Biegun, J | Canada | 2020 | Empirical | Cross-sectional | No | Scrutinize an instrumen to measure problem video game playing and implement to measure reverse gateway hypothesis from problem gambling to gaming | 651 | 18-24+ | 52.50% | Gateway effect | Problem gambling only had weak correlation to problem video gaming, r: 0.105. Problem gambling did not predict problem video game playing. |
| 129 | Labrador, FJ | Spain | 2020 | Empirical | Cross-sectional | No | Analyze the effect of gambling advertisement of video game and gambling habits | 2,887 | 15.35±2.69 | 42.54% | Correlation/Comorbidity | Overall, participants demonstrate a weak effect of advertising of games of chance (correlation scores <0.3). There is significant difference on GoC advertising effect between those who played video games and those who did not, except of the items of whether GoC advertisement increase gambling interest. |
| 130 | Kristiansen, S | Denmark | 2020 | Empirical | Cross-sectional | No | Explore the engagement of youths to loot boxes and its association to problem gambling | 1,137 | 12-16+ | 50.60% | Gateway effect | 56.1% of participants were involved with loot boxes with 42.5% purchased loot boxes in the past year, but only 10.6% ever sold items obtained from loot boxes. Purchasing loot box or selling loot box were associated with higher gambling frequency and problematic gambling. |
| 131 | Kim, HS | Australia | 2020 | Viewpoint/Commentary | Editorial | No | Collate published papers on gaming-gambling convergence | - | - | - | Correlation/Comorbidity | The studies shared a theme of how new technologies enable novel gambling opportunities which might be more detrimental to vulnerable populations. |
| 132 | Li, W | USA | 2020 | Empirical | Cross-sectional | No | Validate problematic social casino game scale and correlated to video game playing and monetary gambling | 436 | 30.59±10.76 | 42.66% | Correlation/Comorbidity | 386 of the sample had played video games, of which 23.06% also played social casino game daily, 15.28% more than once a week, and 15.80% at least once a week. IGD and PGSI scores were also significantly higher among those with higher SCG frequency. |
| 133 | Gainsbury, SM | Australia | 2020 | Empirical | Qualitative | No | Explore the relationship between skill gaming machines and EGM | 21 | 18-39 | 52.38% | Correlation/Comorbidity | Participants understood difference of SGM and EGM, however they did not comprehend the correlation of the reel-gambling component to the video gameplay in SGM. Participants expressed preference to SGM as they perceive they had more control and develop mastery owing to the partly skill-based nature to obtain money. They were concerned with the steep learning curve on SGM as instructions were minimal. |
| 134 | Brosowski, T | Germany | 2020 | Empirical | Cohort | Yes | To understand the mediation of the varying simulated gambling including through video games, apps, social networks, and demo games | 1,178 | 13.6±1.40 | 52.50% | Gateway effect | The rate of simulated gambling participation through video games is the highest at 40%, social apps 19.3%, social networks 14.33% and demo games 9.76%. SG in video games affect problematic gambling significantly through depth of gambling and cognitive fallacies. SG in video game and social networking sites also impacted gambling onset through advertisement exposure. |
| 135 | Mills, DJ | USA | 2020 | Empirical | Cross-sectional | No | Examine the relationship of problematic gambling and problematic gaming symptoms | 1,621 | 20.55±2.70 | 54.50% | Correlation/Comorbidity | Among the sample, 6.1% were assessed as problematic gambling and 22.7% as problematic gaming. Only 2.2% had concurrent PG/PVG and this group had higher risk for comorbid substance use. |
| 136 | Abarbanel, B | USA | 2020 | Empirical | Cross-sectional | No | Assess the gambling-elements in game streaming platforms through content analysis | 414 | - | - | Converging mechanics | Of the 414 extensions analyzed, three groups were relevant to gambling and gaming, which are extensions for games, loyalty and recognition, and viewer engagement. At least one of three gambling legal definition (consideration, prize, chance) is present in each of the group. Of these five themes emerged, presence of traditional gambling elements (e.g., terminologies), prediction actions, disguised legal components of gambling, raffle giveaways labelled as charitable gambling scenarios, and marketing practice normally discouraged in current gambling advertising standards. |
| 137 | Macey, J | Finland | 2020 | Empirical | Cross-sectional | No | Analyze the relationship between digital video games, gambling, and social casino games | 946 | 43 | 49.50% | Correlation/Comorbidity | Social casino games frequency was influenced by digital game play duration (tau: 0.196), including with chance-based or skill-based gambling elements. In-game purchases (tau: 0.188), offline gambling, and online gambling also demonstrated significant association to SCG frequency. |
| 138 | Zendle, D | UK | 2020 | Empirical | Cross-sectional | No | Explore the rates of loot box, esports betting, real-money video gaming, token wagering, and social casino among adults along with their harms | 1,081 | 18-58+ | 50.79% | Correlation/Comorbidity | A total of 18.5% of the participants had engaged one of the gambling-like video game practices at least once in the past year. The most common being loot boxes (7.8%), then watching loot box opening recording (6.6%) or live (4.8%), and watching online gambling recorded (4.2%) or live (4.1%). The correlation of any gambling-like video game practice was significant albeit small, rho 0.23. All practices influenced problem gambling. |
| 139 | von Meduna, M | Germany | 2020 | Empirical | Cross-sectional | No | Analyze the impact of gambling-like products in games | 1,508 | 36.7 | 44.70% | Correlation/Comorbidity | Sampled from P2W users online, of which 586 (38.9%) purchased loot boxes. Of these, 45.9% were screened as problem gamblers and 68.9% as problem gamers, this is significantly higher than non-loot box purchaser. Age negative correlated to ever purchasing loot box, inconclusive for sex. Income is not linked to loot box purchase. P2W gaming harm is linked to ever purchasing loot box but not frequency. Problem gambling did not predict loot box habits. |
| 140 | Zanescu, A | Canada | 2020 | Review | Narrative Review | No | Discuss the gamblification of DOTA 2 | - | - | - | Converging mechanics | Battle Pass is a brimming gambling system that is aimed to elicit specific participation from players. Players are geared to habituated consumption through hybrid gambling systems promoting labor/play system. |
| 141 | Laato, S | Finland | 2020 | Viewpoint/Commentary | Case Study | No | Examine the effect of certain game mechanics to promote gambling | - | - | - | Converging mechanics | The game corners in Pokémon games have been identified as simulating real world gambling by regulatory bodies and the game is rate 12+, however a re-release of the game in a different title containing the same mechanics received a rating of 7+. The difference might lay on the need to not only display gambling images but provide activities that simulate gambling. Laws have always fallen behind due to the rapid changes in the gaming industry. Parental guidance is important for children and adolescent. Nevertheless, the industry also has a duty to maintain consumer's health including psychological health. |
| 142 | Greer, N | Australia | 2020 | Empirical | Report | No | Delineate the demographics and correlations in problematic gaming and gambling activities | 744 | 32.02 | 32.80% | Gateway effect | 41.5% of samples were involved in esports skin betting, esports cash betting, and skin gambling. The most common demographics are young and male. They are also engaged in traditional gambling forms. Though most respondents had younger age of traditional gambling participation than video game gambling. 25.4% gambled in video games before traditional gaming, 47.8% gambled traditionally before video games, and 26.7% gambled in both platforms at the same time. |
| 143 | Rockloff, M | Australia | 2020 | Empirical | Cross-sectional | No | To elucidate the risk of loot box and problem gambling among youths | 1954 | 12-24 | 59.90% | Converging mechanics | Loot box involvement in the past 1-2 years pose more risk than those with earlier experiences. However, there is no link between onset age to gambling risk. Association of loot box and gambling problems were seen in both adolescents and young adults populations. |
| 144 | Castrén, S | Finland | 2021 | Empirical | Cross-sectional | No | Scrutinize if monetary elements in video games are predictors for gambling and other risk factors moderating the association | 4,595 | 15-16 | 50.50% | Gateway effect | Problematic gaming alone did not significantly predict involvement in gambling (in the past year), but monetary spending in-game did predict problematic gambling (OR: 1.48, 1.09-1.99). In the study sample, 13.1% were identified as problematic gamers, 10.9% spent money in-game, and 37.7% had an episode of gambling in the past year. |
| 145 | Hall, LC | New Zealand | 2021 | Empirical | Cross-sectional | No | Explore the influence of self-isolation/quarantine on excessive gaming, gambling and loot box purchases | 1,144 | 31.4±10.5 | 43.60% | Correlation/Comorbidity | Problem gamblers spend the most on loot box, while moderate and low risk gamblers spend similarly, and non-problem gambler spend the least (F[3,1,131]: 34.679, p<0.001). Stronger correlation was found among those who isolated with higher scores of PGSI (r: 0.366), than those who did not isolate (r: 0.209). |
| 146 | Hing, N | Australia | 2021 | Empirical | Cross-sectional | No | Investigate the relationship between skin gambling, monetary gambling, and problem gambling | 1,669 | 14.6±1.66 and 14.8±1.64 | 37.60% | Correlation/Comorbidity | Among the advertisement sample, 55.3% had ever done skin gambling in the past month and 22.4% among qualtrics sample. IGD predicted skin gambling significantly in both group. Similarly, nearly all forms of monetary gambling predicted past-month skin gambling. Subsequently, past month skin gambling predicted problem gambling (OR: 55.208 advertised sample and OR: 24.379 qualtrics sample). |
| 147 | Lelonek-Kuleta, B | Poland | 2021 | Empirical | Cross-sectional | No | Examine the risk of gambling disorder within a group of esport bettors | 438 | 33.1±9.27 | 37% | Correlation/Comorbidity | Within the sample, 17.1% reported at least once a week of esports betting and 1.6% daily betting. Of all the esports bettors, 34.9% were identified as problem gamblers, and 27.6% as moderate gamblers. Most common form of online gambling other than esports betting was TS lottery and scratch cards for offline gambling. Amount of money and duration spent for esports betting predicted problem gambling significantly, but not frequency and amount of virtual currency. |
| 148 | Luquiens, A | France | 2021 | Empirical | Cross-sectional | No | Assess the characteristics of patients accessing a helpline service | 10,017 | - | 39.90% | Correlation/Comorbidity | Of 14,564 calls made, 46 (0.3%) were about concurrent gaming and gambling problems. 2.3% were on gaming problems only and 85.7% on gambling problems only. |
| 149 | Steinmetz, F | Germany | 2021 | Empirical | Cross-sectional | No | Explore the commonalities and distinctions between pay-to-win gambling and other gambling forms | 5,315 | 18-50+ | - | Correlation/Comorbidity | Among the sample, 1508 participants practiced pay-to-win with only 124 being daily P2W buyer. Around 700 (46.4%) participants of the P2W group also gambled in other monetary forms. Frequency of P2W and cumulative spending as well as problematic P2W risk scores predicted PGSI gambling score. |
| 150 | Ayala-Rojas, RE | Spain | 2021 | Empirical | Cross-sectional | No | Examine the mechanism of concurrent gaming and gambling disorder | 117 | 24.7±11.3 | 9.40% | Correlation/Comorbidity | Within the clinic, gaming disorder prevalence was 2.46%, from 1.1% (2005) to 5.6% (2019). Among the 117 gaming disorder patients, 7 met criteria for problem gambling while 17 for gambling disorder. Age of onset for gambling, biological age, novelty seeking, and being employed all predicted concurrent gaming-gambling. |
| 151 | Close, J | UK | 2021 | Empirical | Cross-sectional | No | Scrutinize the relationship of loot box involvement and its risk factors | 16,196 | 18+ | 59.70% | Correlation/Comorbidity | Among the sample, 2780 ever purchased loot box in the past 12 months. Of all gamers, 30.1% also gambled, mostly online gambling than social casino, and 39.54% spent money for in-game purchase. Of those who gambled, 45.97% purchased loot box and 36.07% made in-game purchases. |
| 152 | Greer, N | Australia | 2021 | Empirical | Cross-sectional | No | Compare the risk of gambling problems and harms between esports bettor and tranditional gamblers | 598 | 38.92±12.03 | 28.80% | Correlation/Comorbidity | PGSI scores were higher for esports bettor (10.03±6.59) than sports bettor (3.70±4.91), with 64.8% of esports bettor categorized as problem gamblers and only 17.3% of sports bettor. Additionally, esports bettor was identified with more harms than sports bettor (81.9% vs 45.3%). |
| 153 | Marchica, L | Canada | 2021 | Empirical | Cross-sectional | No | Assess the link between esports betting, problem gambling and problem gaming | 1,348 | 14.67±1.73 | 36% | Correlation/Comorbidity | Within the study sample, 20% had joined esports gambling in the past year. Esports betting was correlated to psychological symptoms through problematic video gaming but not problematic gambling. |
| 154 | Macey, J | Finland | 2021 | Empirical | Cross-sectional | No | Investigate the varying sociodemographics and consumptions pattern among esports bettors | 1,368 | 37.83 | 41.60% | Correlation/Comorbidity | 44.8% watched esports and played video games in the past year, while 52.1% gambled at least once in the past year. Esports consumption had significant link to amount of esports bet (B: 0.268) and bets on gambling sites (B: 0.250). Similarly gambling consumption was significantly associated with esports bet (B: 0.241) and bets on gambling sites (B: 0.199). Video game consumption was not significantly associated. |
| 155 | Mason, L | UK | 2021 | Viewpoint/Commentary | Commentary | No | Discuss on the trend of laws governing video gaming with gambling-like activities that may affect youths | - | - | - | Correlation/Comorbidity | Belgium has banned all loot boxes from video games and the Netherlands ruled that loot boxes is acknowledged as gambling by the law, while Isle of Man required special licensing for including loot boxes in video games and is prohibited to be played by under 18 year olds. |
| 156 | Perreault, GP | Austria | 2021 | Empirical | Cross-sectional | No | Understand the motivations of players purchasing loot boxes in a Final Fantasy game | 592; 21 | <18-80+ | 9.40% | Converging mechanics | Gamers defined loot boxes as resources that is occurring in a shared community, require time and resource management skills both in and out of the game, and require large time investment. The chance-element of loot boxes provide a sense of success and gratification when gamers received low probability (rare) items. Particular game mechanics limit free gameplay duration before a set duration of delay, which can be reduced by purchasing more 'energy'. Strategy is thus required to balance the two, including managing time in the real world. Participants also note on the importance of forums, board communities, and social media to gain information, naturally gamers will know each others' progress and item procurement which propagate competition. Additionally, central leading figures are produced, whom will have more knowledge and privileged roles. The social platforms might direct gamers to purchase using real money if it is required to progress. |
| 157 | Wardle, H | UK | 2021 | Review | Book | No | Review the convergence of gaming-gambling from its social and historical aspects | - | - | - | Correlation/Comorbidity | The modern convergence between gaming and gambling have exploded, but it can be appreciated that the relationship has been growing for a long time. Industry players are also diversifying into both gaming and gambling ventures, thus blurring their labels and responsibilities. |
| 158 | Duffy, L | Australia | 2021 | Review | Literature Review | No | Summarize the research on gambling among youths, with the addition of its convergence with video games | - | - | - | Correlation/Comorbidity | The convergence of gaming and gambling are mediated through multiple platforms, including video gaming modules, loot boxes, and esports betting. Australian surveys have demonstrated its usage, impact, and harm on youths and adults. In response, recommendations on more stringent regulation have also been made, especially with the disturbing marketing strategy. |
| 159 | Nicklin, LL | UK | 2021 | Empirical | Qualitative | No | To gain in-depth understanding on motivations toward risky behaviors in gaming and gambling | 28 | 28.3±8.98 | 40.91% | Correlation/Comorbidity | The study identified 7 themes pertaining risky behaviors of gaming and gambling, the experience of opening items (loot box related), items value (aesthetic, functional, and monetary), gameplay related (pay to play, pay to win, investment, skipping grind), social influence (status and esteem), emotive/impulsive (urge, lack of control, boredom, escape, hard to describe), and fear of missing out (limited promotions) |
| 160 | Rockloff, M | Australia | 2021 | Empirical | Cross-sectional | No | Examine the correlation of loot boxes usage to gambling practice and harm | 1,954 | 12-24 | 59.90% | Gateway effect | Purchasing and selling loot boxes were associated significantly with gambling frequency, PGSI scores, and Gambling Harm Scores among adults. In adolescents, purchasing loot boxes was associated with DSM-IV-MR-J for gambling problems. For adolescents girls the act of selling loot boxes and number of years since opening first loot boxes were also associated. |
| 161 | Kolandai-Matchett, K | New Zealand | 2021 | Review | Narrative Review | No | Review the convergence phenomenon and summarize the commonalities | - | - | - | Correlation/Comorbidity | Several identified convergence areas are gaming elements in gambling, gambling elements in gaming, gambling on games (or using virtual items), free simulated online gambling, and social media games/gambling. Most legislative measures have focused on loot boxes, legal hesitancies remain due to lacking harm data. |
| 162 | Carey, PAK | Australia | 2021 | Empirical | Cross-sectional | No | Measure harm in loot box involvement and gambling | 471 | 18-30 | 15% | Correlation/Comorbidity | Of all types of expenditures, only loot box expenditure was correlated to any harm or moderate harm (r: 0.18-0.21). Compared to PGSI (0.20-0.42), IGD had higher correlation to harm (0.46-0.59). |
| 163 | Spicer, SG | UK | 2021 | Review | Systematic Review | No | Summarize evidence for relationship between loot box and problem gambling | 13 | - | - | Correlation/Comorbidity | 12 out of 13 publications reported positive relationship of loot box to problem gambling, with a small mean effect r: 0.27. The loot bot problem video gaming had higher effect size of r: 0.40, while problem gaming and problem gambling was r: 0.21. Direction of causality will require more evidence. |
| 164 | Stark, S | Canada | 2021 | Empirical | Cross-sectional | No | Scrutinize the convergence of gambling and gaming behavior | 2,651 | 8-24 | - | Correlation/Comorbidity | 8.5% of adolescent and 12.0% of young adults play games for virtual credits (gambling-like). 0.8% of adolescents played gambling game for real money. Most purchased gaming feature was specific item for avatar (39.4%), gain more in-game currency (38.8%), and prolong game play (20.6%). History of gambling or social casino games increased likelihood of spending money in-game, while playing video game for money was linked with higher gambling problems. Parental reports and concerns also associate significantly with children's and adolescent's that purchased more in-game. |
| 165 | Close, J | UK | 2021 | Empirical | Cross-sectional | No | Identify whether loot box purchaser had higher income or are problem gamblers | 7,771 | 16-18+ | - | Correlation/Comorbidity | Significant correlation of problem gambling and loot box expenditure, rho: 0.33. 5% of the biggest spenders on loot box represents >50% of the industry revenue from loot boxes. These high-spending players also demonstrate significantly higher PGSI scores. There is not evidence of correlation between large spending to higher earnings. |
| 166 | Palmeira, M | USA | 2021 | Empirical | Cross-sectional | No | Analyze impact of microtransactions engagement to video game intensity | 208; 207; 200 | 37; 37; 37 | 36%; 36%; 41.5% | Correlation/Comorbidity | Microtransactions intended for aesthetic, than functionality, were significantly more well received by players. Hours of playing but not amount of spending influenced the favourability towards the game, particularly those with aesthetic intentions. |
| 167 | Brock, T | UK | 2021 | Viewpoint/Commentary | Editorial | No | Provide overview of upcoming evidence on gaming gambling convergence | - | - | - | Correlation/Comorbidity | Numerous studies have addressed the shifting landscape of gaming and gambling though little have analyzed from the lenses of consumption theory. Multidisciplinary approach is required as the field is rapidly evolving. |
| 168 | Derrington, S | Australia | 2021 | Viewpoint/Commentary | Commentary | No | To comment on necessary regulations addressing the microtransactions in games | - | - | - | Correlation/Comorbidity | Loot box and other microtransactions due to a token economy have posed as a challenge. Regulatory framework must ensure consumer welfare to make informed decisions when participating or condoning dependents to participate in gambling-like activities. Pilot studies of new game classifications will be required, as well as feedback mechanism from the communities and industries. |
| 169 | Garea, SS | New Zealand | 2021 | Empirical | Meta-analysis | No | Collate data on loot box spending, problem gambling, and pathological gaming | 22 studies | - | - | Correlation/Comorbidity | Overall correlation of loot box to gambling is small r: 0.26 or moderate r: 0.37 (trim and fill), and smaller to gaming (r: 0.25). |
| 170 | Whitson, J | Canada | 2021 | Viewpoint/Commentary | Case Study | No | Analyze the moral factors to regulate gambling and digital games | - | - | - | Converging mechanics | As gambling enters the virtual world, it becomes harder to regulate. Rigid dichotomies of games/gambling games, skilled/unskilled play have also become challenged. Games could garner precious data to predict players behaviors at the expense of players time investment, this variable is exploited through gambling design imperatives (time-on-device). |
| 171 | Thorhauge, AM | Denmark | 2021 | Review | Narrative Review | No | Discuss the growing skin betting economies in Epic and Steam | - | - | - | Converging mechanics | Epic maintains skin monopoly within their games, while Steam promoted free skin market that crosses platforms. This latter system aids the transition of prosumer to micro-entrepreneur and modder into speculator. |
| 172 | Joseph, D | UK | 2021 | Review | Narrative Review | No | Examine how capitalism of battle pass is a form of monetized gaming/gambling | - | - | - | Converging mechanics | Battle passes, loot boxes, and other monetization in games are particularly interlocked among battle royale games. Battle pass capitalism portray the image that games are shops. Even on platforms such as Steam, players compete to gain discounts on game prices. The continuation of these entry passes will drive games not built on these service model premises as unacceptable risk. |
| 173 | Jarrett, J | UK | 2021 | Empirical | Qualitative | No | Explore the microtransactions of League of Legends as examples of affective economy and commercial exchange | 49 | - | - | Converging mechanics | Designing a fair F2P could strengthen an affective economy among the player communities. Games such as LOL is experiences across platforms and spaces through multiple interactive activities. Gaming industries can monetize and commoditized games through an affective agenda to expand from a negative for-profit image. Players expressed the motives for microtransactions engagement as a form of support to the game and a mutually constituted relationship of developer/players. |
| 174 | Macey, J | Finland | 2021 | Empirical | Cross-sectional | No | Understand the effect of exposure to gamblified media affects gambling activities among esports fans | 255 | 12-47 | 18% | Correlation/Comorbidity | Only 2 respondents reported not playing games. 13% reported that the event was their first esports event attendance. 33% disclosed to watching esports regularly every week. 64% reported no involvement in gambling activities within video games or esports, while 8% have done so more than once a week. Most common is esports betting (31%) then loot box (22%) and skin betting (14%). Hours of watching esports online and frequency of watching esports online were found to associate significantly with gambling participation (V: 0.408 and 0.218). Age was negatively correlated to esports betting, while frequency of watching esports was strongly positive. Similarly for loot boxes and skin betting. |
| 175 | Denoo, M | Belgium | 2021 | Empirical | Cross-sectional | No | To describe the motivations of esports bettors using skins or monetary instruments | 87 | 18-34 | 0% | Converging mechanics | Skin betting provided sense of security as it was procured through in-game playing. On the other hand, monetary betting was more convenient and efficient. Ability to gain money from skin betting provided sense of self-esteem, social esteem, and pleasure. This also provided stimulation and excitement. |
| 176 | Freitas, BDA | Spain | 2021 | Empirical | Cross-sectional | No | To assess the varieties of disreputable behaviors existing that present as threats to esports sponsors | 1592 | 23.8±6.8 | 8.30% | Converging mechanics | Connection to illegal or unregulated gambling in esports were seen as high threat. This include promoting underage gambling, disregarding local country legality status of gambling, unregulated skin buying, or loot box purchasing. |
| 177 | Rossi, R | UK | 2021 | Empirical | Cross-sectional | No | To describe the pattern of gambling advertisements, as well as comparison for sports and esports ads | 890,000 ads | - | - | Converging mechanics | Overall only 7.3% of tweets contain social responsibility messages. Among esports ads, staggeringly only 2% contain such messages. Esports ads were also designed to be more appealing (17%) to children than traditional sports gambling ads (3%). The authors noted 76% of esports tweets contain cartoons or animations. Esports gambling accounts had 17% child followers (0-15 years old) making up their total followers, compared to 7% for traditional sports gambling accounts. |
| 178 | Ide, S | Japan | 2021 | Empirical | Cross-sectional | No | To analyze the link between loot box and gambling among adolescents and parents | 1615 | 14 | 36.80% | Converging mechanics | Purchasing loot boxes was linked to a higher chance of displaying problematic online gaming behaviors (OR= 3.75), with this association being especially strong among female participants (OR= 6.73). |
| 179 | Xiao, LY | UK | 2021 | Viewpoint/Commentary | Letter to Editor | No | To assert perception and definition of loot box mechanism as a form of gambling | - | - | - | Converging mechanics | Loot box research has a considerable gap. Academics and regulators have disparate take on the issue. Loot box definition can be broadened with viewing players as gamblers and video game company as the seller, both parties participating in the gamble. Players are aware and willing to lose money for a chance of obtaining items with varying degree of valuation at random/chance. |
| 180 | Xiao, LY | UK | 2021 | Review | Literature Review | No | To review and address the requirement to regulate loot box | - | - | - | Converging mechanics | Loot box can be considered as a form of gambling. In spite of that, loot box is very poorly regulated. Disclosure of loot box or probabilities as well as setting maximum limit might be beneficial. |
| 181 | Hing, N | Australia | 2022 | Empirical | Cross-sectional | No | Simulated gambling participation (i.e., gambling-like activities in video games), associated risk to problem gambling, and association to monetary expenditure as well as time spent playing among youths | 826 (Sample 1) and 843 (Sample 2) | 14.81 and 14.61 | 44.8% and 30.6% | Correlation/Comorbidity | Model 3 (Games with Mini Gambling Components) explained 46% dan 65% variance of problematic gambling in the two samples, respectively. Across both samples, these games are also linked significantly to monetary gambling. In turn, expenditure and time spent playing were highly associated with explained variance being 35% and 50% toward gambling problems. |
| 182 | Lischer, S | Switzerland | 2022 | Viewpoint/Commentary | Narrative Review | No | To provide a summary of games, along with the possible structural properties, that act as risk factors to problematic gambling | - | - | - | Converging mechanics | Monetization within video games tend to be predatory, with gamblification aspects, for example loot boxes. These activities mimic gambling through provision of chance activities. Industry self-regulation have been lacking compared to other aspects of games, e.g., violence. |
| 183 | André, F | Sweden | 2022 | Empirical | Cohort | No | Relapse prevention feasibility among youths with disordered gaming and problematic gambling | 9 | 13-17 | 11% | Correlation/Comorbidity | 7 participants were screened positive for disordered gaming and 3 for problematic gambling before the start of treatment. The study could not conclude efficacy of relapse prevention for both gaming and gambling outcome. Participants reported acceptability and feasibility of the program. |
| 184 | Wardle, H | UK | 2023 | Empirical | Cohort | Yes | Investigating the risk of gambling-like practices in digital games to problematic gambling behavior | 2080 | 21.5 | 46.4% | Gateway effect | Incidence rate ratio for loot boxes involvement is 1.58 (1.07-2.33) and skin betting 2.14 (1.40-3.29). Through parsimonious model, skin betting is still significant at 2.32 (1.69-3.19). There are no significant wave interaction to neither skin betting nor loot boxes. |
| 185 | Drummond, A | New Zealand | 2022 | Empirical | Cross-sectional | No | Investigate the effect whether engagement in probabilistic in-game activities such as loot box causes harm in the form of psychological distress | 2432 | - | 54.1% | Correlation/Comorbidity | Around 43.9% loot box purchasers reported severe psychological distress. On binomial regression the relative risk (RR) is 1.79 (1.53-2.05) for severe distress among players who bought loot box than those who did not. Loot box attributed distress is higher than other purchase types and the only one statistically significant. A substantial amount of variance is explained by problem gambling symptoms. |
| 186 | King, A | USA | 2022 | Empirical | Cross-sectional | No | Investigate whether overlap of gambling and gaming also affect motivations of playing games, particularly through financial incentives | Study 1: 797 Study 2: 179 | Study 1: 20.3±4.54 Study 2: 41.87±10.16 | Study 1: 62.9% Study 2: 48.6% | Converging mechanics | Financial motives are correlated to gaming disorder severity (r= 0.15-0.26, p<0.05). When participants have low to moderate upward mobility perception, financial motives and GD severity were significantly linked (B: 1.07, p<0.001). |
| 187 | Jouhki, H | Finland | 2022 | Empirical | Cohort | Yes | To determine how escapism predict excessive gaming or gambling and how both behaviors change over time | 1022 | 49.5 | 48.43% | Correlation/Comorbidity | Over time, scores of excessive gaming and gambling did not differ significantly. At baseline, excessive gaming and excessive gambling are correlated (r= 0.53, p< 0.001). Longitudinally, respondents with excessive gaming also reported excessive gambling (B: 0.23, p< 0.001) and vice versa (B: 0.21, p< 0.001). |
| 188 | Costes, JM | Canada | 2022 | Empirical | Cross-sectional | No | To describe the risk of spending money in free-to-play games | 3472 | 39.5 | 48.50% | Converging mechanics | Increase money spent on games is associated with disordered gaming. Spending money is also linked to higher flow (immersion), escapism, and competition. Some gamers demonstrated exhibited negative urgency, sensation seeking, and lack of perseverance. Authors note that these are similar phenomena in gambling, thus the gaming industry require similar standards of regulations. |
| 189 | Szerman, N | Spain | 2022 | Empirical | Cross-sectional | No | To investigate the hypothesis of conceptualizing gambling disorder as gambling dual disorder | 116 | 39.2±11.5 | 10.30% | Correlation/Comorbidity | The participants were all gambling disorder patients attending a treatment center. Around 11.2% (N=13) out of all the participants also had video game addiction. Of which, six were probable existence, two were very probable, and five were definite. Clustering analysis of to differentiate between severity of gambling disorder demonstrated non-significance for video gaming addiction. |
| 190 | D'Amico, NJ | Australia | 2022 | Empirical | Experimental | Yes | To explore one mechanism that might explain the gateway effect from gaming to gambling | 153 | 25±6 | 38.6%% | Gateway effect | Loot box versus control yielded a BF of 8.77 towards null hypothesis, similarly for fixed reward versus control and loot box versus fixed reward. On the other hand, correlation analysis demonstrated significance between past loot box engagement and gambling symptoms (r= 0.35). |
| 191 | Baggio, S | Switzerland | 2022 | Empirical | Cross-sectional | No | To assess varieties of problematic online behavior including gaming and gambling | 1617 | 37.2±12.3 | 51.60% | Correlation/Comorbidity | Problematic gaming is significantly correlated to problematic gambling (r= 0.183, p<0.001). Although problematic gaming is more correlated to problematic cybersex (r= 0.236, p<0.001). In the network analysis, between-community edge's strength across problematic gaming and problematic gambling is only 7.8%. |
| 192 | Burleigh, TL | UK | 2022 | Empirical | Cross-sectional | No | To investigate commonalities of grouping through naturalistic factors for behavioral addiction and the possible combinations of significant factors to each problematic behavior | 1916 | 18-64 | 48.60% | Correlation/Comorbidity | High risk profile in UK cohort demonstrated high mean score difference for gaming (0.86) and gambling behavior (1.00) than standardized mean. In contrast, New Zealand and Australian cohorts did not show this pattern. Whereby the high-risk profile group only had moderate video gaming score difference to mean (0.47-0.51) but large gambling score difference to mean (0.80-1.00). |
| 193 | Budiman, R | Indonesia | 2022 | Review | Narrative Review | No | This research seeks to analyze the behavior of Indonesian adolescents who are addicted to online gambling. | - | - | - | Gateway effect | Adolescents addicted to online gambling exhibited the following behaviors: spending increased amounts of time engaged in online gambling, reduced physical activity and neglect of health, avoidance of significant life activities, diminished social interactions, difficulty resisting the urge to gamble online, neglect of work and personal responsibilities, and signs of mental illness. |
| 194 | Lubis, AA | Indonesia | 2022 | Empirical | Qualitative | No | To explore the connection between online gambling and online gaming among students and teenagers in Pasar V Tembung Village. | 15 | - | - | Gateway effect | The authors note that various forms of online gambling disguised as online games are being conducted by students in Pasar V Tembung Village, including slots, lotteries, applications, links, websites, Higgs Domino, football betting, and poker. Teenagers engage in online gambling as a means of fulfilling their psychological and material needs. |
| 195 | Dagwi, N | South Korea | 2022 | Empirical | Cross-sectional | No | The study examined varying perceptions of eSports genres and their impact on the consumption of eSports content among regular internet viewers and enthusiasts globally. Additionally, the paper investigated the perceptions associated with specific genres, including Multiplayer Online Battle Arena, sports simulations, tactical shooters, and card games. | 371 | 18 to above 45 | 42.30% | Correlation/Comorbidity | The results indicate that gambling is statistically insignificant and negatively correlated with watching eSports. This suggests that, unlike traditional sports, there are no established platforms for spectators to predict winners with the potential for substantial financial gains. |
| 196 | Balhara, YPS | India | 2022 | Review | Literature Review | No | This study provides an overview of the current state of fantasy sports and its implications regarding addictive behaviors within diagnostic frameworks. | - | - | - | Converging Mechanics | There is a lack of consensus regarding the classification of fantasy sports as either ‘gaming’ or ‘gambling.’ Additionally, diagnostic systems do not provide a clear definition of ‘gaming behavior.’ |
| 197 | Declerck, P | Belgium | 2022 | Review | Systematic Review | No | This report aims to present a structured overview of gambling and online gaming within the context of the existing children’s rights framework at both international and European levels. | - | - | - | Gateway Effect | Video games featuring microtransactions, such as in-game purchases, are becoming increasingly integrated into children’s daily lives. As a result, children are encountering more gambling-like elements in these games, which could be potentially harmful. Currently, there is a wide array of regulations relevant to this issue, both in general and specifically for children. However, the legal landscape is fragmented, with existing frameworks often overlapping, which complicates the understanding of the interrelations among these provisions in practice. |
| 198 | Riatti, P | Germany | 2022 | Review | Scoping Review | No | This scoping review investigates the existing evidence and identifies the potential societal impacts of esports by utilizing the Mapping Elite Sports Societal Impact Model. | - | - | - | Correlation/Comorbidity | In the context of fans and media attraction, one study noted that eSports consumption can serve as an indicator for gambling and, ultimately, gambling disorder, particularly among young males. The current eSports gambling and betting market is largely unregulated, making it vulnerable to irregularities, match-fixing, and betting abuse. |
| 199 | Daglis, T | Greece | 2022 | Empirical | Cross-sectional | No | To examine the impact of the COVID-19 pandemic using a financial framework by analyzing the stock performance of gaming and online gambling-related companies and considering the activities of these companies. | - | - | - | Correlation/Comorbidity | Events related to COVID-19 have impacted the stock prices of gaming and gambling companies, indicating that confirmed COVID-19 cases offer valuable insights for interpreting and modelling these stocks. The multifractal spectrum analysis of the cross-correlation between each stock and the confirmed COVID-19 cases reveals that the Hurst exponent values for positive q orders are close to or exceed 1.5, indicating a strong relationship. In contrast, the Hurst exponent values for the respective negative q orders are close to or above 2.5, reflecting a very strong relationship. |
| 200 | Lemmens, JS | Netherlands | 2022 | Empirical | Cross-sectional | No | To describe the intentions of an online sports game for spending money on loot boxes | 1144 | 24.0±7.29 | 6.20% | Converging mechanics | Players with gaming disorder tend to spend more on loot boxes (r= 0.14, p< 0.001). Money spent on loot boxes also is greater with higher reward sensitivity (r= 0.21, p< 0.001) and competitiveness (r= 0.17, p< 0.001). Gaming disorder association with higher money spent is mediated by need for autonomy (b= 0.07, p<0.05). Players also associated higher spending with increased chance of winning or higher ranking (b= 0.12, p<0.001). |
| 201 | Ramella-Zampa, B | Italy | 2022 | Review | Narrative Review | No | To provide overview of esports and the association of spectatorship and consumption to behavioral addiction | - | - | - | Converging mechanics | Some features in esports have been gamblified. For example in-game currencies that can be used to purchase items at random. Esports spectatorship also is associated with increased odds for risky gambling and experiencing gambling harm. |
| 202 | Tavares, R | Portugal | 2022 | Review | Narrative Review | No | To address the use of non-fungible tokens in triple A games | - | - | - | Converging mechanics | Play-to-earn games may be perceived as gambling games by some players. Trading financial assets may include microtransactions with intermediary currency such as the NFTs. |
| 203 | Garrett, EP | Australia | 2022 | Empirical | Cross-sectional | No | To investigate the association between loot box involvement and problematic gambling | 1049 | - | 63.20% | Converging mechanics | Higher income is linked to higher loot box purchase. Both income and problematic gambling symptoms fit the model explaining loot box spending, however, neither significantly correlated to loot box purchase. |
| 204 | Cena, L | Italy | 2022 | Empirical | Cross-sectional | No | Explore the prevalence of gaming and gambling disorder along with the types | 502 | 15.9±1.93 | 67.70% | Correlation/Comorbidity | From the study sample, 40.8% were gamers, 4.8% gamblers, and 17.8% were both video gamers and gamblers. Of those who played video games, 7.5% were problem gamers. And of those who gambled, 5.3% were problem gamblers. Only 0.2% of all participants met criteria for problem gamers and gamblers. |
| 205 | Gomez, R | Australia | 2022 | Empirical | Cross-sectional | No | Investigate the factor structure of concurrent addiction disorders | 968 | 29.5±9.35 | 32.50% | Correlation/Comorbidity | Gaming addiction was moderately correlated to online gambling addiction, r: 0.38, p<0.001 |
| 206 | Greer, N | Australia | 2022 | Empirical | Cross-sectional | No | Assess the motivations for esports betting and skin gambling along with the relationship to gambling variables | 736 | 28.98±8.07 | 19.80% | Correlation/Comorbidity | Among the study sample, 30.4% were problem gamblers. Similar proportions among esports cash bettor 29.3%, esports skin bettor 30.6%, and skin gambling 33.8%. Among the esports cash bettors, 86.6% were motivated for winning money and 80.1% for the excitement while 71.8% were for enhancing enjoyment while watching esports. Comparatively, for esports skin bettor the motivations were for excitement (78.9%), to collect skin (74.4%), and use skins during play (72.2%), as for financial win was only 61.7%. For skin gamblers, it was similar with 74.8% saying for excitement, 72.0% to collect skin and 71.7% for using skin during gameplay with only 65.8% aiming to win money or 68.0% winning skin to be exchanged with money. Motivations for regulating internal states were able to predict gambling harms across all groups. |
| 207 | Greer, N | Australia | 2022 | Empirical | Cross-sectional | No | Study the relationship between video game involvement and gambling-related activities to gambling harm and problems | 737 | 28.97±8.07 | 19.80% | Gateway effect | Esports viewing and purchasing skins in the past 6 months were predictive of esports cash betting, esports skin betting and skin gambling frequencies. Esports cash betting was also predictive of traditional monetary gambling. Though only sports betting was predictive of video game-related gambling. Lastly, skin gambling was predictive of at least moderate problem gambling and gambling harm category 4 (5-10 harms). |
| 208 | Hing, N | Australia | 2022 | Empirical | Cross-sectional | No | Scrutinize the relationship of esports betting to video game, monetary gambling, and problem gambling | 1667 | 14.6±1.7 and 14.8±1.6 | 30.7-44.8% | Gateway effect | Playing esports game, watching esports and competing in esports were predictive of esports skin betting in both sample groups. Impulsiveness and problematic gaming were not consistently associated in both groups. But impulsiveness is correlated to past month esports cash betting. Esports skin betting was also associated with problem gambling in both groups. |
| 209 | Hing, N | Australia | 2022 | Empirical | Cross-sectional | No | Examine the association of loot box purchase with problem gambling | 1669 | 14 | 30.6-44.8% | Gateway effect | Esport betting was associated to past month loot box purchase. Past month loot box spending was also correlated to problem gambling (r: 0.23-0.39). Past month loot box purchase was predictive of at-risk (OR: 4.27-10.50) and problem gambling (OR: 3.73-6.00). |
| 210 | Macía, L | Spain | 2022 | Empirical | Cross-sectional | No | Explore the links of gambling across affects, motives and other addictive behaviors in women | 351 | 12-26 | 100% | Correlation/Comorbidity | 39 (11.1%) had gambling problems. This group did not demonstrate difference in video game addiction score than non-problem gamblers, gambling severity was also not correlated to video game addiction. |
| 211 | Montiel, I | Spain | 2022 | Review | Scoping review | No | Review the relationship between loot boxes to problem gaming and problem gambling | - | - | - | Correlation/Comorbidity | Prevalence of loot box purchase is higher in adults gamers than adolescent gamers. Overall, there is a possible significanty relationship between loot box purchase to problematic gaming and gambling. |
| 212 | Müller, SM | Germany | 2022 | Empirical | Cross-sectional | No | Provide consistent measure for various addictive behavior | 985 | 47.6±14.5 | 46.50% | Correlation/Comorbidity | Online gaming and online gambling had moderate correlation of r: 0.322-0.434 |
| 213 | Puiras, E | Canada | 2022 | Empirical | Cross-sectional | No | Scrutinize the motives for involvement in loot box purchase and gambling | 540 | 26.17±9.16 | 30.50% | Correlation/Comorbidity | Motive themes for gambling were enjoyment, winning chance, escape boredom and charitable intentions. While for loot boxes were enjoyment, winning opportunity, progressing the game, and passive engagement. Motives for refraining from gambling and loot boxes were similar, e.g., negative consequences, uncertain outcomes, concerns of gambling, disinterest, (lack of) finances, and accesibility. |
| 214 | Raneri, PC | Australia | 2022 | Review | Systematic Review | No | Review the associations between microtransactions, gaming, and gambling | - | - | - | Correlation/Comorbidity | There is evidence of positive relationship between microtransactions to gaming and gambling disorder, particularly loot boxes and risky loot box pattern. Limited evidence suggests adolescents are at higher risk from loot box purchase to develop problem gambling. |
| 215 | Savolainen, I | Finland | 2022 | Empirical | Cross-sectional | No | Examine the impact of social distancing to gaming and gambling disorders | 1,530 | 46.67±16.42 | 49.67% | Correlation/Comorbidity | Gaming and gambling problems were correlated (B: 3.6, p<0.001) and predicted by social motives (more so for gaming problems than gambling) during social isolation. |
| 216 | Sidloski, B | Canada | 2022 | Empirical | Cross-sectional | No | Investigate loot box and in-game gambling elements understanding among gamer or gambler populations | 144-2,278 | 35.6-37.6 | - | Correlation/Comorbidity | 7 gamers who did not gamble and did not purchase loot box had positive PGSI score, while 15 gamers who did not gamble but purchase loot box also scored positive on PGSI. 8 gamers with score of 1-2 on PGSI endorsed loss chasing, 4 (moderate risk), and 21 from high risk PGSI. For those who purchased loot box, 7 from low risk PGSI, 7 from moderate PGSI, and 9 from severe PGSI. This is possible that gamers consider loot boxes as gambling or relate to legacy harms when filling out PGSI. |
| 217 | Spicer, SG | UK | 2022 | Empirical | Cross-sectional | No | Assess the gateway effects and reverse gateway effects of lootbox purchase and gambling disorders | 1,102 | 32.1±9.57 | 48.40% | Gateway effect | Both gateway and reverse gateway effects were self-reported equally by 19.87% of the participants. 19.63% of those purchasing loot box first transitioned to gambling, and 20.11% of those who gambled first tried loot box purchase. Proportion of males reporting gateway effect was higher than females but not so for reverse gateway. Loot box was correlated to problem gambling and problem video gaming. Higher scores on gambling cognitive errors was correlated to both gateway and reverse gateway effects. Income was not correlated to loot box spending amount. Gateway effect was commonly cited due to sensation seeking and normalization/habituation, while the reverse due to sensation seeking and safer perception. |
| 218 | Vuorinen, I | Finland | 2022 | Empirical | Cross-sectional | No | Explore the level of satisfaction and frustation pertaining gambling and gaming problems | 1,530 | 46.7±16.4 | 49.40% | Correlation/Comorbidity | Gaming and gambling problems were moderately correlated, r: 0.53, p<0.001. Need frustration was correlated to both presence of gaming and gambling problems. |
| 219 | Zarate, D | Australia | 2022 | Empirical | Cross-sectional | No | Scrutinize the relationship across 10 forms of addictive disorders | 968 | 29.54±9.35 | 33.60% | Correlation/Comorbidity | Internet gaming was correlated to problem gambling, r: 0.379, p<0.001. Gambling symptoms demonstrated highest centrality along with certain substance use disorders symptoms. On the disorder level, gaming disorder is the third most central, indicating potential high comorbidity in clinical practice. |
| 220 | Ciccarelli, M | Italy | 2022 | Empirical | Cross-sectional | No | Asses the commonalities between internet gaming disorder and gambling disorder among adolescents | 366 | 16.2±1.33 | 47.80% | Correlation/Comorbidity | IGD and GD have significant small correlation, r: 0.334, p<0.001 |
| 221 | Close, J | UK | 2022 | Empirical | Cross-sectional | No | Determine the impact of COVID-19 lockdown on gamers and gamblers | 631 | 45.28±15.33 | 50.55% | Gateway effect | 44.85% of the sample played games and gambled. Amount of spending on loot box during lockdown increased significantly and so the average IGD score, however risky loot box index score did not increase. IGD score before lockdown was predictive of PGSI after lockdown; however, the reverse was not significant. |
| 222 | Gibson, E | UK | 2022 | Review | Systematic Review | No | Summarize the evidence of links between microtransactions in games, problem gaming, and problem gambling | 19 | - | - | Correlation/Comorbidity | Across varying demographics, loot box purchase and problem gambling is correlated. The correlational nature limits causality interpretation. Association of other microtransactions to problem gambling is understudied. |
| 223 | Woods, O | Singapore | 2022 | Review | Narrative Review | No | Provide counterarguments on the gambling-like elements of gacha in games | - | - | - | Converging mechanics | Gacha is a system of accesing a chance-based source that gives out random items (usually at lower value than the inserted amount) to progress with the game. There is possibility players 'game-the-game' and the 'monetized' design is sought after for aspect of excitement and challenge, by repetitive playing or 'grinding'. |
| 224 | Hing, N | Australia | 2022 | Viewpoint/Commentary | Commentary | No | Discuss the gamblification of video games and its risk to adolescents | - | - | - | Correlation/Comorbidity | Gambling definitions need to reconceptualized to encompass relevant psychological and addictive domains involved with the activities, respecting that value does not equate only financial aspect. The gamblification of digital media and games have been subtle thus regulations have been lagging behind. Priorities are required for data on gateway effect, harms and risk factors |
| 225 | Woods, O | Singapore | 2022 | Empirical | Qualitative | No | Explore the emotive and psychological aspects of gacha | 21 | - | - | Converging mechanics | Gacha can produce items, skins, and other virtual items which is becoming very popular in Singapore such as through Genshin Impact or third party websites. Due to the subtleties in gacha system, players do not notice the correlation to gambling at face value until it becomes problematic. The affective relationship from gacha through to the avatar creates a complex relationship, even socially to other players. A relationship that may provide emotional payoff for the players. |
| 226 | Xiao, LY | Denmark | 2022 | Empirical | Letter to Editor | No | To summarize the spread of loot box among video games in the market | 100 | - | - | Converging mechanics | 77% of the surveyed top grossing games contain loot box. Several identifications were different from previously published studies. Some loot box forms are unique and some in-game gambling like activities allow for use of real-world currencies. This include seemingly benign games, such as claw machines. |
| 227 | Amadieu, T | France | 2022 | Review | Book | No | To provide a review of the cultural dynamics in China that affect gaming culture | - | - | - | Converging mechanics | Some Chinese regulations such as banning home consoles has led to a more harmful video game market. The Chinese game system has adopted "guochanyouxi", a type of design that push monetization through lotter-type devices and provide a service rather than product. Pity-timers is feared to increase cognitive bias since certain rarer rewards are ascertained after a set amount of trials. |
| 228 | Etchells, PJ | UK | 2022 | Empirical | Cross-sectional | No | To examine the association of loot box spending to problem gambling and psychological states | 1990 | 27.9±9.0 | 25.90% | Converging mechanics | Using Bayesian analyses, loot box spending is significantly associated with problem gambling (r= 0.201, BF >10). Loot box spend was not linked to psychological distress, but non-gambling game-related spending was significantly associated (r= 0.046, BF >10). |
| 229 | Aagaard, J | Denmark | 2022 | Empirical | Qualitative | No | To assess players' and developers' perception of dark designs in gaming | 16 | 22-40 | 37.50% | Converging mechanics | Participants agree that the darkest design in modern video game is gamblification, such as loot box. This is rated darker than impersonation or friend spam. While brightest design is reciprocity and grinding feature. |
| 230 | Primi, K | Italy | 2022 | Empirical | Cross-sectional | No | To investigate use of loot box relations to gambling frequency and problem gambling through mediation of video gaming | 1078 | 16.46±1.28 | 61.00% | Gateway effect | Loot box use was significantly associated to video gaming frequency (b= 0.44, p< 0.001) then to problem video gaming (b= 0.40, p< 0.001), and then to problem gambling (b= 0.34, p< 0.001). This was consistent before and during lockdown. Problem video gaming and problem gambling are moderately correlated (r= 0.45, p< 0.001). |
| 231 | Clark, L | Canada | 2023 | Viewpoint/Commentary | Narrative Review | No | To translate effect of reward variability in modern gambling to other behavioral addictions, including gaming. | - | - | - | Converging mechanics | Variability is maintained by leveraging gameplay difficulty across levels, thus preserving the flow (state of immersion) even for skilled players. Players also attain in-game items (e.g. characters, skills, weapons, etc.) through randomized prized. In contrast to conventional gambling, developers are able to conjure unlimited prizes with abstract values that are non-fungible. For example through loot boxes. |
| 232 | Saini, N | Canada | 2023 | Review | Scoping review | No | Examining video games' structural properties that increases the risk for gaming disorder | - | - | - | Converging mechanics | Identified gambling-like characteristics, predominantly reward and punishment features. These include in-game currency, random chance purchase (e.g. loot boxes), event duration features, meta-game rewards, size of reward, general reward features (e.g. experience points and bank systems), intermittent rewards, near miss, punishment features, and even frequency features. |
| 233 | Seffah, KD | USA | 2023 | Review | Narrative Review | No | To review the effect of esports on cardiovascular health and mental health, including addiction such as gambling addiction | - | - | - | Correlation/Comorbidity | There are modifiable behavioral instruments in games that might increase the risk for gambling addiction. Understanding these drivers gambling will help to engineered games with less craving and addiction risk. |
| 234 | Sanmartín, FJ | Spain | 2023 | Empirical | Cross-sectional | No | To investigate the pattern of loot boxes involvement among gamers, relationship to higher spending, similarities to traditional gambling, and impact to psychoemotional states. | 475 | 19.26 | 34.95% | Converging mechanics | There was not statistically significant difference between adult and adolescent for loot boxes purchase (p= 0.25). Loot box involvement was also not significantly associated with socioeconomic status (p= 0.147) or hours spend playing (p= 0.95). Not obtaining the desired item is associated with higher purchase of loot box (p≤ 0.05). In turn, loot box use is also linked to guilt (p= 0.0001), perceived distress (p= 0.0001), and loss of control (p= 0.003). |
| 235 | Kim, HS | Canada | 2023 | Review | Scoping review | No | Assessing the relationship between gambling-like activities (GLA) to gaming or gambling as well as demographics and psychological characteristics. | - | - | - | Converging mechanics | Among empirical studies, there are positive link between GLA to problem gaming and problem gambling. These include loot boxes, esports, skin betting, and token wagering. However, screened empirical studies are only cross-sectional, thus unable to determine the directionality. |
| 236 | Xiao, LY | Denmark | 2023 | Empirical | Cross-sectional | No | Examine the correlation of loot box expenditure with gambling engagement, problem gambling, and impulsiveness | 879 | 23.0 | 19.3% | Gateway effect | Loot box expenditure is correlated to history of gambling in the past 12 months among Chinese speaking sample (p= 0.023). However, loot box expenditure is not correlated to impulsivity (p= 0.100) and problem gambling (p= 0.297). The majority of Chinese respondents (80.7%) view pity-timers as appropriate. Chinese speaking respondents are less likely to be gamblers than English speaking respondents. |
| 237 | Russell, AMT | Australia | 2023 | Empirical | Cross-sectional | No | Assess the temporal evidence of migration from simulated gambling to other gambling forms through data of age of first participation in either simulated gambling or monetary gambling | 1026 | 21.9±2.32 | 59.10% | Gateway effect | Loot boxes are associated with any form of monetary gambling in lifetime (b= 2.593, p< 0.001) and problem gambling in lifetime (b= 1.932, p<0.001). As well as monetary gambling in last 12 months (b= 2.183, p< 0.001) and problem gambling in last 12 months (b= 0.396, p< 0.001). Video games with gambling content also is significantly linked to problem gambling in 12 months (b= 0.299, p<0.01) and lifetime (b= 1.752, p<0.001). |
| 238 | Mangat, HS | Gibraltar | 2023 | Review | Systematic Review | No | To review all available empirical research on the domain of esports gambling | - | - | - | Correlation/Comorbidity | Esports are unique, in that spectatorship and participation provide opportunity to esports betting. Esports gamblers demonstrated higher scores than some traditional gambling. There is also positive relationship of harm to severity of gambling. Some esports betting are complex, such as esports skin betting. |
| 239 | Wieczorek, Ł | Poland | 2023 | Empirical | Cross-sectional | No | To explore motives and determinants of the transitioning from gaming to gambling through a qualitative study | 38 | 26.3±5.1 | 15.8% | Gateway effect | Domains of motives are separated into structure of video games and gambling (desire to win and aspect of randomness), social benefits and losses (financial and recognition), misconception of control, experienced emotions, and escape from reality. |
| 240 | DeCamp, W | USA | 2023 | Empirical | Cohort | No | Comparing the loot box involvement pattern pre- and post-COVID19 pandemic | 2019: 4425 2022: 6492 | 13-17 | 2019: 49.2% 2022: 47.3% | Converging mechanics | Among 8th grader boys, the rate of playing video games from 2019 to 2022 did not change significantly (from 92.7% to 93.6%). However, loot box purchase increased significantly from 43.7% in 2019 to 48.6% in 2022 (p= 0.013). In contrast, among 11th grade males, playing video games did spike significantly from 84.8% to 88.0% (p= 0.029) but was not followed with significant loot box involvement. Alternatively, among 8th grade girls, both playing video games (p< 0.001) and loot box involvement (p< 0.001) increased significantly, from 49.4% and 11.6% to 59.6% and 22.7% respectively. Similar pattern was observed among 11th grade girls for gaming (33.3% to 44.2%, p< 0.001) and loot box (4.5% to 8.1%, p< 0.001). |
| 241 | Yüce, A | Turkey | 2023 | Empirical | Qualitative | No | To analyze how the COVID-19 pandemic affect sports and esports betting pattern | 85 | 20-48 | 0% | Converging mechanics | Most bettors turn to esports betting during COVID-19, however they described feeling the differences. They reported experiencing higher excitement through traditional sports betting. But others illustrated that there are no significant difference during change from sports to esports gambling. While there are also respondents experiencing higher thrill through esports betting. |
| 242 | Király, O | Hungary | 2023 | Review | Literature Review | No | To thoroughly examine the interaction between gaming-related factors, individual factors, and environmental influences that contribute to gambling disorder, drawing on relevant and recent literature. | - | - | - | Gateway Effect | Video games are crafted to enhance player engagement by utilizing various psychological mechanisms, including operant conditioning. It has been noted that gaming-related elements like loot boxes and microtransactions can serve as a gateway to gambling. Individual factors are essential in the development and persistence of gaming disorder, influenced by the person’s psychological and neurobiological vulnerabilities. Additionally, environmental factors, such as family dynamics and the wider cultural context, significantly affect how individuals engage with gaming and the potential for it to become problematic. |
| 243 | Cole, JD | USA | 2023 | Empirical | Qualitative | No | This research aims to offer a comprehensive understanding of the intricate experiences of school counselors as they engage with students exploring careers in esports or iGaming. | - | - | - | Correlation/Comorbidity | The six school counselors identified a distinct sense of marginalization (harm) among students in the sample due to the perceived stigma surrounding iGaming. This theme highlighted that students might face marginalization when choosing to pursue a career in iGaming, stemming from its association with gambling, the risk of addiction, and the generally negative societal perceptions of gambling as a career path. |
| 244 | Bujnakova, E | Finland | 2023 | Empirical | Cross-sectional | No | To understand why players purchase purely aesthetic virtual items in the free-to-play game League of Legends, how frequently they make these purchases, and the motivations behind their spending. | 56 | 18 to 28 | 17.90% | Gateway Effect | The primary factors influencing the purchasing behavior of League of Legends players include the visual appeal of skins, the popularity of champions, and available discounts. Free-to-play games like League of Legends incorporate loot boxes and microtransactions. The psychological aspects of loot boxes are linked to gambling, raising concerns for many, particularly parents of children who play and spend increasing amounts of time gaming as a result. |
| 245 | Greer, NM | Australia | 2023 | Empirical | Cross-sectional | No | To analyze three gambling products associated with video games, which originated in the 2010s but have only recently gained attention in academic literature: esports cash betting, esports skin betting, and skin gambling. | 737 | 28.97 ± 8.07 | 19.80% | Converging Mechanics | eSports bettors and skin gamblers exhibited high levels of problem gambling severity, as measured by the Problem Gambling Severity Index (PGSI), and gambling-related harm, as assessed by the Severity of Gambling Harm Scale (SGHS), with a correlation coefficient of  and a significance level of . |
| 246 | Liniers, BV | Portugal | 2023 | Review | Literature Review | No | The aim is to establish relevant metrics and visualizations that can enhance existing diagnostic tools and aid in the therapeutic monitoring of problematic gaming disorder. | - | - | - | Correlation/Comorbidity | The study by IGD identifies two unique challenges when compared to other behavioral disorders such as gambling. In gaming, it is more challenging to identify individuals exhibiting problematic behavior due to the absence of direct monetary involvement. A non-addicted gambler can typically stop playing after incurring losses, while an addicted gambler may continue despite those losses. In contrast, both addicted and healthy gamers can engage in gaming without facing financial consequences, making it difficult to distinguish between the two groups. Additionally, professional gamers often accumulate many hours of play each week, further complicating the differentiation of player profiles. |
| 247 | Malik, BW | Indonesia | 2023 | Viewpoint/Commentary | Commentary | No | To analyze the legal perspectives on e-sports competition based on interpretations from the Qur'an and Hadith, as well as to examine the legal status of the professional gaming profession through the framework of Maqasid al-Sharia. | - | - | - | Correlation | All competitions with prizes (which involve illat jihad) where the prizes do not originate from third parties, such as the government, donors, or sponsors, are considered haram due to their gambling elements. This restriction on gambling practices is supported by the explanation found in Al-Qur’an, Surah Al-Maidah, verse 90. |
| 248 | Benden, J | New Zealand | 2023 | Review | Literature Review | No | To offer insight into current public attitudes, perceptions, and discourse surrounding esports in New Zealand. | - | - | - | Gateway Effect | Regarding problem gambling, some organizations view games—particularly those featuring loot boxes or other paid in-game mystery prizes—as potential gateways to gambling. They argue that these games generate similar dopamine-induced sensations akin to those experienced with slot machines and assert that certain video games are designed to cultivate a “new demographic of problem gamblers.” |
| 249 | Yusoff, AFJ | Malaysia | 2023 | Viewpoint/Commentary | Qualitative | No | This study seeks to investigate the Sharia rulings regarding the purchase of loot boxes in online games. | - | - | - | Gateway Effect | The outcome of purchasing a loot box, which is regarded as a mystery, does not align with the conditions of subject matter established in Islamic commercial law (muamalat). Consequently, this study concludes that buying loot boxes is impermissible and invalid, as it incorporates elements of gambling and fails to fulfil the essential components of a valid sale contract. |
| 250 | Pamment, J | Sweden | 2023 | Review | Literature Review | No | This study examines how gaming platforms may be utilized by foreign entities for malicious interference and information influence, emphasizing the importance of regulatory measures for gaming platforms. A key issue addressed is the need for regulations aimed at preventing addiction. | - | - | - | Converging Mechanics | In the Netherlands, loot boxes that possess real-world value have been completely banned as a form of gambling. In Belgium, several companies ignored the gaming commission’s recommendation for criminal prosecution regarding illegal loot boxes. Additionally, the German Bundestag passed a new Youth Protection Act that addresses these concerns. |
| 251 | Rozgonjuk, D | Germany | 2023 | Empirical | Cross-sectional | No | To examine the relationships among problematic online gaming, online gambling, online shopping, online pornography use, and online social networking through bivariate and multivariate network analysis within a global gaming community. | 4416 | 23.31 ± 6.72 | 6% | Correlation/Comorbidity | Problematic online gaming was found to have a positive correlation with problematic online gambling, exhibiting medium effect sizes (r = 0.299). |
| 252 | Richard, J | Canada | 2023 | Review | Systematic Review | No | To deliver a clear, concise, and thorough overview of the empirical literature concerning the relationships between externalizing problems, internalizing problems, problem gambling (PG), and problem video gaming (PVG) in youth | - | - | - | Correlation/Comorbidity | The findings from Study 1 suggested stronger evidence for an internalizing pathway leading to Problematic Video Gaming (PVG), while a combined externalizing-internalizing pathway was more significant for Problem Gambling (PG). The primary results from Study 2 indicated that PG was best classified under the externalizing factor. Although it was initially hypothesized that PVG would fit better within the internalizing factor, it was also found to align more closely with the externalizing factor, similar to PG. Studies 3 and 4 indicated that externalizing liabilities serve as a developmental predictor for both PVG and PG. However, sustained levels of externalizing issues, particularly involving behavioral under control and disinhibition during late childhood and adolescence, may be especially pertinent to PG. |
| 253 | Muela, I | Spain | 2023 | Empirical | Cross-sectional | No | This study aimed to create a scale for assessing compulsivity in two domains of potentially addictive behavior: gambling and video gaming. | 631 | 18+ | 45% | Correlation/Comorbidity | The full model demonstrated significance for both gaming and gambling. Compulsivity scores showed a slightly stronger correlation with severity scores in the gambling sample (r = 0.81) compared to the video gaming sample (r = 0.75). Compulsivity was strongly and negatively correlated with quality-of-life scores (-0.60 for the gambling sample and -0.57 for the gaming sample) and moderately and positively correlated with negative affect (0.45 for the gambling sample and 0.35 for the gaming sample). In the video gaming sample, however, severity emerged as a slightly better predictor of negative affect (r = 0.49) compared to compulsivity (r = 0.35). |
| 254 | Mohamed, MS | Sweden | 2023 | Empirical | Cohort | Yes | To investigate the prevalence and patterns of problematic gaming and gambling during the COVID-19 pandemic, as well as their association with psychiatric traits and key categories of anxiety. | 1067 | 22.9 ± 1.04 | 64.40% | Correlation/Comorbidity | During the pandemic, problematic gaming and the average time spent on games decreased, while problematic gambling increased. In Model III, after controlling for variables, social anxiety remained strongly linked to problematic gaming (p = 1.37 x 10^-7), whereas panic was the only anxiety category associated with problematic gambling (p = 0.002). The study indicated that participants who increased their gaming were more likely to report heightened feelings of loneliness, depression, and anxiety. Furthermore, while problematic gaming was associated with problem gambling in a cross-sectional context, it did not serve as a predictor for future gambling issues. |
| 255 | Carcelén-García, S | Spain | 2023 | Empirical | Cross-sectional | No | To identify the behavioral and psychographic factors that influence the likelihood of young people perceiving the risks associated with online gambling and betting, online shopping and eGames, as well as the consumption of content on social media. | 1500 | 18 to 35 | 49.10% | Correlation/Comorbidity | The likelihood of perceiving risk or danger in online betting rises with a high level of participation in online video games and online gambling. The goodness of fit for the model was confirmed using the Hosmer–Lemeshow test, which resulted in a Chi-squared value of 7.686 and a p-value of 0.465. |
| 256 | Mohammad, S | Saudi Arabia | 2023 | Review | Literature Review | No | To explain the mechanism of addiction, assess whether video game addiction qualifies as a genuine addiction, and emphasize the signs and symptoms associated with addiction. | - | - | - | Correlation/Comorbidity | The changes observed in video game addicts resemble those found in other types of addictions, including substance-related and gambling addictions. For example, frequent gamers show increased gray matter in the left ventral striatum (VS), a change that may be attributed to elevated dopamine (DA) discharge, similar to what is seen in individuals with gambling addictions. |
| 257 | Håkansson, A | Sweden | 2023 | Review | Systematic Review | No | To synthesize research findings on the prevalence and factors associated with problem gambling and problem gaming among elite athletes. | - | - | - | Correlation/Comorbidity | Five studies indicated that elite athletes have a higher risk of problem gambling compared to the general population, while one Australian study reported the opposite. Additionally, one study found that problem gaming was present in 4% of males and 1% of females over the past six months, suggesting a trend toward a significant difference. Moreover, problem gaming was significantly linked to problem gambling. |
| 258 | Cooper, DM | Switzerland | 2023 | Review | Systematic Review | No | To offer potential explanations for the connection between loot box engagement and problem gamblers or problem gamers using two prominent psychological theories: Self-Determination Theory and the Dualistic Model of Passion. | - | - | - | Correlation/Comorbidity | Need satisfaction serves as a precursor to psychological well-being, while need frustration is linked not only to psychological distress but also to symptoms of various behavioral addictions, including problem gaming and problem gambling. If need satisfaction within gambling or gaming is viewed as a way to compensate for a lack of overall need satisfaction in life, individuals may become increasingly attached to these activities. This attachment can evolve into an obsessive passion, causing individuals to lose control over their behavior towards the activity, ultimately resulting in symptoms of behavioral addiction. |
| 259 | Lakić, N | Croatia | 2023 | Empirical | Cross-sectional | No | To illustrate the playing and spending habits of Gacha players, including their motivations for enjoying these games, reasons for spending, the amount they spend, the types of purchases made, the duration of their spending, and their awareness of their spending habits. | 713 | <18 to >50 | 11.20% | Correlation/Comorbidity | This study found that while most participants did not perceive themselves as addicted to gambling, many still spent money on Gacha games, with about 10% spending over USD 300 in a single transaction. Additionally, half of the participants did not view themselves as addicted to Gacha games. The findings indicated that players who engaged with the game for a longer duration tended to spend more on it. |
| 260 | Ngetich, R | Gibraltar | 2023 | Review | Systematic Review | No | To explore the relationship between working memory (WM), an essential element of executive functions (EF), and disordered gaming and gambling | - | - | - | Converging Mechanics | The findings indicated that both gambling and gaming disorders were linked to decreased working memory (WM), but no significant relationship was found between problem gaming and WM. Notably, the difference in WM between problem gamblers and healthy controls was task-dependent, showing a significant difference only in the Ospan task and not in the digit span task. Additionally, individuals with substance use disorders exhibited significantly lower WM compared to those with gambling and gaming disorders. Previous reviews and neuroimaging studies have suggested that altered activity in the dorsolateral prefrontal cortex (DLPFC) is associated with cognitive impairments in gambling disorders, while gaming disorders were found to structurally and functionally impact key brain areas involved in WM and other executive functions, particularly the DLPFC. |
| 261 | Gomez, R | Australia | 2023 | Empirical | Cross-sectional | No | To compare four distinct models that encompass the symptoms of these addictions: a one-factor model (where all types of addiction symptoms load onto a single factor), a two-factor model (with latent factors for alcohol use and internet/gambling), a three-factor model (where alcohol use, internet gaming, and gambling symptoms load exclusively onto their respective latent factors), and a bi-factor model (where symptoms load on both their respective target latent factors and a general addiction factor). | 968 | Male 29.6 ± 8.93 Female 30.02 ± 10.39 | 32.50% | Correlation/Comorbidity | The three-factor model demonstrated a good fit, with each of its factors exhibiting strong clarity, reliability, and validity. Specifically, the correlations for the alcohol addiction factor with the internet gaming and gambling factors were 0.106 (p < 0.01) and 0.232 (p < 0.001), respectively. Furthermore, the correlation between the internet gaming and gambling factors was found to be 0.408 (p < 0.001). The omega values, which reflect the internal consistency of the factors, were impressive, measuring at 0.91 for alcohol addiction, 0.89 for internet gaming, and 0.95 for gambling. Regression analyses revealed significant and positive associations between drug addiction, distress, and the three addiction factors—alcohol, internet gaming, and gambling. This indicates that higher levels of distress are linked to greater severity in these types of addictions. |
| 262 | Floros, G | Greece | 2023 | Review | Narrative Review | No | To clarify the origins of a common exclusion criterion for gaming disorder (GD) and gambling disorder (PG)—specifically, engaging in gaming or gambling during an episode of elevated mood—by reviewing the history of its inclusion in the respective diagnoses and the supporting and opposing evidence related to GD. | - | - | - | Correlation/Comorbidity | The findings raise important concerns regarding the validity of differentiating between pathological gambling (PG) and gaming disorder (GD). Both disorders share elements of risk-taking behavior, yet current diagnostic frameworks tend to treat them similarly, primarily emphasizing the monetary risks associated with PG. However, individuals with GD also put valuable assets at risk, such as educational opportunities and employment, in their pursuit of monetary or emotional rewards through gaming. This overlap in diagnostic criteria may lead to high sensitivity in identifying these disorders but low specificity, which complicates efforts to distinguish between them effectively. As a result, a more nuanced understanding of the unique characteristics and impacts of PG and GD is necessary for accurate diagnosis and effective intervention strategies. |
| 263 | Bank, D | South Korea | 2023 | Review | Literature Review | No | To address the effect of microtransaction on self-determination | - | - | - | Converging mechanics | Freemium games advertised as free-to-play have actually incorporate in-game purchases. They require the purchase of virtual currencies which in turn are used to buy items in-game. These may affect the ability to progress with the game, game-as-a-service. Some games introduce these microtransactions until players are psychologically or financially committed. These systems are comparable to gambling systems that motivate consumer spending. |
| 264 | Duivenvoorde, B | Netherlands | 2023 | Review | Narrative Review | No | To illustrate the regulation involving digital services act in Europe | - | - | - | Converging mechanics | Influencer marketing and targeted advertisements have been on the rise. Individuals pattern of online behaviors are collected and made available to advertising companies. Those with history of competitive gaming might not comprehend why they are provided with online gambling advertisements. Safety measures of those with gambling presence virtually to receive less gambling ads is also important. Influencer marketing provide pitfalls, as such should be regulated just as any other advertising methods. |
| 265 | Jeong, DW | USA | 2023 | Review | Systematic Review | No | To analyze common keywords as themes pertaining esports | - | - | - | Converging mechanics | Apart from sports and technology, through word analysis, other thematic communities appear for esports including video games and gambling. This word community is part of the central and developed quadrant, signifying such research themes to be core concepts of esports. This theme is shared by multiple disciplines, such as psychology, medicine, social sciences, computer sciences, and arts and humanities. |
| 266 | McGrane, E | UK | 2023 | Review | Umbrella Review | No | To provide overview of gambling-related advertising leading to gambling-related harm | - | - | - | Converging mechanics | There is consistent evidence of dose-response relationship between gambling-advertisements exposure to increased risk for gambling participation and problematic gambling. These gambling advertisements have been reviewed to exist across multiple platforms, including in-game promotions. |
| 267 | Nguyen, A | Switzerland | 2023 | Review | Narrative Review | No | To provide summary of factors affecting flow or immersion in video games | - | - | - | Converging mechanics | Rewards aid in gameplay for players. More recently, in-game rewards have been packaged in loot boxes that generate revenues for game developers but present a chance-based activities akin to gambling for players. This reward mechanics may not be present in some game genres. |
| 268 | Xiao, LY | Denmark | 2023 | Empirical | Cross-sectional | No | To compare loot box disclosure between UK and China | 100 apps | - | - | Converging mechanics | 75 games in the UK AppStore games contain loot box. Of those, only 48 games disclose probabilities self-regulation and 27 games did not. This is lower than the rate in China (94.3%). |
| 269 | Brooks, GA | Canada | 2023 | Empirical | Cohort | Yes | To analyze the causal relationship between loot box and problem gambling | Baseline= 636 Follow-up= 446 | Gamblers= 22.7±1.89 Non-gamblers= 22.3±1.91 | Gamblers= 20.4% Non-gamblers= 34.2% | Gateway effect | Non-randomized purchases did not predict migration to gambling. Past year loot box expenditure (OR= 1.25, p= 0.002) and risky loot box index score (OR= 1.62, p= 0.008) predicted future problematic gambling status. |
| 270 | González-Cabrera, J | Spain | 2023 | Empirical | Cohort | Yes | To assess pattern of loot box involvement in minors and its relationship to gambling disorder longitudinally | 2213 | Time 1= 13.89 ± 1.46 Time 2= 14.20 ± 1.50 | 51.10% | Gateway effect | Loot box purchase was stable in both time (r= 0.60). Girls had higher risk ratio of partaking in online gambling at T2 after loot box involvement at T1 (RR= 3.59) than boys (RR= 1.96). Overall risk was RR= 2.28 (95% CI 1.80-2.89). Similar pattern for developing online gambling disorder in T2, overall risk is 4.00 (1.53-10.41), boys 2.32 (0.68-7.31), girls 10.74 (2.35-48.96). |
| 271 | Close, J | UK | 2023 | Empirical | Cohort | No | To investigate effect of risky loot box involvement and random item purchases compared to non-risky and non-randomized items to problem gambling, as well as the predictors and related harms | 2718 | 31.92 ± 10.2 | 46.80% | Converging mechanics | Purchasing loot box vs non-loot box content is associated with higher PGSI and IGD score. This relationship is also positive for gambling cognitions and impulsivity. The sense of 'flow' is also positively linked to risky loot box or random item purchases. However, it is positively associated to psychological distress but negatively linked to wellbeing scores. |
| 272 | Sirola, A | Finland | 2023 | Empirical | Cross-sectional | No | To examine the association of loot box purchase, indebtedness, and problem gambling | 2022 | 43.0 ± 15.24 | 45.1%% | Converging mechanics | Loneliness is associated with loot box purchasing. In turn, loot box purchasing is linked to problem gambling (b= 0.347, p< 0.001). Problem gambling is correlated to indebtedness (b= 0.507, p< 0.001). |
| 273 | Duarte, LFC | Brazil | 2023 | Review | Systematic Review | No | To summarize the evidence of loot box risk to gambling addiction | 45 studies | - | - | Converging mechanics | Multiple studies noted the link between loot box involvement and gambling addiction. Most studies were cross-sectional. Causality cannot be drawn. |
| 274 | Oelker, A | Germany | 2024 | Empirical | Cross-sectional | No | Develop an instrument measuring five behavioral addictions including gaming and gambling | 797 (gaming) and 385 (gambling) | 37.71 and 38.48 | 40.9% and 34.0% | Correlation/Comorbidity | The gaming and online gambling domain of ACSID-11 has moderate correlation at 0.674. Comparatively, gaming and online gambling through distinct methods maintains moderate correlation of 0.436-0.534. |
| 275 | Hunt, A | Switzerland | 2024 | Review | Narrative Review | No | To review aspects of substance and behavioral addiction from the perspective of evolutionary psychiatry and psychology | - | - | - | Converging mechanics | Structural characteristics of video games are engrossing, thus posing risk for problematic behaviors similar to gambling. Pathological behavior may stem from yearning of in-game rewards that appeal to the evolutionary mismatch of morality and emotion systems. |
| 276 | Saini, N | Canada | 2024 | Empirical | Cross-sectional | No | Develop a tool for assessing the addiction risk potential of various games | - | - | - | Converging mechanics | Modern games have 'Gambling-like features' that are comprised of near miss, scheduled rewards/payout intervals, in-game gambling, loot boxes, meta-game rewards, event duration, in-game purchases, daily log-in rewards, real-life rewards, no end-point, collectable items. |
| 277 | Inaguma, T | Japan | 2024 | Empirical | Case Study | No | To present a case of gaming disorder involving the use of gacha (a form of loot box in Japan) | 1 | 20s | 0% | Gateway effect | The patient initially turned to video games due to low self-esteem. The gaming behavior was propagated through loot box purchases that lead to stealing from patient's mother and threatening behavior. Loot box was attractive to the patient due to its random rewarding nature, which was alleviated using reward system and token economy. |
| 278 | Schumacher, J | Switzerland | 2024 | Viewpoint/Commentary | Narrative Review | No | To assess technological advancements, prevalence rates, and current regulations linked to the risks and harms associated with online gambling and gaming. | - | - | - | Converging Mechanics | Rapid technological advancements are continuously transforming online gaming, gambling, and related phenomena (e.g., skin betting), presenting ongoing new challenges for individuals, families, and society at large. One of the most notable developments is the convergence of gambling and gaming, where gambling activities increasingly adopt video gaming features (referred to as the ‘gamification of gambling’), while video games are progressively incorporating gambling elements into their gameplay (known as the ‘gamblification of gaming’). More efforts are required to accurately understand and explore this complex relationship. |
| 279 | Popović, L | Croatia | 2024 | Review | Literature Review | No | To examine esports through several dimensions within three primary categories: general gaming and esports, tourism and event management, and the emerging technologies and business models shaping the digital economy. | - | - | - | Converging Mechanics | eSports betting is a phenomenon that is not directly tied to traditional eSports but is also seen in conventional sports. It originated with the trading of CS: GO skins, which encountered legal challenges due to insufficient regulation. Nonetheless, the emerging eSports betting market may ultimately reflect the developments in traditional sports, especially regarding in-game gambling options. |
| 280 | Balahmar, NB | Saudi Arabia | 2024 | Empirical | Cross-sectional | No | To investigate the video game phenomenon among Saudi youth, with a focus on behaviors linked to the risks associated with gaming by examining various related factors, including spending money on in-game content (SMOIGC). | 210 | 23.99 ± 4.97 | 24.86% | Correlation/Comorbidity | The results of the bivariate correlation revealed that younger gamers were more likely to report higher levels of potential consequences and lower levels of emotional stability, while females tended to report more symptoms of anxiety and depression. Additionally, the correlation analysis indicated significant relationships between the domains of Social Media Online Gaming Consumption (SMOIGC), personality traits, and mental health. |
| 281 | Bloch, M | Poland | 2024 | Empirical | Cross-sectional | No | To identify the initial clustering patterns of problematic online gaming and gambling among a community sample of young adults who have not received psychiatric treatment at any point in their lives. | 2775 | 29.5 ± 6.3 | 51.40% | Correlation/Comorbidity | Men may be more likely to exhibit a full range of symptoms associated with both problematic online gambling and gaming. Additionally, men might be more prone to experiencing financial issues, while women may be more susceptible to losing control and developing tolerance. Overall, these findings suggest that the trajectories of problematic online gaming and gambling can differ between men and women. |
| 282 | González-Cabrera, J | Spain | 2024 | Empirical | Cross-sectional | No | To investigate the mediating role of the PU-LB in the relationship between Internet Gaming Disorder (IGD) and Online Gambling Disorder (OGD). | 523 | 11 to 30 | 0% | Gateway Effect | IGD scores were significantly correlated with both Problematic Use of Loot Boxes (PU-LB) (r=0.473, P<.001) and Online Gambling Disorder (OGD) (r=0.209, P<.001). Additionally, PU-LB showed a significant association with OGD (r=0.351, P<.001). The results from the structural equation model indicated that IGD did not have a significant direct effect on OGD (P=0.903). However, the indirect effect of IGD on OGD through PU-LB was significant (P<.001). |
| 283 | Imataka, G | Japan | 2024 | Review | Literature Review | No | To explore the suitable application of video games in terms of health and management in this research. | - | - | - | Converging Mechanics | Advancements in computer technology during the 1980s introduced virtual reels and random number generators to determine game outcomes. This development resulted in higher payouts, an increase in both winners and losers, and the incorporation of auditory effects, which has further enhanced the addictiveness of these games. |
| 284 | Suriá-Martínez, R | Spain | 2024 | Empirical | Cross-sectional | No | To comprehend the classification of risk players (non-risk players, players with issues, and pathological players) and to compare cognitive distortions among students exhibiting problematic behaviors. | 704 | 18 to 33 | 24.90% | Correlation/Comorbidity | A significant percentage of individuals are at risk of developing gambling problems, with approximately 4.7% already experiencing issues and 5.6% classified as addicted. Students with disabilities showed a higher prevalence of problematic profiles, resulting in a profile of pathological players at 11.1% and at-risk players at 5.9% (X² = 14.035, p < 0.05, Phi = 1.141). Having a disability was associated with a 2.5 times greater likelihood of becoming an at-risk player (Exp B = 0.394) compared to those without a disability, and a 4.7 times greater chance of transitioning to a problem player (Exp B = 0.214) compared to non-disabled individuals. |
| 285 | Spicer, SG | UK | 2024 | Empirical | Cohort | No | This study seeks to deepen understanding of the intricate relationships among these variables through a series of Bayesian mixed-effects multiple regressions. | 1495 | 31.16 ± 9.93 | 47.56% | Correlation/Comorbidity | The results challenge the idea of a simple, direct causal relationship between loot box engagement and symptoms of problem gambling. Instead, they suggest that this connection may be influenced by shared variance with problem video gaming and gambling-related cognitions, which simultaneously drive risky behaviors in both video games and loot boxes. Notably, the multiple regression analyses showed no evidence of a relationship with risky loot box engagement, although there was a correlation with increased spending on loot boxes. |
| 286 | Mrayati, OM | Saudi Arabia | 2024 | Empirical | Cross-sectional | No | To assess the prevalence of Internet Gaming Disorder (IGD) and loot box purchasing behaviors in video games among individuals in Saudi Arabia, while highlighting the cultural and contextual factors that shape gaming-related behaviors. | 393 | 18 to 55 | 40% | Converging mechanics | Eighty percent of the participants reported engaging with games that include loot boxes, while 14% had not, and 6% were uncertain. The logistic regression analysis highlighted a relationship between spending on loot boxes and an increased likelihood of meeting the criteria for Internet Gaming Disorder (IGD), reinforcing the association between loot box purchases and problematic gaming behaviors. This connection was further supported by a moderate positive correlation (r = 0.537, p < 0.05) between IGD scores and loot box engagement (RLI) scores among participants. This indicates that as loot box engagement scores rise, IGD scores also increase, suggesting a direct link between the extent of loot box purchases and the severity of gaming-related issues. |
| 287 | Kesuma, AE | Indonesia | 2024 | Empirical | Cross-sectional | No | To investigate the motives for gacha involvement through structural and psychological factors | 300 | 17-45 | 34.33% | Converging mechanics | Hedonic motivation and habit propagates gacha (loot box) involvement. This is influenced by the random feature that enhances the players motivation for control or manipulation. Although the intrinsic chance characteristic diminish player's control thus enabling thrill or excitement in opening the gachas. |
| 288 | Macey, J | Finland | 2024 | Review | Literature Review | No | To describe a conceptual framework of gamblification in video games | - | - | - | Converging mechanics | Authors identified five core concepts for gamblification: interaction context, ethical issues, motivations gratified, representational characteristics, and gambling mechanics. These concepts will aid in investigation of gamblified services. |
| 289 | Xiao, LY | UK | 2024 | Empirical | Cross-sectional | No | To describe loot box prevalence in China and rate of self-regulation disclosure | 100 apps | - | - | Converging mechanics | 91 out of 100 games contain loot boxes. Among games rated 12+, 90.5% contain loot boxes. About 4.4% of games did not disclose probabilities. Only 5.5% display disclosure prominently in-game. |

**References:**

1. Iain R, Brown F. Gaming, gambling, risk taking, addictions and a developmental model of a pathology of man-machine relationships. Simulation-Gaming. 1989:368.
2. Griffiths MD. Amusement machine playing in childhood and adolescence: A comparative analysis of video games and fruit machines. J Adolesc. 1991;14(1):53–73.
3. Ladouceur R, Dubé D. Brief report prevalence of pathological gambling and associated problems in individuals who visit non-gambling video arcades. Journal of Gambling Studies. 1995;11(4):361–5.
4. Fisher S, Griffiths M. current trends in slot machine gambling: research and policy issues. Journal of Gambling Studies. 1995;11(3):239–47.
5. Gupta R, Derevensky JL. The relationship between gambling and video-game playing behavior in children and adolescents. Journal of Gambling Studies. 1996;12(4):375–94.
6. Greenberg JL, Lewis SE, Dodd DK. Brief report overlapping addictions and self-esteem among college men and women. Addictive Behaviors. 1999;24(4):565–71.
7. Griffiths MD, Wood RTA. Risk factors in adolescence: the case of gambling, videogame playing, and the internet. Journal of Gambling Studies. 2000;16(2):199–225.
8. Haninger K, Thompson KM. Content and ratings of teen-rated video games. JAMA. 2004;291(7):856-65.
9. Johansson A, Gotestam KG. Problems with computer games without monetary reward : similarity to pathological gambling. Psychological Reports. 2004;95:641-650.
10. Wood RTA, Griffiths MD, Chappell D, Davies MNO. The structural characteristics of video games : a psycho-structural analysis. CyberPsychology & Behavior. 2004;7(1):1–10.
11. Wood RTA, Gupta R, Derevensky JL, Griffiths M,. Video Game Playing and Gambling in Adolescents : Common Risk Factors. Journal of Child & Adolescent Substance Abuse. 2004;14(1):77–100.
12. Messerlian C, Byrne AM, Derevensky JL. Gambling, youth and the internet: should we be concerned?. The Canadian Child and Adolescent Psychatry Review. 2004;13:1.
13. Griffiths M. Relationship between gambling and video-game playing: a response to Johansson and Gotestam. Psychological Reports. 2005;96:644–6.
14. Parker JDA, Taylor RN, Eastabrook JM, Schell SL, Wood LM. Problem gambling in adolescence: Relationships with internet misuse , gaming abuse and emotional intelligence. Personality and Individual Differences. 2008;45:174–80.
15. Griffiths MD. Digital impact, crossover technologies and gambling practices. Casino & Gaming International. 2008;4(3):37–42.
16. Delfabbro P, King D, Lambos C, Puglies S. Is video-game playing a risk factor for pathological gambling in australian adolescents?. J Gambl Stud. 2009;25:391–405.
17. Johnson TE, Dixon MR. Influencing children’s pregambling game playing via conditional discrimination training. Journal of Applied Behavior Analysis. 2009;1(1):73–81.
18. King DL, Delfabbro P, Griffiths M. The convergence of gambling and digital media : implications for gambling in young people. J Gambl Stud. 2010;26:175–87.
19. Griffiths MD. Online gambling, social responsibility and foot-in-the-door techniques. i-Gaming Business. 2010;62:100-101.
20. Griffiths MD. Gaming in social networking sites: a growing concern?. World Online Gambling Law Report. 2010;9(5):12–3.
21. Griffiths MD. Gaming convergence : further legal issues. Gaming Law Review and Economics. 2011;15(7):461–4.
22. King DL, Ejova A, Delfabbro PH. Illusory control , gambling , and video gaming : an investigation of regular gamblers and video game players. J Gambl Stud. 2012;421–35.
23. Walther B, Morgenstern M, Hanewinkel R. Co-Occurrence of addictive behaviours : personality factors related to substance use, gambling and computer gaming. Eur Addict Res. 2012;18:167–74.
24. Parke J, Wardle H, Rigbye J, Parke A. Exploring social gambling: scoping, classification and exploring social gambling: scoping, classification and evidence review. Gambling Commission: The Gambling Lab. 2012.
25. King DL, Delfabbro PH, Derevensky JL, Griffiths MD. A review of Australian classification practices for commercial video games featuring simulated gambling. International Gambling Studies. 2012;12(2):231-242.
26. Griffiths MD. The psychology of social gaming. I-Gaming Business Affiliate. 2012;26-27.
27. Critselis E, Janikian M, Paleomilitou N, Oikonomou D, Kassinopoulos M. Internet gambling is a predictive factor of Internet addictive behavior among Cypriot adolescents. Journal of Behavioral Addictions. 2013;2(4):224–30.
28. Jiménez-murcia S, Fernández-aranda F, Granero R, Chóliz M, Verde M La, Aguglia E, et al. Video game addiction in gambling disorder: clinical, psychopathological, and personality correlates. Biomed Res Int. 2014;2014:315062.
29. Calado F, Alexandre J, Griffiths MD. Mom, dad it’s only a game! perceived gambling and gaming behaviors among adolescents and young adults: an exploratory study. Int J Ment Health Addiction. 2014;12(6):772-794.
30. Gainsbury SM, Hing N, Delfabbro PH, Daniel L. A taxonomy of gambling and casino games via social media and online technologies. International Gambling Studies. 2014;14(2):196–213.
31. King DL, Delfabbro PH, Kaptsis D, Zwaans T. Adolescent simulated gambling via digital and social media: an emerging problem. Comput Human Behav. 2014;31:305–13.
32. Griffiths MD. Child and adolescent social gaming: What are the issues of concern? Education and Health. 2014;32(1):9–12.
33. Griffiths MD, King DL, Delfabbro PH. Chapter 15 of the Wiley-Blackwell handbook of disordered gambling: the technological convergence of gambling and gaming practices. John Wiley & Sons. 2014;328-346.
34. Castrén S, Grainger M, Lahti T, Alho H, Salonen AH. At-risk and problem gambling among adolescents: a convenience sample of first-year junior high school students in Finland. Substance Abuse Treatment, Prevention, and Policy. 2015;10(9):1–10.
35. Delfabbro P, King D. On finding the C in CBT : the challenges of applying gambling-related cognitive approaches to video-gaming. J Gambl Stud. 2015;31:315–29.
36. Forrest CJ, King DL, Delfabbro PH. The gambling preferences and behaviors of a community sample of australian regular video game players. J Gambl Stud. 2015;32(2):409-20.
37. Griffiths MD, King R. Are mini-games within runescape gambling or gaming?. Gaming Law Review and Economics. 2015;19(9):640–3.
38. Haskell JV. More than just skin(s) in the game : how one digital video game item is being used for unregulated gambling purposes online. Journal of High Technology Law. 2017;18(1):125-160.
39. Tárrega S, Castro-carreras L, Fernández-aranda F, Granero R, Giner-bartolomé C, Aymamí N, et al. A serious videogame as an additional therapy tool for training emotional regulation and impulsivity control in severe gambling disorder. Frontiers in Psychology. 2015;6(982):01721.
40. King DL, Gainsbury SM, Delfabbro PH, Hing N, Abarbanel B. Distinguishing between gaming and gambling activities in addiction research. Journal of Behavioral Addictions. 2015;4(4):215–20.
41. Thege BK, Hodgins DC, Wild TC. Co-occurring substance-related and behavioral addiction problems: a person-centered, lay epidemiology approach. Journal of Behavioral Addictions. 2016;5(4):614–22.
42. Larche CJ, Musielak N, Dixon MJ. The Candy Crush sweet tooth : how ‘near-misses’ in Candy Crush increase frustration , and the urge to continue gameplay. J Gambl Stud. 2016;33:599-615.
43. Kinnunen J, Alha K, Paavilainen J. Creating play money for free-to-play and gambling games. Conference: the 20^th^ International Academic Mindtrek Conference. 2016.
44. Mcbride J, Derevensky JL. Gambling and video game playing among youth. Journal of Gambling Issues. 2016;34:156-178.
45. King DL, Delfabbro PH, King DL, Delfabbro PH. Adolescents ’ perceptions of parental influences on commercial and simulated gambling activities commercial and simulated gambling activities. International Gambling Studies. 2016;16(3):1-18.
46. Brand M, Young KS, Laier C, Wölfling K, Potenza MN. Neuroscience and biobehavioral reviews integrating psychological and neurobiological considerations regarding the development and maintenance of specific internet-use disorders: an interaction of person-affect-cognition-execution (I-PACE) model. Neurosci Biobehav Rev. 2016;71:252–66.
47. Griffiths MD. Adolescent gambling and gambling-type games on social networking sites: issues, concerns, and recommendations. Aloma. 2016;33(2):31-37.
48. King DL, Delfabbro PH. Early exposure to digital simulated gambling : a review and conceptual model. Comput Human Behav. 2016;55:198–206.
49. Lopez-gonzalez H, Griffiths MD. Understanding the convergence of markets in online sports betting. IRSS. 2016;1–17.
50. Bae S, Han DH, Jung J, Nam KIC, Renshaw PF. Comparison of brain connectivity between Internet gambling disorder and Internet gaming disorder : a preliminary study. Journal of Behavioral Addictions. 2017;6(4):505-515.
51. Estévez A, Jáuregui P, Sánchez-marcos I, López-gonzález H. Attachment and emotion regulation in substance addictions and behavioral addictions. Journal of Behavioral Addictions. 2017;6(4):534–44.
52. James RJE, Tunney RJ. The relationship between gaming disorder and addiction requires a behavioral analysis; commentary on : Scholars’ open debate paper on the World Health Organization ICD-11 Gaming Disorder proposal (Aarseth et al.). Journal of Behavioral Addictions. 2017;6(3):306-309.
53. Sanders J, Williams R. Factors distinguishing problem gamblers, problem video-gamers, and dual problem gamblers/video-gamers. Abstracts of the 4^th^ ICBA: Journal Behavioral Addictions. 2017;6(1):48.
54. Gainsbury SM, Abarbanel B, Blaszczynski A. Intensity and gambling harms: Exploring breadth of gambling involvement among esports bettors. Gaming Law Review. 2017;21(8):610–5.
55. Gainsbury SM, Abarbanel B, Blaszczynski A. Game on: comparison of demographic profiles, consumption behaviors, and gambling site selection criteria of esports and sports bettors. Gaming Law Review. 2017;21(8):575–87.
56. Teichert T, Gainsbury SM, Mühlbach C. Positioning of online gambling and gaming products from a consumer perspective: A blurring of perceived boundaries. Computers in Human Behavior. 2017;75:757–65.
57. Abarbanel B, Johnson MR. Esports consumer perspectives on match-fixing: implications for gambling awareness and game integrity. International Gambling Studies. 2019;19(2):296–311.
58. Armstrong T, Rockloff M, Browne M, Li E. An exploration of how simulated gambling games may promote gambling with money. 2018;34(4):1165-1184.
59. Chen JH, Tong KK, Wu AMS, Lau JTF, Zhang MX. The comorbidity of gambling disorder among macao adult residents and the moderating role of resilience and life purpose. IJERPH. 2018;15:1–13.
60. De Pasquale C, Dinaro C, Sciacca F. Relationship of internet gaming disorder with dissociative experience in Italian university students. Ann Gen Psychiatry. 2018;17(28):1–7.
61. Hayer T, Kalke J, Meyer G, Brosowski T. Do simulated gambling activities predict gambling with real money during adolescence? empirical findings from a longitudinal study. J Gambl Stud. 2018;34(3):929-947.
62. Håkansson A, Kenttä G, Åkesdotter C. Problem gambling and gaming in elite athletes. Addict Behav Reports. 2018;8:79–84.
63. King DL. Predatory monetization schemes in video games (e.g. ‘loot boxes’) and internet gaming disorder. Addiction. 2018;113(11):1967-1969.
64. Rémond JJ, Romo L. Analysis of gambling in the media related to screen: immersion as a predictor of excessive use?. IJERPH. 2018;15(58):1-17.
65. Kircaburun K, Griffiths MD. The dark side of internet: preliminary evidence for the associations of dark personality traits with specific online activities and problematic internet use. Journal of Behavioral Addictions. 2018;7(4):993–1003.
66. Bányai F, Griffiths MD, Király O, Demetrovics Z. The psychology of esports: a systematic literature. J Gambl Stud. 2018;35(2):351–65.
67. Molde H, Holmøy B, Merkesdal AG, Torsheim T, Mentzoni RA, Hanns D, et al. Are video games a gateway to gambling? a longitudinal study based on a representative. J Gambl Stud. 2019;35(2):545-557.
68. Peter SC, Li Q, Pfund RA, Whelan JP, Meyers AW. Public stigma across addictive behaviors: casino gambling, esports gambling, and internet gaming. J Gambl Stud. 2019;35(1):247-259.
69. Sanders J, Williams R. The relationship between video gaming , gambling , and problematic levels of video gaming and gambling. J Gambl Stud. 2018;35(2):559-569.
70. Wu Y, Sescousse G, Yu H, Clark L, Li H. Cognitive distortions and gambling near- misses in Internet Gaming Disorder: a preliminary study. PLoS One. 2018;13(1):e0191110.
71. Drummond A, Sauer JD. Video game loot boxes are psychologically akin. Nat Hum Behav. 2018;2:530-532.
72. Griffiths MD. Is the buying of loot boxes in video games a form of gambling or gaming?. Gaming Law Review. 2018;52-54.
73. Schwiddessen S, Karius P. Watch your loot boxes! – recent developments and legal assessment in selected key jurisdictions from a gambling law perspective. IELR. 2018;1(1):17–43.
74. Zendle D, Cairns P. Video game loot boxes are linked to problem gambling: results of a large-scale survey. PLOSONE. 2018;13(11):e0206767.
75. King DL. Online gaming and gambling in children and adolescents – normalising gambling in cyber places. Victorian Responsible Gambling Foundation. 2018.
76. Schluter MG, Hodgins DC, Wolfe J. Can one simple questionnaire assess substance-related and behavioural addiction problems? results of a proposed new screener for community epidemiology. Addiction. 2018;113(8):1528-1537.
77. Macey J, Hamari J. Investigating relationships between video gaming, spectating esports, and gambling. Comput Human Behav. 2018;80:344–53.
78. Griffiths MD. Hot topics: gambling blocking apps, loot boxes, and ‘crypto-trading’ addiction. Online Gambling Lawyer. 2018;17(7):9-11.
79. Vadlin S, Åslund C, Nilsson KW. A longitudinal study of the individual- ­ and group- ­ level problematic gaming and associations with problem gambling among Swedish adolescents. Brain Behav. 2018;8(4):e00949.
80. Office of the eSafety Commissioner. State of play — youth and online gaming in Australia. Australian Government. 2018.
81. Brooks GA, Clark L. Associations between loot box use, problematic gaming and gambling, and gambling-related cognitions. Addict Behav. 2019;96:26–34.
82. Drummond A, Sauer JD, Hall LC. Loot box limit-setting: a potential policy to protect video game users with gambling problems?. Addiction. 2019;114(5):935-936.
83. Li W, Mills D, Nower L. The relationship of loot box purchases to problem video gaming and problem gambling. Addict Behav. 2019;97:27–34.
84. Marmet S, Studer J, Wicki M, Bertholet N, Khazaal Y, Gmel G. Unique versus shared associations between self-reported behavioral addictions and substance use disorders and mental health problems: a commonality analysis in a large sample of young Swiss men. Journal of Behavioral Addictions. 2019;8(4):664–77.
85. Wardle H. The same or different ? convergence of skin gambling and other gambling among children. J Gambl Stud. 2019;35(4):1109-1125.
86. Derevensky JL, Griffiths MD. Convergence between gambling and gaming: does the gambling and gaming industry have a responsibility in protecting the consumer?. Gaming Law Review. 2019;23(9)633–9.
87. Gainsbury SM. Gaming-gambling convergence: research, regulation, and reactions. Gaming Law Review. 2019;23(2):80–83.
88. Greer N, Rockloff M, Browne M, Hing N, King DL. Esports betting and skin gambling: a brief history review esports betting and skin gambling: a brief history. Journal of Gambling Issues. 2019;43.
89. Karlsson J, Broman N, Håkansson A. Associations between problematic gambling , gaming , and internet use : a cross-sectional population survey. Journal of Addiction. 2019;2019:1-8.
90. Liu K. A global analysis into loot boxes: is it "virtually" gambling?. Washington International Law Journal. 2019;28(3):763-799.
91. Macey J, Hamari J. eSports, skins and loot boxes: participant, practices and problematic behaviour associated with emergent forms of gambling. New Media & Society. 2019;21(1):20-41.
92. Zendle D, Bowden-Jones H. Loot boxes and the convergence of video games and gambling. The Lancet Psychiatry. 2019;6(9):724–5.
93. Scholten OJ, Drachen A, Gerard N, Hughes J, Walker JA, Deterding S, et al. Ethereum crypto-games: mechanics, prevalence and gambling similarities. CHI PLAY’19, Barcelona Spain. 2019;379–89.
94. Zendle D, Meyer R, Over H. Adolescents and loot boxes: links with problem gambling and motivations for purchase. Royal Society of Open Science. 2019;6:190049.
95. Zendle D, Cairns P. Loot boxes are again linked to problem gambling: results of a replication study. PLoS One. 2019;14(3):e0213194.
96. Mccaffrey M. The macro problem of microtransactions: the self-regulatory challenges of video game loot boxes. Bus Horiz. 2019;62(4):483–95.
97. Gambling Commission. Young people and gambling survey 2019: a survey of 11-16-year-olds in Great Britain. Gambling Commission. 2019.
98. King DL, Delfabbro P. Internet Gaming Disorder: theory, assessment, treatment, and prevention. Elsevier: Nikki Levy. 2019.
99. Abarbanel B, Phung D. Exploring gamers’ perceptions of esports betting advertising. Gaming Law Review. 2019;23(9):640–4.
100. Sweeney K, Tuttle MH, Berg MD. Esports gambling: Market structure and biases. Games and Culture. 2021 Jan;16(1):65–91.
101. Dagaev D, Stoyan E. Parimutuel betting on the eSports duels: Evidence of the reverse favourite-longshot bias. Journal of Economic Psychology. 2020 Dec;81:102305.
102. Putra MTR, Gunadi A. Legalitas sistem monetisasi lootbox dalam transaksi game online berdasarkan undang-undang nomor 11 tahun 2008 jo undang-undang nomor 19 tahun 2016. Jurnal Hukum Adigama. 2020;3(1):1480–503.
103. Rodda S. A rapid review and research gap analysis: A 2020 update [Internet]. Australia: NSW Office of Responsible Gambling; 2020 [cited 2022 Mar 2]. Available from: https://www.gambleaware.nsw.gov.au/resources-and-education/check-out-our-research/published-research/a-rapid-review-and-research-gap-analysis---a-2020-update
104. Russell AMT, Armstrong TA, Rockloff MJ, Greer NM, Hing N, Browne M. Exploring the changing landscape of gambling in childhood, adolescence and young adulthood [Internet]. Australia: CQUniversity; 2020 [cited 2022 Mar 2]. Available from: http://hdl.cqu.edu.au/10018/1333530
105. Beranuy M, Machimbarrena JM, Vega-osés MA, Carbonell X, Griffiths MD, Pontes HM, et al. Spanish validation of the internet gaming disorder scale–short form (IGDS9-SF): prevalence and relationship with online gambling and quality of life. Int J Environ Res Public Health. 2020;17(5).
106. Drummond A, Sauer JD, Ferguson CJ, Hall LC. The relationship between problem gambling, excessive gaming, psychological distress and spending on loot boxes in Aotearoa New Zealand, Australia, and the United States- a cross-national survey. PLoS One. 2020;15(3):1–16.
107. Ford M, Hakansson A. Problem gambling, associations with comorbid health conditions, substance use, and behavioural addictions: Opportunities for pathways to treatment. PLoS One. 2020;15(1):1–20.
108. González-Cabrera J, Machimbarrena JM, Beranuy M, Pérez-Rodríguez P, Fernández-González L, Calvete E. Design and measurement properties of the online gambling disorder questionnaire (OGD-Q) in Spanish adolescents. J Clin Med. 2020;9(1):1–15.
109. Kim HS, Hodgins DC, Kim B, Wild TC. Transdiagnostic or disorder specific? Indicators of substance and behavioral addictions nominated by people with lived experience. J Clin Med. 2020;9(2).
110. King A, Wong-Padoongpatt G, Barrita A, Phung DT, Tong T. Risk factors of problem gaming and gambling in us emerging adult non-students: the role of loot boxes, microtransactions, and risk-taking. Issues Ment Health Nurs. 2020;41(12):1063–75.
111. King DL, Russell AMT, Delfabbro PH, Polisena D. Fortnite microtransaction spending was associated with peers’ purchasing behaviors but not gaming disorder symptoms. Addict Behav. 2020;104:106311.
112. King DL, Delfabbro PH. The convergence of gambling and monetised gaming activities. Curr Opin Behav Sci. 2020;31:32–6.
113. Kotyuk E, Magi A, Eisinger A, Király O, Vereczkei A, Barta C, et al. Co-occurrences of substance use and other potentially addictive behaviors: epidemiological results from the psychological and genetic factors of the addictive behaviors (PGA) study. J Behav Addict. 2020;9(2):272–88.
114. McCaffrey M. A cautious approach to public policy and loot box regulation. Addict Behav. 2020;102:1-3.
115. Richard J, Fletcher E, Boutin S, Derevensky J, Temcheff C. Conduct problems and depressive symptoms in association with problem gambling and gaming: a systematic review. J Behav Addict. 2020;9(3):497–533.
116. Shi J, Colder Carras M, Potenza MN, Turner NE. A Perspective on age restrictions and other harm reduction approaches targeting youth online gambling, considering convergences of gambling and videogaming. Front Psychiatry. 2020;11:601712.
117. Zendle D, Meyer R, Cairns P, Waters S, Ballou N. The prevalence of loot boxes in mobile and desktop games. Addiction. 2020;115(9):1768–72.
118. Prati AM. Video games in the twenty-first century: parallels between loot boxes and gambling create an urgent need for regulatory action. Vanderbilt J Entertain Technol Law. 2020;22(1):215–50.
119. Drummond A, Sauer JD, Hall LC, Zendle D, Loudon MR. Why loot boxes could be regulated as gambling?. Nat Hum Behav. 2020;4(10):986–8.
120. Gainsbury SM, Philander KS, Grattan G. Skill gambling machines and electronic gaming machines: participation, erroneous beliefs, and understanding of outcomes. Int Gambl Stud. 2020;20(3):500–514.
121. Kalkan B, Bhat CS. Relationships of problematic internet use, online gaming, and online gambling with depression and quality of life among college students. Int J Contemp Educ Res. 2020 7(1):18-28.
122. Macey J, Hamari J. GamCog: a measurement instrument for miscognitions related to gamblification, gambling, and video gaming. Psychol Addict Behav. 2020;34(1):242-256.
123. Serada A. Why is cryptokitties (not) gambling?. ACM Int Conf Proceeding Ser. 2020;18–21.
124. Wardle H, Petrovskaya E, Zendle D. Defining the esports bettor: evidence from an online panel survey of emerging adults. Int Gambl Stud. 2020;20(3):487–499.
125. Zendle D, Cairns P, Barnett H, McCall C. Paying for loot boxes is linked to problem gambling, regardless of specific features like cash-out and pay-to-win. Comput Human Behav. 2020;102:181–91.
126. DeCamp W. Loot boxes and gambling: similarities and dissimilarities in risk and protective factors. J Gambl Stud. 2020;37(1):189–201.
127. Delfabbro P, King DL. Gaming-gambling convergence: evaluating evidence for the ‘gateway’ hypothesis. Int Gambl Stud. 2020;20(3):380–92.
128. Biegun J, Edgerton JD, Roberts LW. Measuring problem online video gaming and its association with problem gambling and suspected motivational, mental health, and behavioral risk factors in a sample of university students. games cult. 2020;16(4):434–56.
129. Labrador FJ, Bernaldo-de-Quirós M, Sánchez-Iglesias I, Labrador M, Vallejo-Achón M, Fernández-Arias I, et al. Advertising games of chance in adolescents and young adults in Spain. J Gambl Stud. 2020;37(3):765–78.
130. Kristiansen S, Severin MC. Loot box engagement and problem gambling among adolescent gamers: Findings from a national survey. Addict Behav. 2020;103):106254.
131. Kim HS, King DL. Gambling-gaming convergence: new developments and future directions. Int Gambl Stud. 2020;20(3):373–379.
132. Li W, Mills D, Nower L. Validation of the problematic social casino gaming scale. Int Gambl Stud. 2020;20(3):436–4511.
133. Gainsbury SM, Philander KS, Blaszczynski A. A qualitative study of participant experience with skill gaming machines in comparison to electronic gaming machines. Int Gambl Stud. 2020;20(3):452–65.
134. Brosowski T, Turowski T, Hayer T. Simulated gambling consumption mediation model (SGCMM): disentangling convergence with parallel mediation models. Int Gambl Stud. 2020;20(3):466–486.
135. Mills DJ, Marchica L, Keough MT, Derevensky JL. Exploring differences in substance use among emerging adults at-risk for problem gambling, and/or problem video gaming. Int Gambl Stud. 2020;20(3):539–55.
136. Abarbanel B, Johnson MR. Gambling engagement mechanisms in Twitch live streaming. Int Gambl Stud. 2020;20(3):393–413.
137. Macey J, Kinnunen J. The convergence of play: interrelations of social casino gaming, gambling, and digital gaming in Finland. Int Gambl Stud. 2020;20(3):414-435.
138. Zendle D. Beyond loot boxes: a variety of gambling-like practices in video games are linked to both problem gambling and disordered gaming. PeerJ. 2020;8:e9466.
139. von Meduna M, Steinmetz F, Ante L, Reynolds J, Fiedler I. Loot boxes are gambling-like elements in video games with harmful potential: results from a large-scale population survey. Technol Soc. 2020;63:101395.
140. Zanescu A, French M, Lajeunesse M. Betting on DOTA 2’s battle pass: gamblification and productivity in play. New Media Soc. 2021;23(10):2882–901.
141. Laato S. Is simulating casino environments in video games worse than gambling with loot boxes? The case of the removed Pokémon game corner. CEUR Workshop Proc. 2020;2737:99–109.
142. Greer N. Gambling and video games: are esports betting and skin gambling associated with greater gambling involvement and harm?. Victorian Responsible Gambling Foundation. 2020;1-50.
143. Rockloff M, Russell AMT, Greer N, Lolé L, Hing N, Browne M. Loot Boxes: Are they grooming youth for gambling? [Internet]. Australia: CQ University; 2020 [cited 2024 Sep 20]. Available from: http://hdl.cqu.edu.au/10018/1331666
144. Castren S, Jarvinen-Tassopoulos J, Raitasalo K. Money used in gaming is associated with problem gambling: Results of the ESPAD 2019 Finland. J Behav Addict. 2021;10(4):932–40.
145. Hall LC, Drummond A, Sauer JD, Ferguson CJ. Effects of self-isolation and quarantine on loot box spending and excessive gaming-results of a natural experiment. PeerJ. 2021;9:1–16.
146. Hing N, Russell AMT, Bryden GM, Newall P, King DL, Rockloff M, et al. Skin gambling predicts problematic gambling amongst adolescents when controlling for monetary gambling. J Behav Addict. 2021;10(4):920–31.
147. Lelonek-Kuleta B, Bartczuk RP. Online gambling activity, pay-to-win payments, motivation to gamble and coping strategies as predictors of gambling disorder among e-sports bettors. J Gambl Stud. 2021;37(4):1079–98.
148. Luquiens A, von Hammerstein C, Benyamina A, Perney P. Burden and help-seeking behaviors linked to problem gambling and gaming: observational quantitative and qualitative analysis. JMIR Ment Heal. 2021;8(11):1–10.
149. Steinmetz F, Fiedler I, von Meduna M, Ante L. Pay-to-win gaming and its interrelation with gambling: findings from a representative population sample. Journal of Gambling Studies. J Gambl Stud; 2021;38(3):785-816.
150. Ayala-Rojas RE, Granero R, Mora-Maltas B, Rivas S, Fernández-Aranda F, Gómez-Peña M, et al. Factors related to the dual condition of gambling and gaming disorders: a path analysis model. J Psychiatr Res. 2021;145:148–58.
151. Close J, Spicer SG, Nicklin LL, Lloyd J, Lloyd H. Loot box engagement: relationships with educational attainment, employment status and earnings in a cohort of 16000 United Kingdom gamers. Addiction. 2021;117(8):2338–45.
152. Greer N, Rockloff MJ, Russell AMT, Lole L. Are esports bettors a new generation of harmed gamblers? a comparison with sports bettors on gambling involvement, problems, and harm. J Behav Addict. 2021;10(3):435-446.
153. Marchica L, Richard J, Mills D, Ivoska W, Derevensky J. Between two worlds: exploring esports betting in relation to problem gambling, gaming, and mental health problems. J Behav Addict. 2021;10(3):447-455.
154. Macey J, Abarbanel B, Hamari J. What predicts esports betting? a study on consumption of video games, esports, gambling and demographic factors. New Media Soc. 2021;23(6):1481–505.
155. Mason L. Video gaming – taking a gamble with young people’s health and wellbeing? Perspect Public Health. 2021;141(1):5–6.
156. Perreault G, Daniel E, Tham SM. The gamification of gambling: A case study of the mobile game Final Fantasy Brave Exvius journalistic role conception and technological innovation view project videogames and strategic communication view project. Game Stud. 2021;21(2):1–17.
157. Wardle H. Games without frontiers?: socio-historical perspectives at the gaming/gambling intersection (part of leisure studies in a global era). Palgrave Macmillan. 2021.
158. Duffy L. Gen bet : a plain English summary of research into gambling and young people. Victorian Responsible Gambling Foundation. 2021;1-19.
159. Nicklin LL, Spicer SG, Close J, Parke J, Smith O, Raymen T, et al. “It’s the attraction of winning that draws you in”—a qualitative investigation of reasons and facilitators for videogame loot box engagement in UK gamers. J Clin Med. 2021;10(10):2103.
160. Rockloff M, Russell AMT, Greer N, Lole L, Hing N, Browne M. Young people who purchase loot boxes are more likely to have gambling problems: an online survey of adolescents and young adults living in NSW Australia. J Behav Addict. 2021;10(1):35–41.
161. Kolandai-Matchett K, Wenden Abbott M. Gaming-gambling convergence: trends, emerging risks, and legislative responses. Int J Ment Health Addict. 2021;20(4):2024–56.
162. Carey PAK, Delfabbro P, King D. An evaluation of gaming-related harms in relation to gaming disorder and loot box involvement. Int J Ment Health Addict. 2021;7:1-16.
163. Spicer SG, Nicklin LL, Uther M, Lloyd J, Lloyd H, Close J. Loot boxes, problem gambling and problem video gaming: a systematic review and meta-synthesis. New Media Soc. 2021;24(4):1001–22.
164. Stark S, Reynolds J, Wiebe J. Gambling and gaming in an ontario sample of youth and parents. J Gambl Issues. 2021;46:4–24.
165. Close J, Spicer SG, Nicklin LL, Uther M, Lloyd J, Lloyd H. Secondary analysis of loot box data: are high-spending “whales” wealthy gamers or problem gamblers? Addict Behav. 2021;117:106851.
166. Palmeira M. The interplay of micro-transaction type and amount of playing in video game evaluations. Comput Human Behav. 2021;115:106609.
167. Brock T, Johnson M. The gamblification of digital games. J Consum Cult. 2021;21(1):3–13.
168. Derrington S, Star S, Kelly SJ. The case for uniform loot box regulation: a new classification typology and reform agenda. J Gambl Issues. 2021;46:302–32.
169. Garea SS, Drummond A, Sauer JD, Hall LC, Williams MN. Meta-analysis of the relationship between problem gambling, excessive gaming and loot box spending. Int Gambl Stud. 2021;21(3):460–79.
170. Whitson J, French M. Productive play: The shift from responsible consumption to responsible production. J Consum Cult. 2021;21(1):14–33.
171. Thorhauge AM, Nielsen RKL. Epic, Steam, and the role of skin-betting in game (platform) economies. J Consum Cult. 2021;21(1):52–67.
172. Joseph D. Battle pass capitalism. J Consum Cult. 2021;21(1):68–83.
173. Jarrett J. Gaming the gift: The affective economy of League of Legends ‘fair’ free-to-play model. J Consum Cult. 2021;21(1):102–19.
174. Macey J, Hamari J, Sjöblom M, Törhönen M. Relationships between the consumption of gamblified media and associated gambling activities in a sample of esports fans. CEUR Workshop Proc. 2021;2883:120–9.
175. Denoo M, Bibert N, Zaman B. Disentangling the Motivational Pathways of Recreational Esports Gamblers: A Laddering Study. In: Proceedings of the 2021 CHI Conference on Human Factors in Computing Systems. Yokohama Japan: ACM; 2021. p. 1–15.
176. Freitas BDA, Contreras-Espinosa RS, Correia PÁP. Model of the Threats that Disreputable Behavior Present to Esports Sponsors. CMR. 2021;17(1):27–64.
177. Rossi R, Nairn A, Smith J, Inskip C. “Get a £10 Free Bet Every Week!”—Gambling Advertising on Twitter: Volume, Content, Followers, Engagement, and Regulatory Compliance. Journal of Public Policy & Marketing. 2021;40(4):487–504.
178. Ide S, Nakanishi M, Yamasaki S, Ikeda K, Ando S, Hiraiwa-Hasegawa M, et al. Adolescent Problem Gaming and Loot Box Purchasing in Video Games: Cross-sectional Observational Study Using Population-Based Cohort Data. JMIR Serious Games. 2021;9(1):e23886. 1.
179. Xiao LY. Regulating loot boxes as gambling? Towards a combined legal and self-regulatory consumer protection approach. IELR. 2021;4(1):27–47.
180. Xiao LY. Conceptualising the Loot Box Transaction as a Gamble Between the Purchasing Player and the Video Game Company. Int J Ment Health Addiction. 2021;19(6):2355–7.
181. Hing N, Dittman CK, Russell AMT, King DL, Rockloff M, Browne M, et al. Adolescents Who Play and Spend Money in Simulated Gambling Games Are at Heightened Risk of Gambling Problems. Int J Environ Res Public Health. 2022 Aug 26;19(17).
182. Lischer S, Jeannot E, Brülisauer L, Weber N, Khazaal Y, Bendahan S, et al. Response to the Regulation of Video Games under the Youth Media Protection Act: A Public Health Perspective. Int J Environ Res Public Health. 2022;19(15).
183. André F, Einarsson I, Dahlström E, Niklasson K, Håkansson A, Claesdotter-Knutsson E. Cognitive behavioral treatment for disordered gaming and problem gambling in adolescents: a pilot feasibility study. Ups J Med Sci. 2022;127.
184. Wardle H, Tipping S. The relationship between problematic gambling severity and engagement with gambling products: Longitudinal analysis of the Emerging Adults Gambling Survey. Addiction. 2023;118(6):1127–39.
185. Drummond A, Hall LC, Sauer JD. Surprisingly high prevalence rates of severe psychological distress among consumers who purchase loot boxes in video games. Sci Rep. 2022;12(1):16128.
186. King A, Wong-Padoongpatt G. Do Gamers Play for Money? A Moderated Mediation of Gaming Motives, Relative Deprivation, and Upward Mobility. Int J Environ Res Public Health. 2022;19(22).
187. Jouhki H, Savolainen I, Sirola A, Oksanen A. Escapism and Excessive Online Behaviors: A Three-Wave Longitudinal Study in Finland during the COVID-19 Pandemic. IJERPH. 2022;19(19):12491.
188. Costes JM, Bonnaire C. Spending Money in Free-to-Play Games: Sociodemographic Characteristics, Motives, Impulsivity and Internet Gaming Disorder Specificities. Int J Environ Res Public Health. 2022;19(23).
189. Szerman N, Basurte-Villamor I, Vega P, Mesías B, Martínez-Raga J, Ferre F, et al. Is there such a thing as gambling dual disorder? Preliminary evidence and clinical profiles. Eur Neuropsychopharmacol. 2023;66:78–91.
190. D’Amico NJ, Drummond A, de Salas K, Lewis I, Waugh C, Bannister B, et al. No effect of short term exposure to gambling like reward systems on post game risk taking. Sci Rep. 2022;12(1):16751.
191. Baggio S, Starcevic V, Billieux J, King DL, Gainsbury SM, Eslick GD, et al. Testing the spectrum hypothesis of problematic online behaviors: A network analysis approach. Addict Behav. 2022;135:107451.
192. Burleigh TL, Griffiths MD, Sumich A, Wang GY, Stavropoulos V, Kannis-Dymand L, et al. Co-Occurrence of Gaming Disorder and Other Potentially Addictive Behaviours between Australia, New Zealand, and the United Kingdom. Int J Environ Res Public Health. 2022;19(23). 1.
193. Budiman R, Romadini NA, Herwandi Aziz MA, Pratama AG. The Impact of Online Gambling Among Indonesian Teens and Technology. IAIC Transactions on Sustainable Digital Innovation. 2022;3(2):162–7.
194. Lubis AA, Saleh S, Marsa YJ. The phenomenon of online gambling under the guise of online games among college student. J Hum Soc Stud. 2022;6(3):363–7.
195. Dagwi N. Perceived Images of Esports and their Effects On Its Content Consumption [Internet] [Thesis]. [South Korea]: Seoul National University; 2022 [cited 2024 Sep 20]. Available from: https://s-space.snu.ac.kr/handle/10371/188149
196. Balhara YPS, Singh S. Fantasy Sports: Current Status, Implications and Way Forward. Postgraduate Journal of Pediatrics and Adolescent Medicine. 2022;1(2):16–20.
197. Declerck P, Feci N. Mapping and analysis of the current regulatory framework on gambling(-like) elements in video games : a report in the framework of the ‘Gam(e)(a)ble’ research project’ [Internet]. Belgium: Universiteit Gent; 2022 [cited 2024 Sep 20]. Report No.: 8768539. Available from: http://hdl.handle.net/1854/LU-8768539
198. Riatti P, Thiel A. The societal impact of electronic sport: a scoping review. Ger J Exerc Sport Res. 2022;52(3):433–46.
199. Daglis T. The excessive gaming and gambling during COVID-19. Journal of Economic Studies. 2022;49(5):888–901.
200. Lemmens JS. Play or pay to win: Loot boxes and gaming disorder in FIFA ultimate team. Telematics and Informatics Reports. 2022;8:100023.
201. Ramella-Zampa B, Carbone GA, Panno A, Santos M, Imperatori C, Budde H, et al. Understanding and conceptualizing eSports among behavioural addictions: A brief overview of the literature. Emerging Trends in Drugs, Addictions, and Health. 2022;2:100048.
202. Tavares R, Sousa JP, Maganinho B, Gomes JP. Gamers’ Reaction to the Use of NFT in AAA Video Games. Procedia Computer Science. 2022;219:606–13.
203. Garrett EP, Sauer JD, Drummond A, Lowe-Calverley E. Problem gambling and income as predictors of loot box spending. Intl Gambl Stud. 2022;22(3):432–43.
204. Cena L, Rota M, Calza S, Trainini A, Zecca S, Zappa SB, et al. Prevalence and types of video gaming and gambling activities among adolescent public school students: findings from a cross-sectional study in Italy. Ital J Pediatr. 2022;48(1):4–7.
205. Gomez R, Stavropoulos V, Brown T, Griffiths MD. Factor structure of ten psychoactive substance addictions and behavioural addictions: common psychoactive substance and behavioural addictions. Psychiatry Res. 2022;313:114605.
206. Greer N, Hing N, Rockloff M, Browne M, King DL. Motivations for esports betting and skin gambling and their association with gambling frequency, problems, and harm. J Gambl Stud. 2022.
207. Greer N, Rockloff M, Hing N, Browne M, King DL. Skin gambling contributes to gambling problems and harm after controlling for other forms of traditional gambling. J Gambl Stud. 2022.
208. Hing N, Lole L, Russell AMT, Rockloff M, King DL, Browne M, et al. Adolescent betting on esports using cash and skins: Links with gaming, monetary gambling, and problematic gambling. PLoS One. 2022;17:1–16.
209. Hing N, Rockloff M, Russell AMT, Browne M, Newall P, Greer N, et al. Loot box purchasing is linked to problem gambling in adolescents when controlling for monetary gambling participation. J Behav Addict. 2022;11(2):396-405.
210. Macía L, Estévez A, Jáuregui P. Gambling: Exploring the role of gambling motives, attachment and addictive behaviours among adolescents and young women. J Gambl Stud. 2022.
211. Müller SM, Wegmann E, Oelker A, Stark R, Müller A, Montag C, et al. Assessment of criteria for specific internet-use disorders (ACSID-11): introduction of a new screening instrument capturing ICD-11 criteria for gaming disorder and other potential internet-use disorders. J Behav Addict. 2022;11:427–50.
212. Puiras E, Oliver C, Cummings S, Sheinin M, Mazmanian D. Motives to engage with or refrain from gambling and loot box content: an exploratory qualitative investigation. J Gambl Stud. 2022;
213. Savolainen I, Vuorinen I, Sirola A, Oksanen A. Gambling and gaming during COVID-19: The role of mental health and social motives in gambling and gaming problems. Compr Psychiatry. 2022;117:152331.
214. Sidloski B, Brooks GA, Zhang K, Clark L. Exploring the association between loot boxes and problem gambling: are video gamers referring to loot boxes when they complete gambling screening tools?. Addict Behav. 2022;131:107318.
215. Spicer SG, Fullwood C, Close J, Nicklin LL, Lloyd J, Lloyd H. Loot boxes and problem gambling: investigating the “gateway hypothesis.” Addict Behav. 2022;131:107327.
216. Vuorinen I, Savolainen I, Hagfors H, Oksanen A. Basic psychological needs in gambling and gaming problems. Addict Behav Reports. 2022;16:100445.
217. Zarate D, Ball M, Montag C, Prokofieva M, Stavropoulos V. Unravelling the web of addictions: A network analysis approach. Addict Behav Reports. 2022;15:100406.
218. Ciccarelli M, Cosenza M, Nigro G, Griffiths M, D’Olimpio F. Gaming and gambling in adolescence: the role of personality, reflective functioning, time perspective and dissociation. Int Gambl Stud. 2022;22(1):161–79.
219. Close J, Spicer SG, Nicklin LL, Lloyd J, Whalley B, Lloyd H. Gambling and Gaming in the United Kingdom during the COVID-19 Lockdown. Covid. 2022;2(2):87–101.
220. Woods O. The affective embeddings of gacha games: aesthetic assemblages and the mediated expression of the self. New Media Soc. 2022;1-16.
221. Montiel I, Basterra-González A, Machimbarrena JM, Ortega-Barón J, González-Cabrera J. Loot box engagement: A scoping review of primary studies on prevalence and association with problematic gaming and gambling. PLoS One. 2022;17:1–23.
222. Raneri PC, Montag C, Rozgonjuk D, Satel J, Pontes HM. The role of microtransactions in internet gaming disorder and gambling disorder: a preregistered systematic review. Addict Behav Reports. 2022;15:100415.
223. Gibson E, Griffiths MD, Calado F, Harris A. The relationship between videogame micro-transactions and problem gaming and gambling: A systematic review. Comput Human Behav. 2022;131:107219.
224. Woods O. The economy of time, the rationalisation of resources: discipline, desire and deferred value in the playing of gacha games. Games Cult. 2022;0(0):1–18.
225. Hing N, Browne, M, Rockloff, M, Lole, L, Russell, AMT. Gamblification: risks of digital gambling games to adolescents. The Lancet Child & Adolescent Health. 2022;6(6):357-359.
226. Xiao LY, Henderson LL, Newall PWS. Loot boxes are more prevalent in United Kingdom video games than previously considered: updating Zendle *et al* . (2020). Addiction. 2022;117(9):2553–5.
227. Amadieu T. Addictive Technologies? The Moral and Normative Dynamics Shaping the Chinese Gaming Culture. In: Chrétien-Ichikawa S, Pawlik K, editors. Creative Industries and Digital Transformation in China [Internet]. Singapore: Springer Nature Singapore; 2022 [cited 2024 Sep 20]. p. 59–82. Available from: https://link.springer.com/10.1007/978-981-19-3049-2_4
228. Etchells PJ, Morgan AL, Quintana DS. Loot box spending is associated with problem gambling but not mental wellbeing. R Soc open sci. 2022;9(8):220111.
229. Aagaard J, Knudsen MEC, Bækgaard P, Doherty K. A Game of Dark Patterns: Designing Healthy, Highly-Engaging Mobile Games. In: CHI Conference on Human Factors in Computing Systems Extended Abstracts [Internet]. New Orleans LA USA: ACM; 2022 [cited 2024 Sep 20]. p. 1–8. Available from: <https://dl.acm.org/doi/10.1145/3491101.3519837>
230. Primi C, Sanson F, Vecchiato M, Serra E, Donati MA. Loot boxes use, video gaming, and gambling in adolescents: Results from a path analysis before and during COVID-19-pandemic-related lockdown in Italy. Front Psychol. 2022;13:1009129.
231. Clark L, Zack M. Engineered highs: Reward variability and frequency as potential prerequisites of behavioural addiction. Addict Behav. 2023;140:107626.
232. Clark L, Zack M. Engineered highs: Reward variability and frequency as potential prerequisites of behavioural addiction. Addict Behav. 2023;140:107626.
233. Seffah KD, Salib K, Dardari L, Taha M, Dahat P, Toriola S, et al. Health Benefits of Esports: A Systematic Review Comparing the Cardiovascular and Mental Health Impacts of Esports. Cureus. 2023;15(6):e40705.
234. Sanmartín FJ, Velasco J, Cuadrado F, Gálvez-Lara M, De Larriva V, Moriana JA. Loot boxes use as a new form of gambling within video games. Adicciones. 2023;35(4):407–20.
235. Kim HS, Leslie RD, Stewart SH, King DL, Demetrovics Z, Andrade ALM, et al. A scoping review of the association between loot boxes, esports, skin betting, and token wagering with gambling and video gaming behaviors. J Behav Addict. 2023;12(2):309–51.
236. Xiao LY, Fraser TC, Newall PWS. Opening Pandora’s Loot Box: Weak Links Between Gambling and Loot Box Expenditure in China, and Player Opinions on Probability Disclosures and Pity-Timers. J Gambl Stud. 2023;39(2):645–68.
237. Russell AMT, Hing N, Newall P, Greer N, Dittman CK, Thorne H, et al. Order of first-play in simulated versus monetary gambling. J Behav Addict. 2023;12(4):992–1005.
238. Mangat HS, Griffiths MD, Yu SM, Felvinczi K, Ngetich RK, Demetrovics Z, et al. Understanding Esports-related Betting and Gambling: A Systematic Review of the Literature. J Gambl Stud. 2023;40(2):893–914.
239. Wieczorek Ł, Bujalski M, Dąbrowska K. “I Can Tell You It’s a Bit of a Gamble”: A Qualitative Analysis of How People Who Engage in Gaming and Gambling Understand a Link Between These Two Behaviours. J Gambl Stud. 2023;40(2):859–71.
240. DeCamp W, Daly K. Loot box consumption by adolescents pre- and post- pandemic lockdown. PeerJ. 2023;11:e15287.
241. Yüce A, Gökce Yüce S, Katırcı H, Aydoğdu V, Chiu W, Griffiths MD. The Effect of the COVID-19 Pandemic on Sports Betting Tipsters as Professional Bettors: A Qualitative Interview Study. Sustainability. 2023;15(9):7729.
242. Király O, Koncz P, Griffiths MD, Demetrovics Z. Gaming disorder: A summary of its characteristics and aetiology. Compr Psychiatry. 2023;122:152376.
243. Cole JD. Understanding School Counselors’ Perceptions of Esports and Igaming as a Career Choice [Doctoral]. [USA]: Oregon State University; 2023 [cited 2024 Sep 20]. Available from: https://ir.library.oregonstate.edu/concern/graduate_thesis_or_dissertations/j9602803m
244. Bujňáková E. Consumer Behavior of League of Legends Players [Internet] [Bachelor]. [Finland]: Satakunta University of Applied Sciences; 2023 [cited 2024 Sep 20]. Available from: <http://www.theseus.fi/handle/10024/812974>
245. Greer N. Experiences with esports betting and skin gambling: Exposure, access, motivations and impacts [Internet] [Thesis]. [Australia]: CQUniversity; 2023 [cited 2024 Sep 20]. Available from: <https://acquire.cqu.edu.au/articles/thesis/Experiences_with_esports_betting_and_skin_gambling_Exposure_access_motivations_and_impacts/23897322/1>
246. Liniers BV. Use of Telemetry Data and Emotion Analysis to complement the analysis of Problematic Gaming Behaviors [Internet] [Masters]. [Portugal]: Universidade de Lisboa; 2023 [cited 2024 Sep 20]. Available from: <https://repositorio.ul.pt/handle/10451/63287?locale=en>
247. Malik BW, Harisah H. An Islamic perspective on e-sport competition. Islamuna: Jurnal Studi Islam. 2024;11(1):165–83.
248. Benden J. New Zealand attitudes towards the emerging sport of Esports: Content analysis of New Zealand public discourse on Esports : A thesis submitted in partial fulfilment of the requirements for the Degree of Master of Applied Science at Lincoln University [Internet] [Masters]. [New Zealand]: Lincoln University; 2023 [cited 2024 Sep 20]. Available from: <https://researcharchive.lincoln.ac.nz/entities/publication/c2ef54d8-25af-4e35-9ffc-b3eca28a2817>
249. Yusoff AFJ, Razak IA. Sharia Issues on Loot Boxes in Online Games and Esport. Samarah: Jurnal Hukum Keluarga dan Hukum Islam. 2023;7(1):341–56.
250. Pamment J, Falkheimer J, Isaksson E. Malign foreign interference and information influence on video game platforms: Understanding the adversarial playbook [Internet]. Sweden: Psychological Defence Agency; 2023 [cited 2024 Sep 20]. (MPF Report). Available from: <https://mpf.se/psychological-defence-agency/about-us/news/2023/2023-10-09-malign-foreign-interference-and-information-influence-on-video-game-platforms-understanding-the-adversarial-playbook>
251. Rozgonjuk D, Schivinski B, Pontes HM, Montag C. Problematic Online Behaviors Among Gamers: the Links Between Problematic Gaming, Gambling, Shopping, Pornography Use, and Social Networking. Int J Ment Health Addiction. 2023;21(1):240–57.
252. Richard J. Understanding the development of problem gambling and gaming: the role of externalizing and Internalizing problems from childhood to emerging adulthood [Internet] [Doctoral]. [Canada]: McGill University; 2023 [cited 2024 Sep 20]. Available from: <https://escholarship.mcgill.ca/concern/theses/nz806558w>
253. Muela I, Navas JF, Barrada JR, López-Guerrero J, Rivero FJ, Brevers D, et al. Operationalization and measurement of compulsivity across video gaming and gambling behavioral domains. BMC Psychol. 2023;11(1):407.
254. Mohamed MS, Rukh G, Schiöth HB, Vadlin S, Olofsdotter S, Åslund C, et al. Worsened Anxiety and Loneliness Influenced Gaming and Gambling during the COVID-19 Pandemic. JCM. 2023;12(1):249. 1.
255. Carcelén-García S, Díaz-Bustamante Ventisca M, Galmes-Cerezo M. Young People’s Perception of the Danger of Risky Online Activities: Behaviours, Emotions and Attitudes Associated with Their Digital Vulnerability. Social Sciences. 2023;12(3):164.
256. Mohammad S, Jan RA, Alsaedi SL. Symptoms, Mechanisms, and Treatments of Video Game Addiction. Cureus. 2023;15(3):e36957.
257. Håkansson A, Durand-Bush N, Kenttä G. Problem Gambling and Problem Gaming in Elite Athletes: a Literature Review. Int J Ment Health Addiction. 2023;21(3):1837–53.
258. Cooper DM. Needs, Passions and Loot Boxes - Exploring Reasons for Problem Behaviour in Relation to Loot Box Engagement [Internet]. Switzerland: arXiv; 2023. Available from: <https://arxiv.org/abs/2307.04549>
259. Lakić N, Bernik A, Čep A. Addiction and Spending in Gacha Games. Information. 2023;14(7):399.
260. Ngetich R, Burleigh TL, Czakó A, Vékony T, Németh D, Demetrovics Z. Working memory performance in disordered gambling and gaming: A systematic review. Compr Psychiatry. 2023;126:152408.
261. Gomez R, Stavropoulos V, Brown T, Watson S. Factor Structure of the Symptoms of Alcohol Use, Gaming, and Gambling Addictions. Int J Ment Health Addiction. 2023;21(5):3345–61.
262. Floros G, Mylona I. Bipolar Disorder and Gaming Disorder—Compatible or Incompatible Diagnoses? J Clin Med. 2023;12(19):6251. 1.
263. Bank D. Problematic monetization in mobile games in the context of the human right to economic self-determination. Computers in Human Behavior. 2023;149:107958.
264. Duivenvoorde B, Goanta C. The regulation of digital advertising under the DSA: A critical assessment. Computer Law & Security Review. 2023;51:105870.
265. Jeong D, Youk S. Refining esports: A quantitative cartography of esports literature. Entertainment Computing. 2023;47:100597.
266. McGrane E, Wardle H, Clowes M, Blank L, Pryce R, Field M, et al. What is the evidence that advertising policies could have an impact on gambling-related harms? A systematic umbrella review of the literature. Public Health. 2023;215:124–30.
267. Nguyen A, Bavelier D. Play in video games. Neuroscience & Biobehavioral Reviews. 2023;153:105386.
268. Xiao LY, Henderson LL, Newall PWS. What are the odds? Poor compliance with UK loot box probability disclosure industry self-regulation. Grima S, editor. PLoS ONE. 2023;18(9):e0286681.
269. Brooks GA, Clark L. The gamblers of the future? Migration from loot boxes to gambling in a longitudinal study of young adults. Computers in Human Behavior. 2023;141:107605.
270. González-Cabrera J, Basterra-González A, Ortega-Barón J, Caba-Machado V, Díaz-López A, Pontes HM, et al. Loot box purchases and their relationship with internet gaming disorder and online gambling disorder in adolescents: A prospective study. Computers in Human Behavior. 2023;143:107685.
271. Close J, Spicer SG, Nicklin LL, Uther M, Whalley B, Fullwood C, et al. Exploring the relationships between psychological variables and loot box engagement, part 1: pre-registered hypotheses. R Soc Open Sci. 2023;10(12):231045.
272. Duarte LFC, Ishikawa E. Loot box gambling addiction risk versus responsible computing: a systematic review. JIS. 2023;14(1):106–18.
273. Sirola A, Nyrhinen J, Nuckols J, Wilska TA. Loot box purchasing and indebtedness: The role of psychosocial factors and problem gambling. Addictive Behaviors Reports. 2023;18:100516.
274. Oelker A, Rumpf HJ, Brand M, Müller SM. Validation of the ACSID-11 for consistent screening of specific Internet-use disorders based on ICD-11 criteria for gaming disorder: A multitrait-multimethod  approach. Compr Psychiatry. 2024;132:152470.
275. Hunt A, Merola GP, Carpenter T, Jaeggi AV. Evolutionary perspectives on substance and behavioural addictions: Distinct and shared pathways to understanding, prediction and prevention. Neurosci Biobehav Rev. 2024;159:105603.
276. Saini N, Adair C, King DL, Kuss DJ, Gentile DA, Kim HS, et al. Development of the Saini-Hodgins Addiction Risk Potential of Games (SHARP-G) Scale: An International Delphi study. J Behav Addict. 2024;13(2):450–62.
277. Inaguma T, Misumi S, Funatogawa T, Nemoto T, Harima H, Mizuno M. Does the loot box open the door to addiction? A case report of gaming disorder with high charges for loot box purchases. PCN Rep. 2024;3(1):e167.
278. Schumacher J, Puhm A, Romic M, Mardešić M, Petrenko R, Lainas S, et al. Risks and harms associated with online gaming and gambling [Internet]. Strasbourg: Council of Europe International Cooperation Group on Drugs and Addictions; 2024 [cited 2024 Sep 20]. Available from: <https://www.drugsandalcohol.ie/41261/>
279. Popović L. Esports as a phenomenon of new trends in tourism : a gaming experience through digital and live events [Internet] [Masters]. [Croatia]: University of Zagreb; 2023 [cited 2024 Sep 20]. Available from: <https://urn.nsk.hr/urn:nbn:hr:148:829528>
280. Balahmar NB. The Gamers’ Phenomenon among Saudi Young Population as an Active Audience [Internet] [Doctoral]. [United Kingdom]: University of Leicester; 2024 [cited 2024 Sep 20]. Available from:

https://figshare.le.ac.uk/articles/thesis/The_Gamers_Phenomenon_among_Saudi_Young_Population_as_an_Active_Audience/26318965/1

1. Błoch M, Misiak B. Understanding the Emergence of Comorbidity between Problematic Online Gaming and Gambling: A Network Analysis Approach. Brain Sciences. 2024;14(9):929.
2. González-Cabrera J, Caba-Machado V, Díaz-López A, Jiménez-Murcia S, Mestre-Bach G, Machimbarrena JM. The Mediating Role of Problematic Use of Loot Boxes Between Internet Gaming Disorder and Online Gambling Disorder: Cross-Sectional Analytical Study. JMIR Serious Games. 2024;12:e57304–e57304.
3. Imataka G, Izumi S, Miyamoto Y, Maehashi A. Gaming Disorders: Navigating the Fine Line between Entertainment and Addiction—Gaming History, Health Risks, Social Consequences, and Pathways to Prevention. J Clin Med. 2024;13(17):5122.
4. Suriá-Martínez R, García-Castillo F, López-Sánchez C, Villegas E, Carretón C. Online Games and Cognitive Distortions: A Comparative Analysis in Students with and without Disabilities. EJIHPE. 2024;14(7):1868–80.
5. Spicer SG, Close J, Nicklin LL, Uther M, Whalley B, Fullwood C, et al. Exploring the relationships between psychological variables and loot box engagement, part 2: exploratory analyses of complex relationships. R Soc open sci. 2024;11(1):231046.
6. Mrayati OM. Assessing the prevalence and perception of video games loot box engagement and its association with problematic gaming behaviors among the Saudi community [Internet] [Masters]. [Saudi Arabia]: Alfaisal University; 2024 [cited 2024 Sep 20]. Available from: <https://www.proquest.com/docview/3067413534>
7. Kesuma AE, Princes E. Antecedents of Gacha gaming intention: Extending UTAUT2 with structural video game characteristics. Computers in Human Behavior Reports. 2024;14:100405. 1.
8. Macey J, Hamari J, Adam M. A conceptual framework for understanding and identifying gamblified experiences. Computers in Human Behavior. 2024;152:108087.
9. Xiao LY, Henderson LL, Yang Y, Newall PWS. Gaming the system: suboptimal compliance with loot box probability disclosure regulations in China. Behav Public Policy. 2024;8(3):590–616.
